# Efficacy of UB0316, a multi-strain probiotic formulation in patients with type 2 diabetes mellitus: A double blind, randomized, placebo controlled study

**DOI:** 10.1371/journal.pone.0225168

**Published:** 2019-11-13

**Authors:** Ratna Sudha Madempudi, Jayesh J. Ahire, Jayanthi Neelamraju, Anirudh Tripathi, Satyavrat Nanal

**Affiliations:** 1 Centre for Research & Development, Unique Biotech Ltd., Alexandria Knowledge Park, Hyderabad, Telangana, India; 2 Life Veda Treatment and Research Centre, Worli, Mumbai, India; 3 Nanal Clinic, Anand Bhuvan, Gore wadi, Mahim (W), Mumbai, India; Universidad Miguel Hernandez de Elche, SPAIN

## Abstract

**Background:**

Role of multi-strain probiotic formulations in the management of type 2 diabetes mellitus (T2DM) has rarely been reported. In the present study, the effects of the probiotic formulation, UB0316 (*L*. *salivarius* UBLS22, *L*. *casei* UBLC42, *L*. *plantarum* UBLP40, *L*. *acidophilus* UBLA34, *B*. *breve* UBBr01, *B*. *coagulans* Unique IS2, 5 billion CFU each and fructo-oligosaccharides, 100 mg) in patients with T2DM were assessed.

**Methods:**

A total of 79 eligible subjects (18–65 years, on stable metformin therapy) were randomly assigned to receive UB0316 or placebo, twice-a-day for 12 weeks. The primary endpoint was change in glycated hemoglobin (HbA1c), secondary were assessment of blood glucose levels, HOMA-IR (homeostatic model assessment of insulin resistance), insulin, body weight, and blood lipids. Quality of life, vital signs, physical investigations, safety and Physician/Subject’s Global assessment were also evaluated.

**Results:**

Twelve week multi-strain probiotic (UB0316) supplementation significantly reduced HbA1c (7.70 ± 0.79%; *p* = 0.0023) and weight (67.00 ± 8.82 kg; *p* < 0.001) as compared to placebo (HbA1c: 8.30 ± 1.35%; weight: 67.60 ± 9.46 kg). The changes recorded in fasting blood glucose (FBG), HOMA-IR, insulin, TC, TG, HDL, and LDL levels were however not significantly altered as compared to placebo. No severe adverse events, abnormal vital and physical signs were reported. The quality of life of T2DM was significantly improved.

**Conclusions:**

UB0316 significantly improved glycemic control as indicated by the decrease in HbA1c levels. There was also a significant decrease in weight in the probiotic treated subjects as compared to placebo.

## Introduction

Type 2 diabetes mellitus (T2DM), a non-communicable metabolic disease characterized by high blood glucose levels, results from impaired insulin secretion, insulin resistance or a combination of both [1]. According to the International Diabetes Federation (IDF), In 2017, 98 million adults aged 65–79 years and 327 million adults aged 20–64 worldwide were diabetic. This number is expected to increase to 191 million (age 65–79 years) and 438 million (age 20–64) by 2045 [2]. T2DM accounts for more than 90% of all diagnosed diabetes cases and is largely the result of excess body weight and physical inactivity [1–3]. The current treatment of T2DM is costly, limited and based on management of insulin resistance by medication and life style changes. There is a significant need for research on cost effective therapies that not only control blood glucose and insulin resistance but also improve patients’ quality of life, decrease future treatment burden and serve to stabilize metabolic control [4].

Recent research on gut microbiota suggests that the bacterial community profile is a major contributing factor towards the development of T2DM, besides genetic and environmental factors [1, 5]. Significantly lower relative abundance of *Firmicutes* and higher proportions of *Bacteroidetes* and *Proteobacteria* are found in T2DM persons compared to non-diabetic counterparts [6]. The lipopolysaccharide (LPS) of enriched Gram-negative bacteria belonging to the phyla *Bacteroidetes* and *Proteobacteria* are known to trigger inflammation and play an important role in the development of T2DM [7]. In recent years, gut microbiota manipulations using probiotics is garnering a lot of interest in the control of diabetes [8]. Evidence suggests that probiotics control gut dysbiosis, improve barrier function, insulin sensitivity and reduce chronic systemic inflammations [9]. Probiotics are “live microorganisms that, when administered in adequate amounts, confer a health benefit on the host” [10]. Probiotic strains which have been implicated in regulation of sugar levels include *Lactobacillus acidophilus*, *L*. *casei*, *L*. *rhamnosus*, *L*. *bulgaricus*, *L*. *lactis*, *Bifidobacterium breve*, *B*. *longum*, *B*. *infantis*, *B*. *lactis*, *Streptococcus thermophilus* and *Bacillus coagulans* (*L*. *sporogenes*) [11].

Metformin is the first-line treatment for T2DM, known to increase glucose uptake by peripheral tissues and reduce hepatic glucose production [12]. These kind of glucose-lowering agents are known to affect the composition and diversity of gut microbiota [12, 13]. Forslund and co-workers suggest partial gut microbiota mediation during therapy with metformin [14]. In this study, we have combined multi-strains (*L*. *salivarius* UBLS22, *L*. *casei* UBLC42, *L*. *plantarum* UBLP40, *L*. *acidophilus* UBLA34, *B*. *breve* UBBr01, *B*. *coagulans* Unique IS2, 5 billion CFU each and fructo-oligosaccharides, 100 mg), which are well documented for their *in vivo* clinical efficiencies to improve cholesterol profile (*B*. *coagulans* Unique IS2 and *L*. *salivarius* UBLS22) [15, 16], reduction in inflammatory markers (*L*. *salivarius* UBLS22) [16], complications of liver cirrhosis (*B*. *coagulans* Unique IS2) [17], irritable bowel syndrome in childrens [18] and adults (*B*. *coagulans* Unique IS2) [19], childhood abdominal pain (*B*. *coagulans* Unique IS2) [20], acute diarrhoea and constipation (*B*. *coagulans* Unique IS2) [21, 22] and improvement in the health-related quality of life in individuals with type II diabetes mellitus (*L*. *salivarius* UBLS22, *L*. *casei* UBLC42, *L*. *plantarum* UBLP40, *L*. *acidophilus* UBLA34, *B*. *breve* UBBr01, *B*. *coagulans* Unique IS2) [23]. The multi-strain capsule is referred as UB0316 and in this study, the efficacy on HbA1c and blood glucose levels, HOMA-IR, weight, blood lipid profile and quality of life in T2DM adults on stable metformin therapy were assessed.

## Methods

This randomized, double blind, placebo-controlled, twelve week clinical trial was reviewed and approved by Intersystem Biomedica Ethics Committee (ISBEC/NR20/KM-JVJ/2016) on 19^th^ July 2016 and performed in accordance with recommendation on Good clinical practice (GCP, 2016), Declaration of Helsinki (2013) and Indian Council of Medical Research (2006). The participants were recruited and enrolled at two sites i.e. Nanal Clinic (Mumbai, India) and Life Veda Treatment and Research Centre (Mumbai, India). All the participants signed informed consent prior to their inclusion in the study. This trial was registered retrospectively with Clinical Trial Registry of India (http://ctri.nic.in) with registration number CTRI/2017/07/009164. The reason for the delay in registering at CTRI was due to some internet connectivity issues and technical hitches. The authors confirm that all ongoing and related trials for this drug/intervention are registered.

### Participants

Participants of either sex, age 18−65 years were screened as per inclusion and exclusion criteria. *Inclusion criteria*: Type 2 diabetics (on stable metformin (500 mg) monotherapy for 8 weeks prior to the screening); BMI between 23 to 32 kg/m^2^; glycated hemoglobin (HbA1c) between 7–9%; non pregnant females and those willing to sign informed consent form prior to participation.

*Exclusion criteria*: Type 1 diabetic; history of diabetic ketoacidosis; using anti-hyperglycemic medication other than metformin; fasting blood triglycerides > 400 mg/dL and/or low density lipoprotein (LDL) > 190 mg/dL; HbA1c, > 9%; known hypersensitivity to study drugs or constituents; severe systemic disease; on ayurvedic, homeopathic or herbal medicines. The participants were asked to refrain from consuming yoghurt or other similar dietary supplements during the study, no changes were made in the dietary pattern and life style of the subjects.

*Discontinuation criteria*: Participants were free to drop out during the study and could choose not to receive intervention; in the event of adverse reactions and laboratory abnormalities; failure to comply visit requirements as per protocol; pregnancy; use of prohibited medication; worsening of condition/disorder; frequent non-adherence to dosing regimen.

### Intervention and compliance

Multi-strain probiotic UB0316 capsules (*L*. *salivarius* UBLS22, *L*. *casei* UBLC42, *L*. *plantarum* UBLP40, *L*. *acidophilus* UBLA34, *B*. *breve* UBBr01, and *B*. *coagulans* Unique IS2, 30 billion CFU and fructo-oligosaccharide, 100 mg) and placebo capsules (containing excipient maltodextrin) were provided by Unique Biotech Limited (Hyderabad, India). Participants were instructed to take 2 capsules (UB0316 or placebo) daily after any principal meal for up to 12 weeks. During the intervention, participants were called for 3 visits at the interval of 4 weeks from baseline and assessed for schedule study outcomes. Compliance was monitored by recording the data on used (empty bottles) and unused capsules at respective study sites. All the participants were on metformin (500 mg) during the trial.

### Primary outcome

Primary outcome of the present clinical investigation was the change in glycated hemoglobin (HbAlc) from baseline to week 12. HbAlc concentration (mmol/mol) in blood was evaluated by Tina-quant^®^ HbA1c assay using Roche cobas^®^ 6000 modular analyzer series (Rotkreuz, Switzerland) and expressed in percent (%).

### Secondary outcomes

#### Blood glucose

Fasting blood samples of participants were collected by standard pathological procedure and glucose levels were evaluated by GOD-POD (glucose oxidase−peroxidase) method using Roche cobas^®^ 6000 modular analyzer series (Rotkreuz, Switzerland). The blood glucose levels (mg/dL) were estimated at baseline and week 12.

#### Homeostatic Model Assessment of Insulin Resistance (HOMA-IR) and Insulin

HOMA-IR was calculated based on the concentrations of fasting blood glucose and insulin. A computer-solved model was used to predict homeostatic concentrations arising from varying degrees of beta cell deficiency and insulin resistance. Serum insulin levels were measured by chemi-luminescent immunoassay (CLIA) using Roche, Elecsys 2010 Immunology analyzer (Rotkreuz, Switzerland). HOMA-IR and insulin levels were assessed at baseline and week 12.

#### Body weight

Participant’s body weight was measured on calibrated Omron, HBF-362, electronic scale (Omron, Karada scan, Netherlands) with an accuracy of ± 1% (kg), at baseline and week 12.

#### Fasting blood lipids

Fasting blood samples were collected from participants and evaluated for total cholesterol (TC: CHOD-PAP method), triglycerides (TG: GPO-PAP method), high density lipoprotein (HDL: direct enzymatic method), and low density lipoproteins (LDL: direct enzymatic method/calculated) at baseline and week 12.

### Quality of life (QOL)

QOL was assessed according to the questionnaire consisting of eight domains *viz*. physical health, physical endurance, general health, treatment satisfaction, symptom botherness, financial worries, emotional/mental health and diet satisfaction [24], on baseline and scheduled visits.

### Physician and subject global assessment

The assessment was performed on the basis of a self-administered scale which was completed by the subject or his caregiver and physician during the visits at week 4, 8 and 12. The scale of 1 indicates “worsening of condition” and 5 indicates “comprehensive relief” as to how severely activities were hindered due to diabetes.

### Safety measures

Safety was measured by recording the incidence of adverse events (as per MedDRA), their severity (mild, moderate, severe) and relationship (not related, unlikely, possibly, probably, definite) to the treatment. Simultaneously, vital signs (heart rate, respiratory rate, blood pressure and temperature), physical examination and laboratory investigations (hematology) were performed at appropriate visits.

#### Sample size determination

SAS software (version 9.4) was used to determine sample size required to evaluate primary end point (HbA1c). Approximately, 92 participants were required for screening and 70 for evaluation of primary outcome to give 80% power to reject the null hypothesis (H0 = UB0316 –placebo = 0 verses Ha = UB0316 − placebo ≠ 0), when the true overall mean difference is minimum 1.03 with a standard deviation of 1.5 at a significance level of 0.05. The primary null hypothesis is that there is no difference between the UB0316 and the placebo with respect to the mean change from baseline in Hb1Ac at week 12. Rejecting this null hypothesis at a 2-sided alpha of 0.05 level will indicate the difference observed between the UB0316 and placebo is statistically significant with respect to the mean change from baseline in Hb1Ac at week 12. In the present study, a total of 126 participants were screened, out of which 79 were enrolled and 74 completed all schedule visits.

### Randomization and blinding

Randomization was performed in a 1:1 ratio by using SAS software. The generated random numbers were provided to the investigators for drug dispensing, while identity of the participants was kept blind. The probiotic and placebo capsules were provided in identical bottles to ensure double blind condition, both the capsules were identical in shape, texture and appearance, except, placebo capsules lacked the active ingredients.

### Statistical analysis

Two sample *t*-test was performed to study the effect of probiotic treatment i.e. primary and secondary efficacy outcome measures, compared with placebo. The paired *t*-test was used to evaluate intragroup differences. The safety and efficacy endpoints were calculated with precision of 95% confidence interval (CI) using SAS software (Version 9.4, USA) and *p* value < 0.05 considered as significant.

## Results

### Participant flow

**Fig 1:**
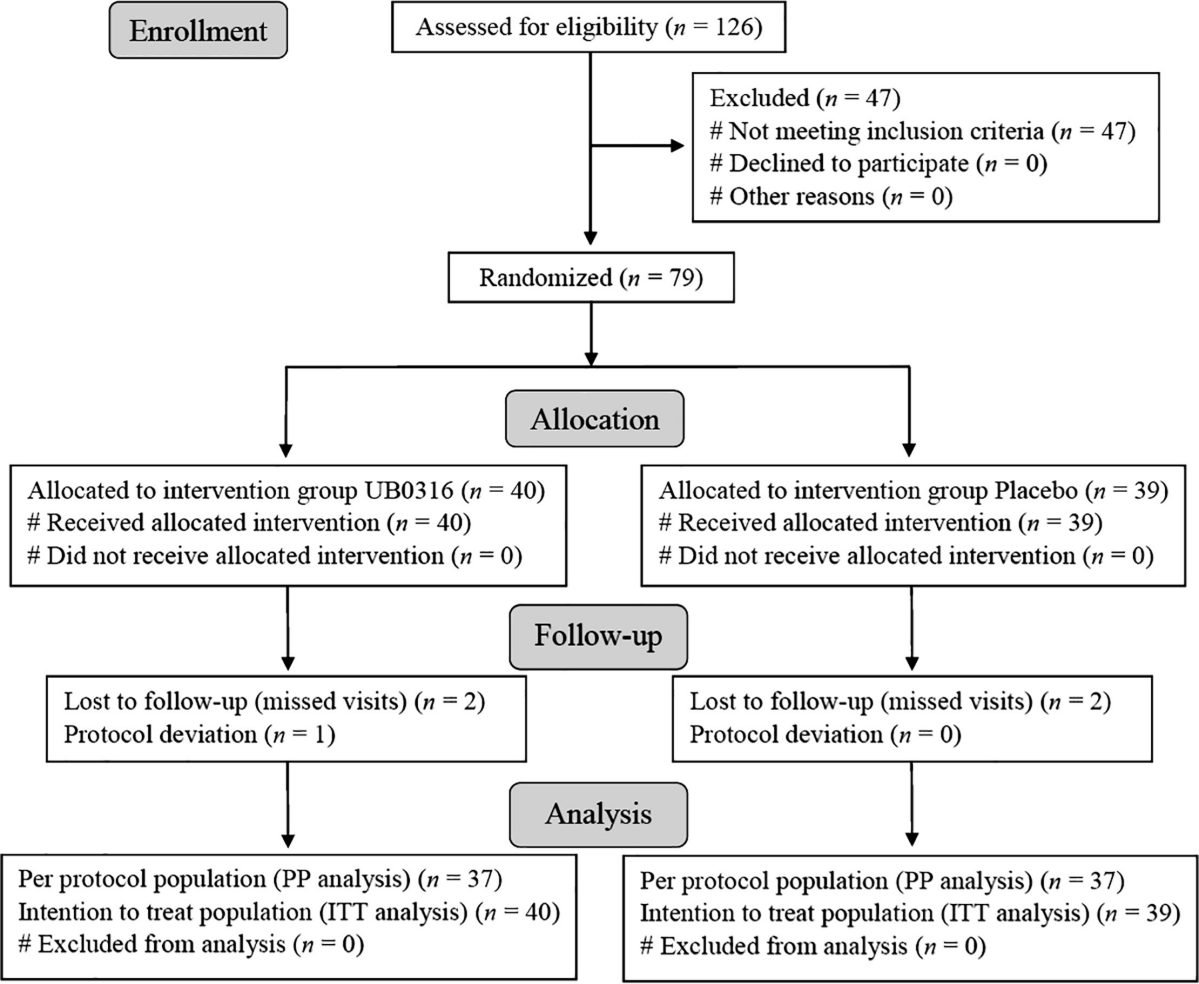
Participant flowchart of the trial.

One-twenty-six participants were screened for eligibility criteria, out of which 79 were enrolled and randomized between January 2017 and February 2018. The randomized participants who received intended treatment [UB0316 (*n* = 40) or placebo (*n* = 39)] and analyzed after baseline, were included in Intention to Treat (ITT) population (*n* = 79). Four participants [UB0316 (*n* = 2) and placebo (*n* = 2)] dropped out due to unavailability at scheduled visits, and 1 was a protocol deviation (UB0316 treatment) (Fig 1). The participants who completed all scheduled study visits were included in Per Protocol (PP) population [*n* = 74: UB0316 (*n* = 37) and placebo (*n* = 37)]. Both PP and ITT analysis were performed and ITT analysis was considered as primary to evaluate the effect of UB0316 intervention on primary and secondary outcomes of this study. The last observation carried forward (LOCF) method was employed in ITT analysis. The baseline demographics of UB0316 and placebo treated patients were comparable (Table 1). The measured treatment compliance was 100% in both UB0316 and placebo group. All the participants were on metformin (500 mg; concomitant) medication during the trial. Rescue medicines such as human insulin and glimepride were allowed, but not utilized during the trial. This trial was performed on participants of Indian origin.

**Table 1:** Baseline characteristics of participants in each group.

		UB0316	Placebo	Total
**Intention to Treat**	***n***	**40**	**39**	**79**
**Gender, *n***	male/female	33/7	29/10	62/17
**Age, years**	mean	54.10	50.60	52.40
**Height, cm**	mean	161.80	159.90	160.90
**Weight, kg**	mean	69.20	68.00	68.60
**HbA1c, %**	mean ± SD	8.20 ± 0.70	7.90 ± 0.72	8.10 ± 0.71
**FBG, mg/dL**	mean ± SD	147.60 ± 48.40	150.70 ± 41.30	149.20 ± 44.78
**HOMA-IR, units**	mean ± SD	3.00 ± 2.04	3.30 ± 1.86	3.10 ± 1.95
**Insulin, μlU/mL**	mean ± SD	8.70 ± 5.26	8.90 ± 4.51	8.80 ± 4.87
**TC, mg/dL**	mean ± SD	175.10 ± 35.01	173.90 ± 27.81	174.50 ± 31.47
**Triglycerides, mg/dL**	mean ± SD	161.70 ± 74.11	159.60 ± 77.19	160.60 ± 75.17
**HDL, mg/dL**	mean ± SD	49.50 ± 6.30	49.30 ± 6.73	49.40 ± 6.48
**LDL, mg/dL**	mean ± SD	92.10 ± 34.13	93.60 ± 28.87	92.80 ± 31.45
**Per Protocol**	***n***	**37**	**37**	**74**
**Gender, *n***	male/female	30/7	28/9	58/16
**Age, years**	mean	53.60	50.50	52.10
**Height, cm**	mean	161.80	159.90	160.90
**Weight, kg**	mean	69.20	68.00	68.60
**HbA1c, %**	mean ± SD	8.20 ± 0.68	7.90 ± 0.72	8.00 ± 0.71
**FBG, mg/dL**	mean ± SD	150.30 ± 49.07	148.90 ± 40.16	149.60 ± 44.53
**HOMA-IR, units**	mean ± SD	3.10 ± 2.07	3.20 ± 1.86	3.20 ± 1.96
**Insulin, μlU/mL**	mean ± SD	9.00 ± 5.33	8.90 ± 4.61	8.90 ± 4.95
**TC, mg/dL**	mean ± SD	174.30 ± 35.97	173.30 ± 28.14	173.80 ± 32.08
**Triglycerides, mg/dL**	mean ± SD	158.70 ± 68.87	162.50 ± 78.23	160.60 ± 73.22
**HDL, mg/dL**	mean ± SD	49.50 ± 6.37	49.40 ± 6.72	49.50 ± 6.50
**LDL, mg/dL**	mean ± SD	90.00 ± 34.50	92.40 ± 29.05	91.20 ± 31.69

HbA1c: glycated hemoglobin; FBG: Fasting blood glucose; HOMA-IR: homeostatic model assessment of insulin resistance; TC: total cholesterol; HDL: high density lipoproteins; LDL: low density lipoproteins.

### Primary outcome

#### Glycated hemoglobin (HbA1c) levels

**Fig 2:**
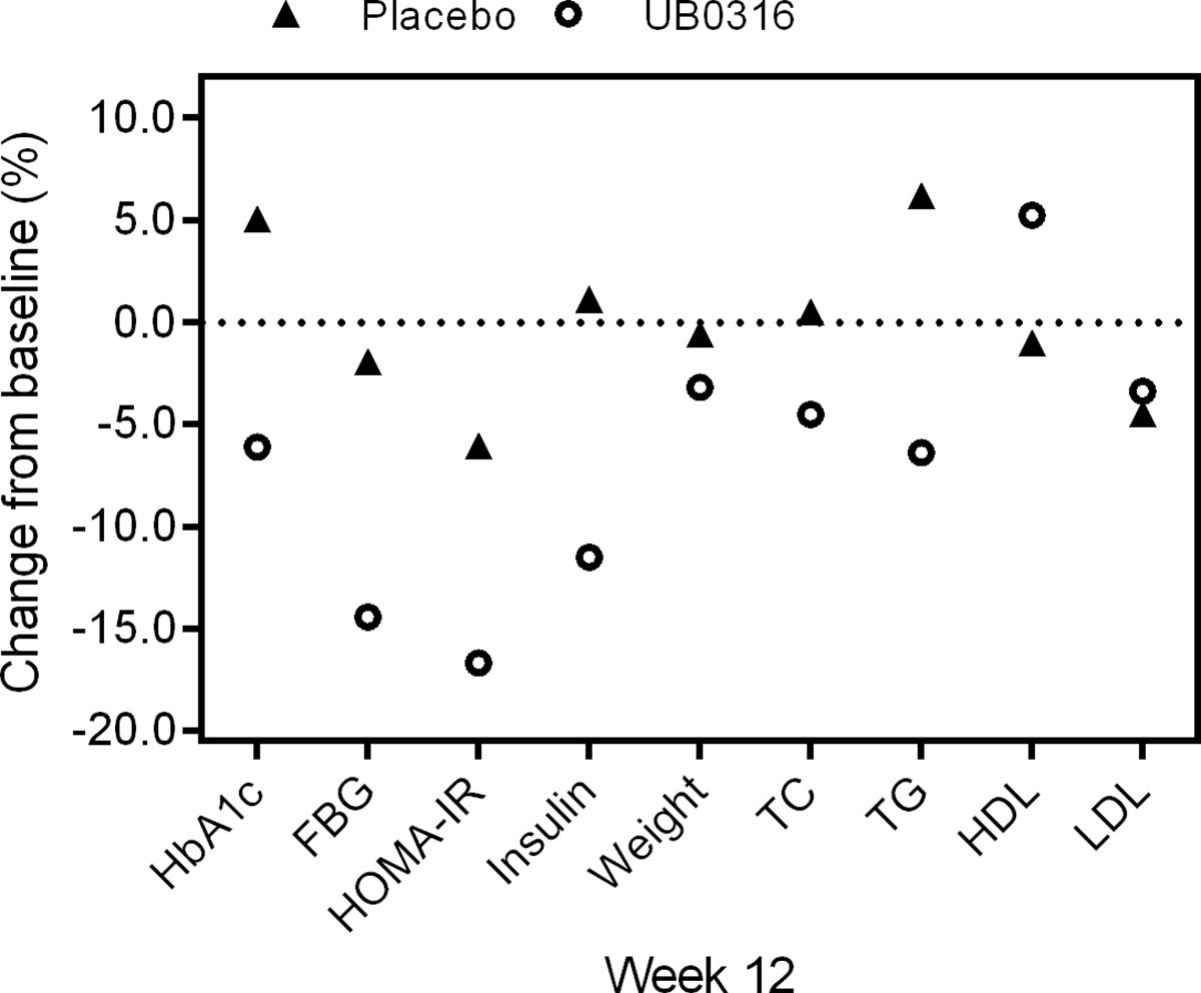
Effect of UB0316 and placebo on primary and secondary end points change (%) from baseline value (adjusted to zero) to week 12. HbA1c: glycated hemoglobin; FBG: fasting blood glucose; HOMA-IR: homeostatic model assessment of insulin resistance; TC: total cholesterol; TG: triglycerides; HDL: high density lipoproteins; LDL: low density lipoproteins. The percent change was calculated as, Mean _week 12_ –Mean _baseline_ / Mean _baseline_ × 100.

By the end of treatment (12 weeks), ITT population of UB0316 treatment (7.70 ± 0.79%) showed significant (*p* = 0.0150) reduction in HbA1c from the baseline (8.20 ± 0.70%), whereas placebo showed insignificant (*p* = 0.1878) increase of HbA1c (8.30 ± 1.35%) as compared to the baseline (7.90 ± 0.72%) (Table 2, Fig 2). The reduction in HbA1c with UB0316 treatment was significant (*p* = 0.0023), compared to placebo (Table 2). Similarly, in PP population, UB0316 supplementation significantly (*p* < 0.001) reduced HbA1c as compared to placebo (Table 2).

**Table 2:** Primary and secondary outcomes (baseline to week 12) of the Intention-to-Treat and Per Protocol populations.

	UB0316 (mean ± SD)		Placebo (mean ± SD)		Absolute change from baseline to visit (mean ± SD, 95% CI)	*p* value^#^	*p* value^§^	
	Baseline	Week 12	Baseline	Week 12			UB0316	Placebo
**Intention to Treat**	***n* = 40**	***n* = 40**	***n* = 39**	***n* = 39**	-	-	**-**	**-**
HbA1c, %	8.20 ± 0.70	7.70 ± 0.79	7.90 ± 0.72	8.30 ± 1.35	0.00 ± 1.09 (−0.19, −0.29)	0.0023	0.0150	0.1878
FBG, mg/dL	147.60 ± 48.40	126.30 ± 26.69	150.70 ± 41.30	147.80 ± 51.83	12.30 ± 45.51 (2.06, 22.45)	0.0709	0.0174	0.7855
HOMA-IR, units	3.00 ± 2.04	2.50 ± 1.24	3.30 ± 1.86	3.10 ± 1.38	0.40 ± 1.70 (−0.02, 0.74)	0.2944	0.1412	0.6702
Insulin, **μ**lU/mL	8.70 ± 5.26	7.70 ± 3.76	8.90 ± 4.51	9.00 ± 4.14	0.40 ± 4.25 (−0.51, 1.40)	0.2084	0.3126	0.8625
Weight, kg	69.20 ± 9.31	67.00 ± 8.82	68.00 ± 9.30	67.60 ± 9.46	1.00 ± 1.77 (0.55, 1.37)	< 0.001	0.3890	0.9673
TC, mg/dL	175.10 ± 35.01	167.20 ± 39.52	173.90 ± 27.81	174.80 ± 28.03	3.60 ± 34.83 (−4.24, 11.36)	0.2601	0.3452	0.8855
TG, mg/dL	161.70 ± 74.11	151.40 ± 64.10	159.60 ± 77.19	169.50 ± 86.99	0.30 ± 73.52 (−16.20, 16.74)	0.2246	0.5108	0.5946
HDL, mg/dL	49.50 ± 6.30	52.10 ± 9.23	49.30 ± 6.73	48.80 ± 7.16	−1.10 ± 9.50 (−3.23, 1.02)	0.1541	0.1440	0.7794
LDL, mg/dL	92.10 ± 34.13	89.00 ± 27.37	93.60 ± 28.87	89.40 ± 31.41	3.70 ± 30.64 (−3.21, 10.52)	0.8718	0.6552	0.5381
**Per Protocol**	***n* = 37**	***n* = 37**	***n* = 37**	***n* = 37**	-	-	-	-
HbA1c, %	8.20 ± 0.68	7.70 ± 0.78	7.90 ± 0.72	8.40 ± 1.28	0.00 ± 1.04 (−0.24, 0.24)	<0.001	0.0102	0.0731
FBG, mg/dL	150.30 ± 49.07	127.20 ± 26.97	148.90 ± 40.16	150.00 ± 51.90	11.00 ± 43.96 (0.83, 21.20)	0.0169	0.0150	0.9209
HOMA-IR, units	3.10 ± 2.07	2.50 ± 1.25	3.20 ± 1.86	3.20 ± 1.37	0.30 ± 1.69 (−0.06, 0.72)	0.1549	0.1312	0.9009
Insulin, **μ**lU/mL	9.00 ± 5.33	7.80 ± 3.77	8.90 ± 4.61	9.20 ± 4.20	0.40 ± 4.38 (−0.59, 1.43)	0.1686	0.2991	0.7832
Weight, kg	69.20 ± 9.31	67.40 ± 8.78	68.00 ± 9.30	67.90 ± 9.58	1.00 ± 1.77 (0.55, 1.37)	< 0.001	0.3928	0.9609
TC, mg/dL	174.30 ± 35.97	165.70 ± 40.46	173.30 ± 28.14	173.20 ± 27.94	4.30 ± 35.65 (−3.94, 12.58)	0.3088	0.3388	0.9919
TG, mg/dL	158.70 ± 68.87	147.60 ± 56.29	162.50 ± 78.23	172.60 ± 88.34	0.50 ± 75.98 (−17.13, 18.08)	0.2331	0.4517	0.6037
HDL, mg/dL	49.50 ± 6.37	52.40 ± 9.44	49.40 ± 6.72	48.60 ± 7.30	−1.00 ± 9.73 (−3.27, 1.24)	0.1105	0.1364	0.6292
LDL, mg/dL	90.00 ± 34.50	86.60 ± 26.90	92.40 ± 29.05	87.30 ± 30.88	4.20 ± 31.50 (−3.08, 11.51)	0.8151	0.6425	0.4682

HbA1c: glycated hemoglobin; FBG: fasting blood glucose; HOMA-IR: homeostatic model assessment of insulin resistance; TC: total cholesterol; TG: triglycerides; HDL: high density lipoproteins; LDL: low density lipoproteins.

#: intergroup (two sample *t* test)

§: intragroup (paired *t* test).

### Secondary outcomes

#### Blood glucose

In ITT population, UB0316 treatment by the end of 12 weeks significantly (*p* = 0.0174) reduced fasting (126.30 ± 26.69 mg/dL) blood glucose levels as compared to baseline (147.60 ± 48.40 mg/dL). (Table 2, Fig 2). Similarly, in the placebo group there was an insignificant reduction (*p* = 7855) in fasting sugar levels (147.80 ± 51.83 mg/dL) as compared to the baseline (150.70 ± 41.30 mg/dL), (Table 2, Fig 2). Moreover, the fasting blood glucose reduction with UB0316 treatment was insignificant (*p* = 0.0709), as compared to placebo. The analysis performed on PP population showed that UB0316 treatment significantly (*p* = 0.0169) reduce fasting blood glucose as compared with placebo (Table 2).

#### Homeostatic Model Assessment of Insulin Resistance (HOMA-IR) and Insulin

In ITT population, the HOMA-IR (2.50 ± 1.24 units) and insulin (7.70 ± 3.76 μlU/mL) levels were insignificantly (*p* = 0.1412; 0.3126) reduced from their baseline (3.00 ± 2.04 units; 8.70 ± 5.26 μlU/mL), when participants were treated with UB0316 for 12 week (Table 2, Fig 2). Similarly, placebo supplementation did not cause significant (*p* = 0.6702; 0.8625) change in either insulin (9.00 ± 4.14 μlU/mL) or HOMA IR (3.10 ± 1.38 units) levels as compared to the baseline (8.90 ± 4.51 μlU/mL; 3.30 ± 1.86 units) (Table 2, Fig 2). The HOMA-IR and insulin reduction observed with UB0316 treatment was however non-significant (*p* = 0.2944; *p* = 0.2084) when compared with placebo (Table 2). The PP analysis on PP population showed similar insignificant results (*p* = 0.1549; *p* = 0.1686) as compared to placebo (Table 2).

#### Body weight

In ITT population, UB0316 supplementation insignificantly (*p* = 0.3890) reduced body weight (kg) from 69.20 ± 9.31 (baseline) to 67.00 ± 8.82 (week 12) (Table 2, Fig 2). Similarly, placebo showed insignificant (*p* = 0.9673) reduction from 68.00 ± 9.30 to 67.60 ± 9.46 at week 12. However, the weight reduction observed with UB0316 was significant (*p* < 0.001) compared to the placebo (Table 2). Similar results were observed in PP analysis (Table 2).

#### Fasting blood lipids

At 12 weeks, the ITT participants of UB0316 treatment showed insignificant reduction in levels of total cholesterol (TC; 167.20 ± 39.52 mg/dL), triglycerides (TG; 151.40 ± 64.10 mg/dL), and low density lipoproteins (LDL; 89.00 ± 27.37 mg/dL) from the baseline (TC: 175.10 ± 35.01; TG: 161.70 ± 74.11; LDL: 92.10 ± 34.13 mg/dL), The levels of high density lipoprotein (HDL; 52.10 ± 9.23 mg/dL) were insignificantly increased from the baseline (49.50 ± 6.30 mg/dL) (Table 2, Fig 2). In placebo, TC (174.80 ± 28.03 mg/dL) and TG (169.50 ± 86.99 mg/dL) levels were increased and HDL (48.80 ± 7.16 mg/dL) and LDL (89.40 ± 31.41 mg/dL) were decreased from their respective baseline (TC: 173.90 ± 27.81; TG: 159.60 ± 77.19; HDL: 49.30 ± 6.73; LDL: 93.60 ± 28.87 mg/dL) (Table 2, Fig 2). The changes (intragroup) were non-significant. Overall, UB0316 treatment did not significantly improve fasting blood lipid profile (TC: *p* = 0.2601; TG: *p* = 0.2246; HDL: *p* = 0.1541; LDL: *p* = 0.8718) compared to placebo (Table 2). Similarly, in PP analysis, UB0316 supplementation failed to significantly improve fasting blood lipid profile (TC: *p* = 0.3088; TG: *p* = 0.2331; HDL: *p* = 0.1105; LDL *p* = 0.8151), as compared to placebo (Table 2).

### Quality of life (QOL)

**Fig 3:**
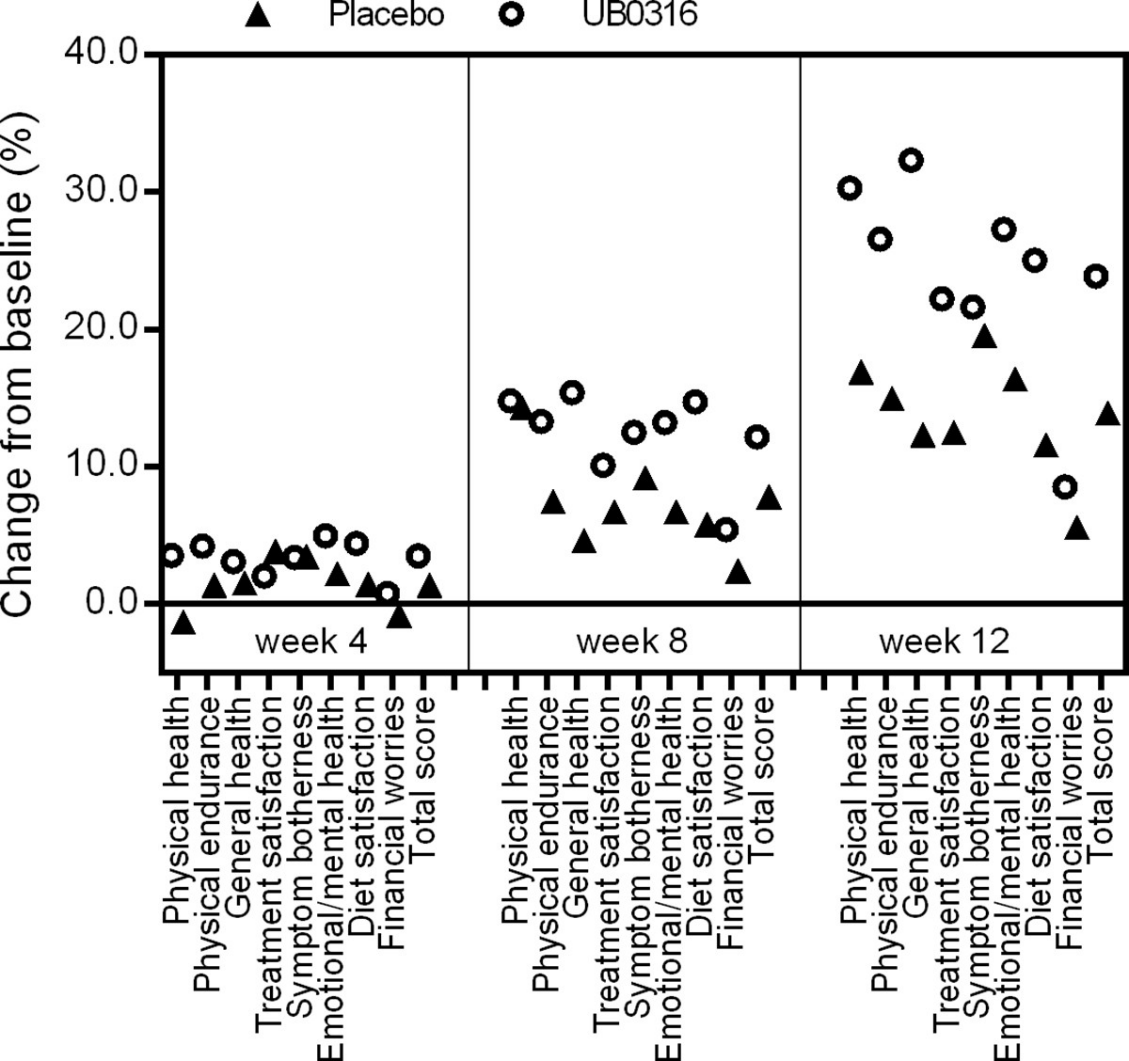
Effect of UB0316 and placebo on quality of life change (%) from baseline value (adjusted to zero) to week 4, 8 and 12. The percent change was calculated as, Mean _week_−Mean _baseline_ / Mean _baseline_ × 100.

The total score of QOL of ITT population was significantly improved with 12 weeks of UB0316 treatment (*p* = 0.0130), compared to placebo. Among the eight domains evaluated, five of the domains namely physical health (*p* = 0.0401), physical endurance (*p* = 0.0242), general health (*p* = 0.0062), treatment satisfaction (*p* = 0.0493), and diet satisfaction (*p* = 0.0352) improved significantly as compared with placebo. There was no significant change in the other three domains *viz*. symptom botherness (*p* = 0.6159), emotional/mental health (*p* = 0.0909) and financial worries (*p* = 0.3047) as compared to placebo (Table 3, Fig 3). However, symptom botherness and emotional/mental health were significantly (*p* < 0.001) improved from their baseline in UB0316 group (Table 3). The PP analysis, the total score of QOL was significant improved with UB0316 treatment (*p* = 0.0057) as compared to placebo (Table 3). The results of effect of treatment and placebo on QOL at all schedule visits are shown in S1 and S2 Tables.

**Table 3:** Quality of life assessment of the Intention-to-Treat and Per Protocol populations.

	UB0316 (mean ± SD)		Placebo (mean ± SD)		Absolute change from baseline to visit (mean ± SD, 95% CI)	*p* value^#^	*p* value^§^	
	Baseline	Week 12	Baseline	Week 12			UB0316	Placebo
**Intention to Treat**	***n* = 40**	***n* = 40**	***n* = 39**	***n* = 39**	-	-	-	-
**Physical health**	14.20 ± 5.33	18.50 ± 5.19	15.40 ± 4.43	18.00 ± 3.87	−3.50 ± 3.61 (−4.29, −2.67)	0.0401	<0.001	0.0064
**Physical endurance**	14.30 ± 5.74	18.10 ± 4.98	14.70 ± 5.17	16.90 ± 4.23	−3.00 ± 3.07 (−3.70, −2.33)	0.0242	0.0024	0.0403
**General health**	6.50 ± 2.08	8.60 ± 2.06	6.50 ± 1.64	7.30 ± 1.59	−1.50 ± 2.19 (−1.98, −1.00)	0.0062	<0.001	0.0275
**Treatment satisfaction**	9.90 ± 2.75	12.10 ± 2.58	10.40 ± 3.00	11.70 ± 2.33	−1.80 ± 2.14 (−2.24, −1.28)	0.0493	<0.001	0.0383
**Symptom botherness**	8.80 ± 2.17	10.70 ± 2.20	8.70 ± 1.98	10.40 ± 2.23	−1.80 ± 2.05 (−2.34, −1.33)	0.6159	<0.001	<0.001
**Emotional/mental health**	12.10 ± 4.98	15.40 ± 3.23	13.40 ± 4.87	15.60 ± 4.06	−2.70 ± 3.01 (−3.38, −2.03)	0.0909	<0.001	0.0394
**Diet satisfaction**	6.80 ± 2.24	8.50 ± 2.37	6.90 ± 2.42	7.70 ± 2.25	−1.20 ± 1.76 (−1.64, −0.85)	0.0352	0.0020	0.1250
**Financial worries**	12.90 ± 2.75	14.00 ± 2.23	12.50 ± 2.23	13.20 ± 2.32	−0.90 ± 1.76 (−1.27, −0.48)	0.3047	0.0584	0.2004
**Total score**	85.50 ± 23.53	105.90 ± 19.67	88.40 ± 20.30	100.70 ± 15.96	−16.40 ± 14.62 (−19.63, −13.08)	0.0130	<0.001	0.0040
**Per Protocol**	***n* = 37**	***n* = 37**	***n* = 37**	***n* = 37**	-	-	-	-
**Physical health**	14.00 ± 5.12	18.60 ± 5.04	15.10 ± 4.34	17.80 ± 3.83	−3.70 ± 3.65 (−4.51, −2.82)	0.0227	<0.001	0.0058
**Physical endurance**	14.10 ± 5.53	18.10 ± 4.77	14.40 ± 5.06	16.60 ± 4.11	−3.20 ± 3.10 (−3.88, −2.44)	0.0097	0.0011	0.0398
**General health**	6.50 ± 2.10	8.80 ± 2.03	6.40 ± 1.60	7.30 ± 1.63	−1.60 ± 2.20 (−2.12, −1.10)	0.0062	<0.001	0.0168
**Treatment satisfaction**	9.80 ± 2.77	12.20 ± 2.59	10.40 ± 3.04	11.70 ± 2.32	−1.90 ± 2.17 (−2.37, −1.36)	0.0309	<0.001	0.0386
**Symptom botherness**	8.70 ± 2.19	10.70 ± 2.24	8.80 ± 2.02	10.50 ± 2.22	−1.90 ± 2.07 (−2.37, −1.41)	0.5044	<0.001	<0.001
**Emotional/mental health**	11.90 ± 4.91	15.50 ± 2.99	13.10 ± 4.77	15.40 ± 4.08	−2.90 ± 3.01 (−3.60, −2.21)	0.0694	<0.001	0.0310
**Diet satisfaction**	6.60 ± 2.15	8.40 ± 2.39	6.80 ± 2.39	7.60 ± 2.24	−1.30 ± 1.80 (−1.73, −0.89)	0.0230	0.0012	0.1239
**Financial worries**	12.80 ± 2.66	14.00 ± 2.11	12.40 ± 2.28	13.10 ± 2.38	−0.90 ± 1.80 (−1.35, −0.52)	0.2754	0.0413	0.1990
**Total score**	84.40 ± 22.80	106.30 ± 18.90	87.20 ± 19.97	100.00 ± 15.94	−17.30 ± 14.58 (−20.72, −13.96)	0.0057	<0.001	0.0034

#:intergroup (two sample *t* test)

§: intragroup (paired *t* test)

### Physician and subject global assessment

Physician global assessment for UB0316 treatment on ITT and PP population between week 4 to 12 showed non-significant changes (*p* = 0.0780; *p* = 0.0769), compared to placebo (S3 Table). However, the changes observed in UB0316 group from the baseline were significant in both ITT and PP analysis (*p* < 0.001). The subject global assessment for UB0316 treatment on both ITT and PP population was significant (*p* = 0.0166; *p* = 0.0163) as compared to placebo (S4 Table). Similarly, the changes observed in UB0316 group from the baseline were significant in both ITT and PP analysis (*p* < 0.001).

In physician assessment on 12 week UB0316 supplementation, 2.50% participants of ITT population had complete relief, 55.00% considerable relief, 37.50% remained unchanged, 2.50% somewhat relieved and 2.50% experienced worse conditions. However, with placebo, 23.08% participants’ experienced considerable relief, 64.10% remained unchanged, 7.69% somewhat relieved and 5.13% worse (S5 Table). The assessment on PP population showed 59.46% participants had considerable relief, 35.14% remained unchanged, 2.70% somewhat relieved and 2.70% worse with UB0316 treatment, while 24.32% had considerable relief, 62.16% remained unchanged, 8.11% somewhat relieved and 5.41% worse (S5 Table).

In subject assessment (ITT population), 12 week UB0316 treatment provided complete relief to 2.50% participants while 52.50% had considerable relief, 40.00% remained unchanged, 2.50% somewhat relieved and 2.50% experienced worse conditions, whereas 23.08% participants showed considerable relief, 61.53% remained unchanged, 7.69% somewhat relieved and 7.69% experienced worse conditions with placebo supplementation (S6 Table). In PP population, 2.70% participants with UB0316 showed complete relief, 56.76% considerable relief, 35.14% remained unchanged, 2.70% somewhat relieved and 2.70% worse, whereas 24.32% participants with placebo had considerable relief, 59.46% remained unchanged, 8.11% somewhat relieved and 8.11% experienced worse conditions (S6 Table).

### Safety measures

During the twelve week UB0316 treatment, 2 participants (PP population) showed either constipation and or flatulence. According to MedDRA System Organ Class, these events are classified into gastrointestinal system organ class with preferred term of constipation and flatulence. Furthermore, the events were rated as mild (flatulence) and moderated (constipation), not related and assessed as unlikely relation to the treatment. Placebo had no adverse events to report. Hematology, vital signs and physical examinations remains normal and within normal range throughout the trial period (S7, S8 and S9 Tables). No other severe adverse events and deaths were reported in this study.

## Discussion

In this study, we have shown that the daily (twice a day) administration of UB0316 to T2DM patients (with HbA1c between 7–9% and on stable metformin: 500 mg) therapy for 12 weeks significantly improved HbA1c (primary outcome) along with a concomitant decrease in weight as compared to placebo. FBG, HOMA-IR, insulin, TC, TG, HDL, and LDL were however not -significant as compared to placebo. Overall, UB0316 significantly improved quality of life of T2DM patients.

The primary and secondary outcomes of the trial were evaluated for both ITT and PP population to avoid bias prone conclusions due to protocol deviations and dropouts [25]. The ITT analysis was considered as primary and at around 93.67% of ITT population was used to perform PP analysis.

HbA1c is an indicator of chronic glycaemia, which gives integrated glycemic index over the entire lifespan (120 day) of red blood cells [26]. Evidences suggest that probiotics control chronic glycaemia by stabilizing gut dysbiosis, improve barrier function, insulin sensitivity and reducing chronic systemic inflammations [9]. A recent clinical study conducted using a multi-strain probiotic formulation showed significant reduction in HbA1c after 8 weeks of treatment [27]. However, the trial was conducted on small population and there was no information on use of metformin which is considered as the first line of treatment. In the present 12 week clinical investigation, we have shown that multi-strain probiotic UB0316 as an adjuvant to metformin significantly reduced HbA1c. The change noted for placebo from the baseline was non-significant, which is in accordance to the results of Mobini et al. [28], that participants of placebo (most of them on metformin) showed no significant change in HbA1c for 12 weeks. Moreover, the 12 week trial with single strain probiotics did not lead to reduction in HbA1c in T2DM participants (on metformin). On contrary, 6 or 8 weeks studies using fermented products with two or more probiotic strains reported reductions in HbA1c, but there is no data available for metformin use during the trial [29–31]. Overall, the results suggested that multi-strain probiotics are much effective in reduction of HbA1c.

FBG levels estimated in ITT population of this study were non-significant may be due to LOCF. On the contrary, at 12 weeks, FBG levels in PP population were significantly (*p* = 0.0169) reduced with UB0316 treatment as compared with placebo. The FBG reduction is in accordance with recent meta-analysis that probiotic supplementation has significant FBG lowering effects when given along with antidiabetic drug in diabetic individuals [32].

Unbalanced blood lipid profile is one of the main symptoms of T2DM, which is known to intensify risk factors associated with cardiovascular diseases [33]. The participants recruited in this study had lipid profile values close to normal which could be due to the lipid balancing effects of metformin monotherapy [34]. Lipid profile changes in the UB0316 treatment group were insignificant as compared with placebo suggesting negligible effects of probiotics + metformin on lipid profile. However, in previous studies we have shown that daily supplementation of *B*. *coagulans* Unique IS2 for 60 days and *L*. *salivarius* UBLS-22 for 42 days significantly improve lipid profile of hypercholesterolemic [15] and or healthy individuals [16]. Moreover, the results of present investigation warrant need of clinical studies with large population, duration, customized diet and life style to come to a firm conclusion.

A recent, 12 week double blind, randomised, multi-strain probiotic trial conducted on T2DM patients showed that probiotic (*B*. *bifidum* W23, *B*. *lactis* W52, *L*. *acidophilus* W37, *L*. *brevis* W63, *L*. *casei* W56, *L*. *salivarius* W24, *Lactococcus lactis* W19 and W58, 2.5 × 10^9^ CFU/g; 2 g sachets) supplementation for 12 week significantly reduced HOMA-IR in medication naïve T2DM patients. The levels of insulin changed but remained insignificant as compared to placebo [35]. In the present study, we report multi-strain probiotic UB0316 supplementation for 12 weeks as an adjuvant to metformin reduced both HOMA-IR and insulin levels as compared to placebo in T2DM participants. Though, the changes were not significant, they appear important in maintaining a stable equilibrium between the conjuncted elements of T2DM, which are vital in the prevention and management of the diabetes.

Counteracting excessive weight gain is important to prevent T2DM [27]. However, there is no clear evidence that in patients with T2DM weight loss can reduce microvascular or cardiovascular complications [36, 37]. In this study, 12 week treatment of UB0316 significantly reduced weight of T2DM patients as compared with placebo, which is in agreement with the several previous findings [38]. However, the insignificant weight reduction observed in intragroup analysis warrant need of clinical studies with large population, duration, customized diet and life style.

T2DM has an impact on quality of life and socializing which may in turn may have negative impacts on mental health and long term T2DM management [39]. In the present investigation, we observed improvement in QOL of both probiotic and placebo treated groups with metformin, however, QOL scores were significantly higher in probiotic as compared to placebo. These results suggested that probiotic as an adjuvant to metformin improved QOL limitations associated with T2DM. Recently, Venkataraman et al. [23] showed that 12 week UB0316 capsule supplementation improved health-related quality of life in individuals with type II diabetes mellitus. Moreover, the improvement in scores of subject as well as physician global assessment suggested good tolerability profile of UB0316. There are however a few limitations to this study: Though sufficiently powered, the total number of subjects was not large; additionally the study duration was for only 12 weeks; no changes were made in the dietary pattern and life style of the subjects, which could be one of the reasons that no clear-cut significant differences in most of the secondary outcome measures between probiotic and placebo treatments were evident. Besides this, gut microbiota changes were not evaluated for the participants of both the groups.

## Conclusions

In conclusion, consumption of UB0316 along with metformin for a period of 12 weeks significantly lowered HbA1c and weight as compared to placebo with metformin. There was a trend towards improvement of FBG, HOMA-IR, insulin and lipid levels though it was not significant in T2DM patients. To conclude, UB0316 is safe and well-tolerated probiotic multi-strain formulation that can be given along with metformin which is used as the first line of treatment in the management of T2DM.

## Supporting information

S1 Table ITT analysis of change in Quality of Life (QOL) as compared from baseline. (DOCX)

S2 Table PP analysis of change in Quality of Life (QOL) as compared from baseline. (DOCX)

S3 Table Change from visit 1 to the end of visit 2 and visit 3 in physician global assessment of T2DM. (DOCX)

S4 Table Change from visit 1 to the end of visit 2 and visit 3 in subject’s global assessment of T2DM. (DOCX)

S5 Table Summary of physician global assessment of T2DM. (DOCX)

S6 Table Summary of subject’s global assessment of T2DM. (DOCX)

S7 Table Summary of hematology of T2DM participants. (DOCX)

S8 Table Summary of vital signs of T2DM participants. (DOCX)

S9 Table Summary of physical examination of T2DM participants. (DOCX)

S10 Table CONSORT 2010 Checklist. (DOC)

S1 Text Diabetes study protocol. (PDF)

## References

[1] DeFronzo RA, Ferrannini E, Groop L, Henry RR, Herman WH, Holst JJ, et al Type 2 diabetes mellitus. Nat Rev Dis Primers. 2015;1:15019 10.1038/nrdp.2015.19 27189025

[2] International Diabetes Federation (IDF). IDF Diabetes Atlas, 8th Edition, http://www.diabetesatlas.org (2017).

[3] World Health Organization (WHO). Diabetes. http://www.who.int/news-room/fact-sheets/detail/diabetes Accessed October 03, 2018.

[4] Inzucchi SE, Bergenstal RM, Buse JB, Diamant M, Ferrannini E, Nauck M, et al Management of hyperglycaemia in type 2 diabetes: a patient-centered approach. Position statement of the American Diabetes Association (ADA) and the European Association for the Study of Diabetes (EASD). Diabetologia. 2012;55:1577–1596. 10.1007/s00125-012-2534-0 22526604

[5] Han JL, Lin HL. Intestinal microbiota and type 2 diabetes: from mechanism insights to therapeutic perspective. World J Gastroenterol. 2014;20:17737–17745. 10.3748/wjg.v20.i47.17737 25548472 PMC4273124

[6] Larsen N, Vogensen FK, van den Berg FW, Nielsen DS, Andreasen AS, Pedersen BK, et al Gut microbiota in human adults with type 2 diabetes differs from non-diabetic adults. PloS One. 2010;5:e9085 10.1371/journal.pone.0009085 20140211 PMC2816710

[7] Allcock GH, Allegra M, Flower RJ, Perretti M. Neutrophil accumulation induced by bacterial lipopolysaccharide: effects of dexamethasone and annexin 1. Clin Exp Immunol. 2001;123:62–67. 10.1046/j.1365-2249.2001.01370.x 11167999 PMC1905950

[8] Sato J, Kanazawa A, Azuma K, Ikeda F, Goto H, Komiya K, et al Probiotic reduces bacterial translocation in type 2 diabetes mellitus: A randomised controlled study. Sci Rep. 2017;7:12115 10.1038/s41598-017-12535-9 28935921 PMC5608749

[9] Aw W, Fukuda S. Understanding the role of the gut ecosystem in diabetes mellitus. J Diabetes Investig. 2018;9:5–12. 10.1111/jdi.12673 28390093 PMC5754518

[10] Hill C, Guarner F, Reid G, Gibson GR, Merenstein DJ, Pot B, et al The international scientific association for probiotics and prebiotics consensus statement on the scope and appropriate use of the term probiotic. Nat Rev Gastroenterol Hepatol. 2014;11:506–514. 10.1038/nrgastro.2014.66 24912386

[11] Yao K, Zeng L, He Q, Wang W, Lei J, Zou X. Effect of probiotics on glucose and lipid metabolism in type 2 diabetes mellitus: a meta-analysis of 12 randomized controlled trials. Med Sci Monit. 2017;23:3044 10.12659/MSM.902600 28638006 PMC5491138

[12] Lv Y, Zhao X, Guo W, Gao Y, Yang S, Li Z, et al The relationship between frequently used glucose-lowering agents and gut microbiota in type 2 diabetes mellitus. J Diabetes Res. 2018; 1890978 10.1155/2018/1890978 29854817 PMC5964532

[13] Brunkwall L, Orho-Melander M. The gut microbiome as a target for prevention and treatment of hyperglycaemia in type 2 diabetes: from current human evidence to future possibilities. Diabetologia. 2017;60:943–951. 10.1007/s00125-017-4278-3 28434033 PMC5423958

[14] Forslund K, Hildebrand F, Nielsen T, Falony G, Le Chatelier E, Sunagawa S, et al Disentangling type 2 diabetes and metformin treatment signatures in the human gut microbiota. Nature. 2015;528:262–266. 10.1038/nature15766 26633628 PMC4681099

[15] Sudha MR, Radkar N, Maurya A. Effect of supplementation of probiotic *Bacillus coagulans* Unique IS-2 (ATCC PAT-11748) on hypercholesterolemic subjects: a clinical study. Int J Probiotics and Prebiotics. 2011;6:89–93.

[16] Rajkumar H, Kumar M, Das N, Kumar SN, Challa HR, Nagpal R. Effect of probiotic *Lactobacillus salivarius* UBL S22 and prebiotic fructo-oligosaccharide on serum lipids, inflammatory markers, insulin sensitivity, and gut bacteria in healthy young volunteers: a randomized controlled single-blind pilot study. J Cardiovasc Pharmacol Ther. 2015; 20:289–298. 10.1177/1074248414555004 25331262

[17] Pawar RR, Pardeshi ML, Ghongane BB. Study of effects of probiotic lactobacilli in preventing major complications in patients of liver cirrhosis. Int J Res Pharm Biomed Sci. 2012;3:206–211.

[18] Sudha MR, Jayanthi N, Aasin M, Dhanashri RD, Anirudh T. Efficacy of *Bacillus coagulans* Unique IS2 in treatment of irritable bowel syndrome in children: a double blind, randomised placebo controlled study. Benef Microbes. 2018;9:563–572. 10.3920/BM2017.0129 29695183

[19] Madempudi RS, Ahire JJ, Neelamraju J, Tripathi A, Nanal S. Randomized clinical trial: the effect of probiotic *Bacillus coagulans* Unique IS2 vs. placebo on the symptoms management of irritable bowel syndrome in adults. Sci Rep. 2019;9:12210 10.1038/s41598-019-48554-x 31434935 PMC6704184

[20] Saneian H, Pourmoghaddas Z, Roohafza H, Gholamrezaei A. Synbiotic containing *Bacillus coagulans* and fructo-oligosaccharides for functional abdominal pain in children. Gastroenterol Hepatol Bed Bench. 2015;8:56 25584177 PMC4285933

[21] Sudha RM, Bhonagiri S. Efficacy of *Bacillus coagulans* strain Unique IS-2 in the treatment of patients with acute diarrhea. Int J Probiotics and Prebiotics. 2012;7:33–37.

[22] Madempudi RS, Neelamraju J, Ahire JJ, Gupta SK, Shukla VK. *Bacillus coagulans* Unique IS2 in constipation: a double-blind, placebo-controlled study. Probiotics & Antimicro Prot. 2019;1–8. 10.1007/s12602-019-09542-9. 30911991

[23] Venkataraman R, Jose P, Jose J. Impact of probiotics on health-related quality of life in Type II diabetes mellitus: a randomized single-blind, placebo-controlled study. J Nat Sci Biol Med. 2019;10: 2 10.4103/jnsbm.JNSBM_31_18.

[24] Nagpal J, Kumar A, Kakar S, Bhartia A. The development of quality of life instrument for Indian diabetes patients (QOLID): a validation and reliability study in middle and higher income groups. J Assoc Physicians India. 2010;58:295–304. 21117348

[25] Clark GT, Mulligan R. Fifteen common mistakes encountered in clinical research. J Prosthodont Res. 2011;55:1–6. 10.1016/j.jpor.2010.09.002 21095178

[26] Florkowski C. HbA1c as a diagnostic test for diabetes mellitus–reviewing the evidence. Clin Biochem Rev. 2013;34:75–83. 24151343 PMC3799221

[27] Kobyliak N, Falalyeyeva T, Mykhalchyshyn G, Kyriienko D, Komissarenko I. Effect of alive probiotic on insulin resistance in type 2 diabetes patients: randomized clinical trial. Diabetes Metab Syndr: Clin Res Rev. 2018;12:617–624. 10.1016/j.dsx.2018.04.015 29661605

[28] Mobini R, Tremaroli V, Ståhlman M, Karlsson F, Levin M, Ljungberg M, et al Metabolic effects of *Lactobacillus reuteri* DSM 17938 in people with type 2 diabetes: a randomized controlled trial. Diabetes Obes Metab. 2017;19:579–589. 10.1111/dom.12861 28009106

[29] Mohamadshahi M, Veissi M, Haidari F, Javid AZ, Mohammadi F, Shirbeigi E. Effects of probiotic yogurt consumption on lipid profile in type 2 diabetic patients: a randomized controlled clinical trial. J Res Med Sci. 2014;19:531–536. 25197295 PMC4155708

[30] Ostadrahimi A, Taghizadeh A, Mobasseri M, Farrin N, Payahoo L, Gheshlaghi ZB, et al Effect of probiotic fermented milk (kefir) on glycemic control and lipid profile in type 2 diabetic patients: a randomized double-blind placebo-controlled clinical trial. Iran J Public Health. 2015;44:228–237. 25905057 PMC4401881

[31] Tonucci LB, dos Santos KM, de Oliveira LL, Ribeiro SM, Martino HS. Clinical application of probiotics in type 2 diabetes mellitus: a randomized, double-blind, placebo-controlled study. Clin Nutr. 2017;36:85–92. 10.1016/j.clnu.2015.11.011 26732026

[32] Ruan Y, Sun J, He J, Chen F, Chen R, Chen H. Effect of probiotics on glycemic control: a systematic review and meta-analysis of randomized, controlled trials. PloS One 2015;10:e0132121 10.1371/journal.pone.0132121 26161741 PMC4498615

[33] Ozder A. Lipid profile abnormalities seen in T2DM patients in primary healthcare in Turkey: a cross-sectional study. Lipids Health Dis. 2014;13:183 10.1186/1476-511X-13-183 25481115 PMC4271485

[34] Lin SH, Cheng PC, Te Tu S, Hsu SR, Cheng YC, Liu YH. Effect of metformin monotherapy on serum lipid profile in statin-naïve individuals with newly diagnosed type 2 diabetes mellitus: a cohort study. PeerJ. 2018;6:e4578 10.7717/peerj.4578 29666753 PMC5899882

[35] Sabico S, Al-Mashharawi A, Al-Daghri NM, Yakout S, Alnaami AM, Alokail MS, et al Effects of a multi-strain probiotic supplement for 12 weeks in circulating endotoxin levels and cardiometabolic profiles of medication naïve T2DM patients: a randomized clinical trial. J Transl Med. 2017;15:249 10.1186/s12967-017-1354-x 29228964 PMC5725828

[36] Chobot A, Górowska‐Kowolik K, Sokołowska M, Jarosz‐Chobot P. Obesity and diabetes–not only a simple link between two epidemics. Diabetes Metab Res Rev. 2018;34:e3042 10.1002/dmrr.3042 29931823 PMC6220876

[37] Wilding JPH. The importance of weight management in type 2 diabetes mellitus. Int J Clin Pract. 2014;68:682–691. 10.1111/ijcp.12384 24548654 PMC4238418

[38] John G, Wang L, Nanavati J, Twose C, Singh R, Mullin G. Dietary alteration of the gut microbiome and its impact on weight and fat mass: a systematic review and meta-analysis. Genes. 2018;9:167. 10.3390/genes9030167 PMC5867888 29547587

[39] Feng X, Astell-Burt T. Impact of a type 2 diabetes diagnosis on mental health, quality of life, and social contacts: a longitudinal study. BMJ Open Diabetes Res Care. 2017;5:e000198 10.1136/bmjdrc-2016-000198 28243446 PMC5316913

