# Supplementary material for: Efficacy of UB0316, a multi-strain probiotic formulation in patients with type 2 diabetes mellitus: A double blind, randomized, placebo controlled study
Source: PLoS One. 2019 Nov 13;14(11):e0225168. doi: 10.1371/journal.pone.0225168 (PMC6853318; doi:10.1371/journal.pone.0225168)
Supplement: S1 Table — (DOCX) [file pone.0225168.s001.docx]

**S1 Table.** **ITT analysis of change in Quality of Life (QOL) as compared from baseline.**

| **Visit** | **UB0316 (*n* = 40)** | | **Placebo (*n* = 39)** | | **Absolute change from baseline to visits** | | | ***p* value^#^** | ***p* value^§^** | |
| --- | --- | --- | --- | --- | --- | --- | --- | --- | --- | --- |
|  | **mean** | **SD** | **mean** | **SD** | **mean** | **SD** | **95% CI** |  | **UB0316** | **Placebo** |
| **Physical Health** | | | | | | | | | | |
| **Baseline** | 14.20 | 5.33 | 15.40 | 4.43 |  |  |  |  |  |  |
| **Week 4** | 14.70 | 4.98 | 15.20 | 3.78 | -0.10 | 1.99 | (-0.59,0.31) | 0.1616 | 0.6975 | 0.8479 |
| **Week 8** | 16.30 | 5.10 | 17.60 | 4.34 | -2.10 | 2.91 | (-2.78,-1.47) | 0.8148 | 0.0828 | 0.0293 |
| **Week 12** | 18.50 | 5.19 | 18.00 | 3.87 | -3.50 | 3.61 | (-4.29,-2.67) | 0.0401 | <0.001 | 0.0064 |
| **Physical Endurance** | | | | | | | | | | |
| **Baseline** | 14.30 | 5.74 | 14.70 | 5.17 |  |  |  |  |  |  |
| **Week 4** | 14.90 | 5.89 | 14.90 | 4.98 | -0.40 | 1.23 | (-0.67,-0.12) | 0.1833 | 0.6595 | 0.8588 |
| **Week 8** | 16.20 | 5.86 | 15.80 | 4.76 | -1.50 | 2.17 | (-2.01,-1.03) | 0.1410 | 0.1520 | 0.3084 |
| **Week 12** | 18.10 | 4.98 | 16.90 | 4.23 | -3.00 | 3.07 | (-3.70,-2.33) | 0.0242 | 0.0024 | 0.0403 |
| **General Health** | | | | | | | | | | |
| **Baseline** | 6.50 | 2.08 | 6.50 | 1.64 |  |  |  |  |  |  |
| **Week 4** | 6.70 | 1.90 | 6.60 | 1.48 | -0.20 | 1.15 | (-0.44,0.08) | 0.7127 | 0.6142 | 0.7178 |
| **Week 8** | 7.50 | 2.00 | 6.80 | 1.60 | -0.70 | 1.75 | (-1.11,-0.33) | 0.0905 | 0.0239 | 0.2969 |
| **Week 12** | 8.60 | 2.06 | 7.30 | 1.59 | -1.50 | 2.19 | (-1.98,-1.00) | 0.0062 | <0.001 | 0.0275 |
| **Treatment Satisfaction** | | | | | | | | | | |
| **Baseline** | 9.90 | 2.75 | 10.40 | 3.00 |  |  |  |  |  |  |
| **Week 4** | 10.10 | 2.60 | 10.80 | 2.80 | -0.30 | 1.22 | (-0.60,-0.06) | 0.4488 | 0.7078 | 0.5086 |
| **Week 8** | 10.90 | 2.53 | 11.10 | 2.65 | -0.90 | 1.60 | (-1.24,-0.53) | 0.3601 | 0.0795 | 0.2658 |
| **Week 12** | 12.10 | 2.58 | 11.70 | 2.33 | -1.80 | 2.14 | (-2.24,-1.28) | 0.0493 | <0.001 | 0.0383 |
| **Symptom Botherness** | | | | | | | | | | |
| **Baseline** | 8.80 | 2.17 | 8.70 | 1.98 |  |  |  |  |  |  |
| **Week 4** | 9.10 | 2.00 | 9.00 | 2.05 | -0.30 | 1.41 | (-0.60,0.04) | 0.7680 | 0.4875 | 0.6144 |
| **Week 8** | 9.90 | 2.08 | 9.50 | 1.90 | -0.90 | 1.94 | (-1.36,-0.49) | 0.4849 | 0.0264 | 0.0846 |
| **Week 12** | 10.70 | 2.20 | 10.40 | 2.23 | -1.80 | 2.05 | (-2.24,-1.33) | 0.6159 | <0.001 | <0.001 |
| **Emotional/Mental Health** | | | | | | | | | | |
| **Baseline** | 12.10 | 4.98 | 13.40 | 4.87 |  |  |  |  |  |  |
| **Week 4** | 12.70 | 4.50 | 13.70 | 4.79 | -0.40 | 1.34 | (-0.72,-0.12) | 0.4709 | 0.6223 | 0.7791 |
| **Week 8** | 13.70 | 3.82 | 14.30 | 4.26 | -1.20 | 2.07 | (-1.70,-0.78) | 0.1174 | 0.1107 | 0.4023 |
| **Week 12** | 15.40 | 3.23 | 15.60 | 4.06 | -2.70 | 3.01 | (-3.38,-2.03) | 0.0909 | <0.001 | 0.0394 |
| **Diet Satisfaction** | | | | | | | | | | |
| **Baseline** | 6.80 | 2.24 | 6.90 | 2.42 |  |  |  |  |  |  |
| **Week 4** | 7.10 | 2.43 | 7.00 | 2.47 | -0.20 | 0.69 | (-0.36,-0.05) | 0.2013 | 0.5676 | 0.8534 |
| **Week 8** | 7.80 | 2.29 | 7.30 | 2.29 | -0.70 | 1.27 | (-0.99,-0.42) | 0.0381 | 0.0521 | 0.4445 |
| **Week 12** | 8.50 | 2.37 | 7.70 | 2.25 | -1.20 | 1.76 | (-1.64,-0.85) | 0.0352 | 0.0020 | 0.1250 |
| **Financial Worries** | | | | | | | | | | |
| **Baseline** | 12.90 | 2.75 | 12.50 | 2.23 |  |  |  |  |  |  |
| **Week 4** | 13.00 | 2.60 | 12.40 | 2.12 | 0.00 | 0.53 | (-0.12,0.12) | 0.4002 | 0.9336 | 0.9176 |
| **Week 8** | 13.60 | 2.47 | 12.80 | 2.32 | -0.50 | 1.35 | (-0.76,-0.15) | 0.2607 | 0.2881 | 0.5863 |
| **Week 12** | 14.00 | 2.23 | 13.20 | 2.32 | -0.90 | 1.76 | (-1.27,-0.48) | 0.3047 | 0.0584 | 0.2004 |
| **Total Scores** | | | | | | | | | | |
| **Baseline** | 85.50 | 23.53 | 88.40 | 20.30 |  |  |  |  |  |  |
| **Week 4** | 88.30 | 22.37 | 89.60 | 18.18 | -2.00 | 6.52 | (-3.43,-0.52) | 0.2871 | 0.5937 | 0.7876 |
| **Week 8** | 95.90 | 21.10 | 95.30 | 18.15 | -8.60 | 9.89 | (-10.84,-6.40) | 0.1108 | 0.0412 | 0.1219 |
| **Week 12** | 105.90 | 19.67 | 100.70 | 15.96 | -16.40 | 14.62 | (-19.63,-13.08) | 0.0130 | <0.001 | 0.0040 |

*n*: number of participants

#: intergroup (two sample *t* test)

§: intragroup (paired *t* test)
