# Supplementary material for: Efficacy of UB0316, a multi-strain probiotic formulation in patients with type 2 diabetes mellitus: A double blind, randomized, placebo controlled study
Source: PLoS One. 2019 Nov 13;14(11):e0225168. doi: 10.1371/journal.pone.0225168 (PMC6853318; doi:10.1371/journal.pone.0225168)
Supplement: S2 Table — (DOCX) [file pone.0225168.s002.docx]

**S2 Table. PP analysis of change in Quality of Life (QOL) as compared from baseline.**

| **Visit** | **UB0316 (*n* = 37)** | | **Placebo (*n* = 37)** | | **Absolute change from baseline to visits** | | | ***p* value^#^** | ***p* value^§^** | |
| --- | --- | --- | --- | --- | --- | --- | --- | --- | --- | --- |
|  | **mean** | **SD** | **mean** | **SD** | **mean** | **SD** | **95% CI** |  | **UB0316** | **Placebo** |
| **Physical Health** | | | | | | | | | | |
| **Baseline** | 14.00 | 5.12 | 15.10 | 4.34 |  |  |  |  |  |  |
| **Week 4** | 14.50 | 4.78 | 15.00 | 3.76 | -0.20 | 1.97 | (-0.66,0.25) | 0.2639 | 0.6911 | 0.9545 |
| **Week 8** | 16.20 | 4.96 | 17.30 | 4.26 | -2.20 | 2.99 | (-2.90,-1.51) | 0.9693 | 0.0658 | 0.0298 |
| **Week 12** | 18.60 | 5.04 | 17.80 | 3.83 | -3.70 | 3.65 | (-4.51,-2.82) | 0.0227 | 0.0002 | 0.0058 |
| **Physical Endurance** | | | | | | | | | | |
| **Baseline** | 14.10 | 5.53 | 14.4 | 5.06 |  |  |  |  |  |  |
| **Week 4** | 14.70 | 5.74 | 14.6 | 4.87 | -0.40 | 1.27 | (-0.71,-0.12) | 0.1724 | 0.6366 | 0.8521 |
| **Week 8** | 16.10 | 5.74 | 15.5 | 4.60 | -1.60 | 2.22 | (-2.08,-1.05) | 0.0741 | 0.1264 | 0.3279 |
| **Week 12** | 18.10 | 4.77 | 16.6 | 4.11 | -3.20 | 3.10 | (-3.88,-2.44) | 0.0097 | 0.0011 | 0.0398 |
| **General Health** | | | | | | | | | | |
| **Baseline** | 6.50 | 2.10 | 6.40 | 1.60 |  |  |  |  |  |  |
| **Week 4** | 6.70 | 1.94 | 6.50 | 1.50 | -0.20 | 1.16 | (-0.47,0.07) | 0.9209 | 0.6473 | 0.6019 |
| **Week 8** | 7.60 | 2.03 | 6.80 | 1.63 | -0.80 | 1.77 | (-1.19,-0.37) | 0.1155 | 0.0241 | 0.2255 |
| **Week 12** | 8.80 | 2.03 | 7.30 | 1.63 | -1.60 | 2.20 | (-2.12,-1.10) | 0.0062 | <0.001 | 0.0168 |
| **Treatment Satisfaction** | | | | | | | | | | |
| **Baseline** | 9.80 | 2.77 | 10.40 | 3.04 |  |  |  |  |  |  |
| **Week 4** | 10.00 | 2.61 | 10.80 | 2.83 | -0.40 | 1.25 | (-0.64,-0.06) | 0.4630 | 0.6990 | 0.5034 |
| **Week 8** | 10.90 | 2.56 | 11.10 | 2.65 | -0.90 | 1.64 | (-1.31,-0.55) | 0.2912 | 0.0714 | 0.2750 |
| **Week 12** | 12.20 | 2.59 | 11.70 | 2.32 | -1.90 | 2.17 | (-2.37,-1.36) | 0.0309 | <0.001 | 0.0386 |
| **Symptom Botherness** | | | | | | | | | | |
| **Baseline** | 8.70 | 2.19 | 8.80 | 2.02 |  |  |  |  |  |  |
| **Week 4** | 9.00 | 2.02 | 9.00 | 2.07 | -0.30 | 1.46 | (-0.64,0.04) | 0.7526 | 0.4752 | 0.6106 |
| **Week 8** | 9.80 | 2.13 | 9.60 | 1.91 | -1.00 | 1.99 | (-1.43,-0.51) | 0.4186 | 0.0234 | 0.0902 |
| **Week 12** | 10.70 | 2.24 | 10.50 | 2.22 | -1.90 | 2.07 | (-2.37,-1.41) | 0.5044 | <0.001 | <0.001 |
| **Emotional/Mental Health** | | | | | | | | | | |
| **Baseline** | 11.90 | 4.91 | 13.10 | 4.77 |  |  |  |  |  |  |
| **Week 4** | 12.50 | 4.41 | 13.50 | 4.73 | -0.50 | 1.37 | (-0.78,-0.14) | 0.5007 | 0.6024 | 0.7512 |
| **Week 8** | 13.70 | 3.67 | 14.10 | 4.22 | -1.30 | 2.10 | (-1.82,-0.85) | 0.1105 | 0.0903 | 0.3688 |
| **Week 12** | 15.50 | 2.99 | 15.40 | 4.08 | -2.90 | 3.01 | (-3.60,-2.21) | 0.0694 | <0.001 | 0.0310 |
| **Diet Satisfaction** | | | | | | | | | | |
| **Baseline** | 6.60 | 2.15 | 6.80 | 2.39 |  |  |  |  |  |  |
| **Week 4** | 6.90 | 2.38 | 6.80 | 2.43 | -0.20 | 0.70 | (-0.37,-0.04) | 0.1382 | 0.5406 | 0.8853 |
| **Week 8** | 7.70 | 2.27 | 7.20 | 2.27 | -0.70 | 1.30 | (-1.05,-0.44) | 0.0254 | 0.0391 | 0.4561 |
| **Week 12** | 8.40 | 2.39 | 7.60 | 2.24 | -1.30 | 1.80 | (-1.73,-0.89) | 0.0230 | 0.0012 | 0.1239 |
| **Financial Worries** | | | | | | | | | | |
| **Baseline** | 12.80 | 2.66 | 12.40 | 2.28 |  |  |  |  |  |  |
| **Week 4** | 12.90 | 2.50 | 12.40 | 2.18 | -0.00 | 0.54 | (-0.14,0.11) | 0.5192 | 0.9285 | 0.9586 |
| **Week 8** | 13.50 | 2.38 | 12.70 | 2.38 | -0.50 | 1.39 | (-0.81,-0.17) | 0.2434 | 0.2535 | 0.5846 |
| **Week 12** | 14.00 | 2.11 | 13.10 | 2.38 | -0.90 | 1.80 | (-1.35,-0.52) | 0.2754 | 0.0413 | 0.1990 |
| **Total Scores** | | | | | | | | | | |
| **Baseline** | 84.40 | 22.80 | 87.20 | 19.97 |  |  |  |  |  |  |
| **Week 4** | 87.30 | 21.70 | 88.70 | 18.14 | -2.20 | 6.62 | (-3.72,-0.66) | 0.3464 | 0.5745 | 0.7431 |
| **Week 8** | 95.50 | 20.55 | 94.20 | 17.92 | -9.10 | 10.05 | (-11.40,-6.74) | 0.0727 | 0.0301 | 0.1183 |
| **Week 12** | 106.30 | 18.90 | 100.00 | 15.94 | -17.30 | 14.58 | (-20.72,-13.96) | 0.0057 | <0.001 | 0.0034 |

*n*: number of participants

#: intergroup (two sample *t* test)

§: intragroup (paired *t* test)
