# Supplementary material for: Efficacy of UB0316, a multi-strain probiotic formulation in patients with type 2 diabetes mellitus: A double blind, randomized, placebo controlled study
Source: PLoS One. 2019 Nov 13;14(11):e0225168. doi: 10.1371/journal.pone.0225168 (PMC6853318; doi:10.1371/journal.pone.0225168)
Supplement: S3 Table — (DOCX) [file pone.0225168.s003.docx]

**S3 Table.** **Change from visit 1 to the end of visit 2 and visit 3 in physician global assessment of T2DM.**

**ITT analysis**

| **Visit** | **UB0316 (*n* = 40)** | | **Placebo (*n* = 39)** | | **Absolute change from visit1 to visits** | | | ***p* value^#^** | ***p* value^§^** | |
| --- | --- | --- | --- | --- | --- | --- | --- | --- | --- | --- |
|  | **mean** | **SD** | **Mean** | **SD** | **mean** | **SD** | **95% CI** |  | **UB0316** | **Placebo** |
| **Visit 1 (Week 4)** | 3.10 | 0.22 | 2.90 | 0.27 |  |  |  |  |  |  |
| **Visit 2 (Week 8)** | 3.20 | 0.61 | 3.10 | 0.48 | -0.20 | 0.54 | -0.27 - -0.03 | 1.0000 | 0.1484 | 0.0869 |
| **Visit 3 (Week 12)** | 3.50 | 0.68 | 3.10 | 0.72 | -0.30 | 0.77 | -0.46 - -0.11 | 0.0780 | <0.001 | 0.3050 |

**PP analysis**

| **Visit** | **UB0316 (*n* = 37)** | | **Placebo (n = 37)** | | **Absolute change from visit1 to visits** | | | ***p* value^#^** | ***p* value^§^** | |
| --- | --- | --- | --- | --- | --- | --- | --- | --- | --- | --- |
|  | **mean** | **SD** | **mean** | **SD** | **mean** | **SD** | **95% CI** |  | **UB0316** | **Placebo** |
| **Visit 1 (Week 4)** | 3.10 | 0.23 | 2.90 | 0.28 |  |  |  |  |  |  |
| **Visit 2 (Week 8)** | 3.20 | 0.63 | 3.10 | 0.49 | -0.20 | 0.55 | -0.29, -0.03 | 1.0000 | 0.1479 | 0.0865 |
| **Visit 3 (Week 12)** | 3.50 | 0.69 | 3.10 | 0.74 | -0.30 | 0.79 | -0.48, -0.11 | 0.0769 | <0.001 | 0.3055 |

*n*: number of participants

#: inter group (two sample *t* test)

§: intra group (paired *t* test)
