# Supplementary material for: Efficacy of UB0316, a multi-strain probiotic formulation in patients with type 2 diabetes mellitus: A double blind, randomized, placebo controlled study
Source: PLoS One. 2019 Nov 13;14(11):e0225168. doi: 10.1371/journal.pone.0225168 (PMC6853318; doi:10.1371/journal.pone.0225168)
Supplement: S4 Table — (DOCX) [file pone.0225168.s004.docx]

**S4 Table. Change from visit 1 to the end of visit 2 and visit 3 in subject’s global assessment of T2DM.**

**ITT analysis**

| **Visit** | **UB0316 (*n* = 40)** | | **Placebo (*n* = 39)** | | **Absolute change from visit1 to visits** | | | ***p* value^#^** | ***p* value^§^** | |
| --- | --- | --- | --- | --- | --- | --- | --- | --- | --- | --- |
|  | **mean** | **SD** | **mean** | **SD** | **mean** | **SD** | **95% CI** |  | **UB0316** | **Placebo** |
| **Visit 1 (Week 4)** | 3.10 | 0.22 | 2.90 | 0.22 |  |  |  |  |  |  |
| **Visit 2 (Week 8)** | 3.20 | 0.63 | 3.10 | 0.48 | -0.20 | 0.56 | -0.28, -0.03 | 0.6887 | 0.0986 | 0.1366 |
| **Visit 3 (Week 12)** | 3.50 | 0.72 | 3.00 | 0.79 | -0.30 | 0.76 | -0.43, -0.08 | 0.0166 | <0.001 | 0.6999 |

**PP analysis**

| **Visit** | **UB0316 (*n* = 37)** | | **Placebo (*n* = 37)** | | **Absolute change from visit1 to visits** | | | ***p* value^#^** | ***p* value^§^** | |
| --- | --- | --- | --- | --- | --- | --- | --- | --- | --- | --- |
|  | **mean** | **SD** | **mean** | **SD** | **mean** | **SD** | **95% CI** |  | **UB0316** | **Placebo** |
| **Visit 1 (Week 4)** | 3.10 | 0.23 | 2.90 | 0.23 |  |  |  |  |  |  |
| **Visit 2 (Week 8)** | 3.20 | 0.64 | 3.10 | 0.49 | -0.20 | 0.57 | -0.30, -0.03 | 0.6884 | 0.0980 | 0.1369 |
| **Visit 3 (Week 12)** | 3.50 | 0.73 | 3.00 | 0.82 | -0.30 | 0.78 | -0.45, -0.09 | 0.0163 | <0.001 | 0.7002 |

*n*: number of participants

#: intergroup (two sample *t* test)

§: intragroup (paired *t* test)
