# Supplementary material for: Efficacy of UB0316, a multi-strain probiotic formulation in patients with type 2 diabetes mellitus: A double blind, randomized, placebo controlled study
Source: PLoS One. 2019 Nov 13;14(11):e0225168. doi: 10.1371/journal.pone.0225168 (PMC6853318; doi:10.1371/journal.pone.0225168)
Supplement: S6 Table — (DOCX) [file pone.0225168.s006.docx]

**S6 Table. Summary of subject’s global assessment of T2DM.**

**ITT analysis**

| **Categories** | **UB0316 (*n* = 40)** | | **Placebo (*n* = 39)** | |
| --- | --- | --- | --- | --- |
|  | **Visit 1, *n* (%)** | **Visit 3, *n* (%)** | **Visit 1, *n* (%)** | **Visit 3, *n* (%)** |
| **Complete relief** | 0 | 1 (2.50) | 0 | 0 |
| **Considerable relief** | 2 (5.00) | 21 (52.50) | 0 | 9 (23.08) |
| **Unchanged** | 38 (95.00) | 16 (40.00) | 37 (94.87) | 24 (61.53) |
| **Somewhat relieved** | 0 | 1 (2.50) | 2 (5.12) | 3 (7.69) |
| **Worse** | 0 | 1 (2.50) | 0 | 3 (7.69) |

**PP analysis**

| **Categories** | **UB0316 (*n* = 37)** | | **Placebo (*n* = 37)** | |
| --- | --- | --- | --- | --- |
|  | **Visit 1, *n* (%)** | **Visit 3, *n* (%)** | **Visit 1, *n* (%)** | **Visit 3, *n* (%)** |
| **Complete relief** | 0 | 1 (2.70) | 0 | 0 |
| **Considerable relief** | 2 (5.41) | 21 (56.76) | 0 | 9 (24.32) |
| **Unchanged** | 35 (94.59) | 13 (35.14) | 35 (94.59) | 22 (59.46) |
| **Somewhat relieved** | 0 | 1 (2.70) | 2 (5.41) | 3 (8.11) |
| **Worse** | 0 | 1 (2.70) | 0 | 3 (8.11) |
