# Supplementary material for: Efficacy of UB0316, a multi-strain probiotic formulation in patients with type 2 diabetes mellitus: A double blind, randomized, placebo controlled study
Source: PLoS One. 2019 Nov 13;14(11):e0225168. doi: 10.1371/journal.pone.0225168 (PMC6853318; doi:10.1371/journal.pone.0225168)
Supplement: S7 Table — (DOCX) [file pone.0225168.s007.docx]

**S7 Table. Summary of hematology of T2DM participants.**

|  | | **UB0316** | | | | | | **Placebo** | | | | | | **Total** | | | | | |
| --- | --- | --- | --- | --- | --- | --- | --- | --- | --- | --- | --- | --- | --- | --- | --- | --- | --- | --- | --- |
|  |  | **Normal** | | **Abnormal (CNS)** | | **Abnormal (CS)** | | **Normal** | | **Abnormal (CNS)** | | **Abnormal (CS)** | | **Normal** | | **Abnormal (CNS)** | | **Abnormal (CS)** | |
| **Test** | **Visit** | ***n*** | **%** | ***n*** | **%** | ***n*** | **%** | ***n*** | **%** | ***n*** | **%** | ***n*** | **%** | ***n*** | **%** | ***n*** | **%** | ***n*** | **%** |
| **PCV** | **Baseline** | 28 | 70.00 | 12 | 30.00 | 0 | 0 | 26 | 66.67 | 13 | 33.33 | 0 | 0 | 54 | 68.35 | 25 | 31.65 | 0 | 0 |
|  | **Week 12** | 25 | 67.56 | 12 | 32.43 | 0 | 0 | 25 | 67.56 | 12 | 32.43 | 0 | 0 | 50 | 67.57 | 24 | 32.43 | 0 | 0 |
| **MCV** | **Baseline** | 33 | 82.50 | 7 | 17.50 | 0 | 0 | 30 | 76.92 | 9 | 23.08 | 0 | 0 | 63 | 79.75 | 16 | 20.25 | 0 | 0 |
|  | **Week 12** | 27 | 72.97 | 10 | 27.02 | 0 | 0 | 27 | 72.97 | 10 | 27.02 | 0 | 0 | 54 | 72.97 | 20 | 27.03 | 0 | 0 |
| **MCH** | **Baseline** | 31 | 77.50 | 9 | 22.50 | 0 | 0 | 29 | 74.36 | 10 | 25.64 | 0 | 0 | 60 | 75.95 | 19 | 24.05 | 0 | 0 |
|  | **Week 12** | 25 | 67.56 | 12 | 32.43 | 0 | 0 | 26 | 70.27 | 11 | 29.72 | 0 | 0 | 51 | 68.92 | 23 | 31.08 | 0 | 0 |
| **MCHC** | **Baseline** | 23 | 57.50 | 17 | 42.50 | 0 | 0 | 29 | 74.36 | 10 | 25.64 | 0 | 0 | 52 | 65.82 | 27 | 34.18 | 0 | 0 |
|  | **Week 12** | 24 | 64.86 | 13 | 35.14 | 0 | 0 | 25 | 67.57 | 12 | 32.43 | 0 | 0 | 49 | 66.22 | 25 | 33.78 | 0 | 0 |
| **WBC** | **Baseline** | 38 | 95.00 | 2 | 5.00 | 0 | 0 | 37 | 94.87 | 2 | 5.13 | 0 | 0 | 75 | 94.94 | 4 | 5.06 | 0 | 0 |
|  | **Week 12** | 35 | 94.59 | 2 | 5.41 | 0 | 0 | 34 | 91.89 | 3 | 8.11 | 0 | 0 | 69 | 93.24 | 5 | 6.76 | 0 | 0 |
| **Neutrophils** | **Baseline** | 31 | 77.50 | 9 | 22.50 | 0 | 0 | 28 | 71.79 | 11 | 28.21 | 0 | 0 | 59 | 74.68 | 20 | 25.32 | 0 | 0 |
|  | **Week 12** | 27 | 72.97 | 10 | 27.02 | 0 | 0 | 22 | 59.46 | 15 | 40.54 | 0 | 0 | 49 | 66.21 | 25 | 33.79 | 0 | 0 |
| **Basophils** | **Baseline** | 39 | 97.50 | 1 | 2.50 | 0 | 0 | 38 | 97.44 | 1 | 2.56 | 0 | 0 | 77 | 97.47 | 2 | 2.53 | 0 | 0 |
|  | **Week 12** | 36 | 97.29 | 1 | 2.71 | 0 | 0 | 36 | 97.29 | 1 | 2.71 | 0 | 0 | 72 | 97.30 | 2 | 2.70 | 0 | 0 |
| **Eosinophil’s** | **Baseline** | 37 | 92.50 | 3 | 7.50 | 0 | 0 | 34 | 87.18 | 5 | 12.82 | 0 | 0 | 71 | 89.87 | 8 | 10.13 | 0 | 0 |
|  | **Week 12** | 32 | 86.49 | 5 | 13.51 | 0 | 0 | 31 | 83.78 | 6 | 16.22 | 0 | 0 | 63 | 85.14 | 11 | 14.86 | 0 | 0 |
| **Lymphocytes** | **Baseline** | 36 | 90.00 | 4 | 10.00 | 0 | 0 | 35 | 89.74 | 4 | 10.26 | 0 | 0 | 71 | 89.87 | 8 | 10.13 | 0 | 0 |
|  | **Week 12** | 34 | 91.89 | 3 | 8.11 | 0 | 0 | 33 | 89.19 | 4 | 10.81 | 0 | 0 | 67 | 90.54 | 7 | 9.46 | 0 | 0 |
| **Monocytes** | **Baseline** | 27 | 67.50 | 13 | 32.50 | 0 | 0 | 24 | 61.54 | 15 | 38.46 | 0 | 0 | 51 | 64.56 | 28 | 35.44 | 0 | 0 |
|  | **Week 12** | 24 | 64.86 | 13 | 35.13 | 0 | 0 | 25 | 67.56 | 12 | 32.43 | 0 | 0 | 49 | 66.21 | 25 | 33.79 | 0 | 0 |
| **Platelet Count** | **Baseline** | 36 | 90.00 | 4 | 10.00 | 0 | 0 | 33 | 84.62 | 6 | 15.38 | 0 | 0 | 69 | 87.34 | 10 | 12.66 | 0 | 0 |
|  | **Week 12** | 31 | 83.78 | 6 | 16.22 | 0 | 0 | 25 | 67.56 | 12 | 32.43 | 0 | 0 | 56 | 75.68 | 18 | 24.32 | 0 | 0 |

PCV: packed cell volume, MCV: mean corpuscular volume, MCH: mean corpuscular hemoglobin, MCHC: mean corpuscular hemoglobin concentration, WBC: white blood cells. The data of PP participants were shown at week 12 (visit 3).
