# Supplementary material for: Efficacy of UB0316, a multi-strain probiotic formulation in patients with type 2 diabetes mellitus: A double blind, randomized, placebo controlled study
Source: PLoS One. 2019 Nov 13;14(11):e0225168. doi: 10.1371/journal.pone.0225168 (PMC6853318; doi:10.1371/journal.pone.0225168)
Supplement: S8 Table — (DOCX) [file pone.0225168.s008.docx]

**S8 Table. Summary of vital signs of T2DM participants.**

|  | | **UB0316** | | | | | | **Placebo** | | | | | | **Total** | | | | | |
| --- | --- | --- | --- | --- | --- | --- | --- | --- | --- | --- | --- | --- | --- | --- | --- | --- | --- | --- | --- |
|  |  | **Normal** | | **Abnormal (CNS)** | | **Abnormal (CS)** | | **Normal** | | **Abnormal (CNS)** | | **Abnormal (CS)** | | **Normal** | | **Abnormal (CNS)** | | **Abnormal (CS)** | |
| **Test** | **Visit** | *n* | % | *n* | % | *n* | % | *n* | % | *n* | % | *n* | % | *n* | % | *n* | % | *n* | % |
| **Pulse** | **Baseline** | 40 | 100.00 | 0 | 0 | 0 | 0 | 38 | 97.44 | 1 | 2.56 | 0 | 0 | 78 | 98.73 | 1 | 1.27 | 0 | 0 |
|  | **Visit 3** | 37 | 100.00 | 0 | 0 | 0 | 0 | 37 | 100.00 | 0 | 0 | 0 | 0 | 74 | 100.00 | 0 | 0 | 0 | 0 |
| **Diastolic Blood Pressure** | **Baseline** | 38 | 95.00 | 2 | 5.00 | 0 | 0 | 35 | 89.74 | 4 | 10.26 | 0 | 0 | 73 | 92.41 | 6 | 7.59 | 0 | 0 |
|  | **Visit 3** | 35 | 94.59 | 2 | 5.41 | 0 | 0 | 37 | 100.00 | 0 | 0 | 0 | 0 | 73 | 97.29 | 2 | 2.71 | 0 | 0 |
| **Systolic Blood Pressure** | **Baseline** | 40 | 100.00 | 0 | 0 | 0 | 0 | 39 | 100.00 | 0 | 0 | 0 | 0 | 79 | 100.00 | 0 | 0 | 0 | 0 |
|  | **Visit 3** | 37 | 100.00 | 0 | 0 | 0 | 0 | 37 | 100.00 | 0 | 0 | 0 | 0 | 74 | 100.00 | 0 | 0 | 0 | 0 |
| **Temperature** | **Baseline** | 19 | 47.50 | 21 | 52.50 | 0 | 0 | 20 | 51.28 | 19 | 48.72 | 0 | 0 | 39 | 49.37 | 40 | 50.63 | 0 | 0 |
|  | **Visit 3** | 18 | 48.65 | 19 | 51.35 | 0 | 0 | 14 | 37.84 | 23 | 62.16 | 0 | 0 | 32 | 43.24 | 42 | 56.76 | 0 | 0 |
| **Respiratory Rate** | **Baseline** | 40 | 100.00 | 0 | 0 | 0 | 0 | 39 | 100.00 | 0 | 0 | 0 | 0 | 79 | 100.00 | 0 | 0 | 0 | 0 |
|  | **Visit 3** | 37 | 100.00 | 0 | 0 | 0 | 0 | 37 | 100.00 | 0 | 0 | 0 | 0 | 74 | 100.00 | 0 | 0 | 0 | 0 |

The data of PP participants were shown at visit 3 (week 12).
