# Supplementary material for: Efficacy of UB0316, a multi-strain probiotic formulation in patients with type 2 diabetes mellitus: A double blind, randomized, placebo controlled study
Source: PLoS One. 2019 Nov 13;14(11):e0225168. doi: 10.1371/journal.pone.0225168 (PMC6853318; doi:10.1371/journal.pone.0225168)
Supplement: S9 Table — (DOCX) [file pone.0225168.s009.docx]

**S9 Table. Summary of physical examination of T2DM participants.**

|  | | **UB0316** | | | | | | **Placebo** | | | | | | **Total** | | | | | |
| --- | --- | --- | --- | --- | --- | --- | --- | --- | --- | --- | --- | --- | --- | --- | --- | --- | --- | --- | --- |
|  |  | **Normal** | | **Abnormal (CNS)** | | **Abnormal (CS)** | | **Normal** | | **Abnormal (CNS)** | | **Abnormal (CS)** | | **Normal** | | **Abnormal (CNS)** | | **Abnormal (CS)** | |
| **Test** | **Visit** | *n* | % | *n* | % | *n* | % | *n* | % | *n* | % | *n* | % | *n* | % | *n* | % | *n* | % |
| **General Appearance** | **Baseline** | 38 | 95.00 | 2 | 5.00 | 0 | 0 | 33 | 84.62 | 6 | 15.38 | 0 | 0 | 71 | 89.87 | 8 | 10.13 | 0 | 0 |
|  | **Visit 3** | 36 | 97.30 | 1 | 2.70 | 0 | 0 | 35 | 94.60 | 2 | 5.40 | 0 | 0 | 71 | 95.95 | 3 | 4.05 | 0 | 0 |
| **Gastro Intestinal System** | **Baseline** | 38 | 95.00 | 2 | 5.00 | 0 | 0 | 38 | 97.44 | 1 | 2.56 | 0 | 0 | 76 | 96.20 | 3 | 3.80 | 0 | 0 |
|  | **Visit 3** | 36 | 97.30 | 1 | 2.70 | 0 | 0 | 37 | 100.00 | 0 | 0 | 0 | 0 | 73 | 98.65 | 1 | 1.35 | 0 | 0 |
| **Respiratory System** | **Baseline** | 40 | 100.00 | 0 | 0 | 0 | 0 | 39 | 100.00 | 0 | 0 | 0 | 0 | 79 | 100.00 | 0 | 0 | 0 | 0 |
|  | **Visit 3** | 37 | 100.00 | 0 | 0 | 0 | 0 | 36 | 97.30 | 1 | 2.70 | 0 | 0 | 73 | 98.65 | 1 | 1.35 | 0 | 0 |
| **Nervous System** | **Baseline** | 40 | 100.00 | 0 | 0 | 0 | 0 | 39 | 100.00 | 0 | 0 | 0 | 0 | 79 | 100.00 | 0 | 0 | 0 | 0 |
|  | **Visit 3** | 37 | 100.00 | 0 | 0 | 0 | 0 | 37 | 100.00 | 0 | 0 | 0 | 0 | 74 | 100.00 | 0 | 0 | 0 | 0 |
| **Genitourinary System** | **Baseline** | 39 | 97.50 | 1 | 2.50 | 0 | 0 | 37 | 94.87 | 2 | 5.13 | 0 | 0 | 76 | 96.20 | 3 | 3.80 | 0 | 0 |
|  | **Visit 3** | 35 | 94.60 | 2 | 5.40 | 0 | 0 | 36 | 97.30 | 1 | 2.70 | 0 | 0 | 71 | 95.95 | 3 | 4.05 | 0 | 0 |
| **Musculo-skeletal system** | **Baseline** | 29 | 72.50 | 11 | 27.50 | 0 | 0 | 29 | 74.36 | 10 | 25.64 | 0 | 0 | 58 | 73.42 | 21 | 26.58 | 0 | 0 |
|  | **Visit 3** | 28 | 75.67 | 9 | 24.33 | 0 | 0 | 32 | 86.50 | 5 | 13.50 | 0 | 0 | 60 | 81.08 | 14 | 18.91 | 0 | 0 |
| **Others** | **Baseline** | 39 | 97.50 | 1 | 2.50 | 0 | 0 | 39 | 100.00 | 0 | 0 | 0 | 0 | 78 | 98.73 | 1 | 1.27 | 0 | 0 |
|  | **Visit 3** | 37 | 100.00 | 0 | 0 | 0 | 0 | 37 | 100.00 | 0 | 0 | 0 | 0 | 74 | 100.00 | 0 | 0 | 0 | 0 |

The data of PP participants were shown at visit 3 (week 12).
