# Supplementary material for: Efficacy of UB0316, a multi-strain probiotic formulation in patients with type 2 diabetes mellitus: A double blind, randomized, placebo controlled study
Source: PLoS One. 2019 Nov 13;14(11):e0225168. doi: 10.1371/journal.pone.0225168 (PMC6853318; doi:10.1371/journal.pone.0225168)
Supplement: S1 Text — (PDF) [file pone.0225168.s011.pdf]

STUDY PROTOCOL

TITLE

A double blind, randomized, placebo controlled study to determine the safety and efficacy of **UB0316** as an adjuvant for 12 weeks in type 2 diabetes mellitus.

Protocol Number: IHS/UBL/03/16

Principal Investigator: Site I
Dr Anirudh Tripathi MD
Dr. Hemant Gupta MD Physician.
LIFE VEDA
Treatment and Research Centre
7/6, Century quarters,
Next to T.V. Tower, P.B. Road,
Worli, Mumbai – 400030

Co-Investigator: Site II
Dr. Satyavrat Nanal
Dr. Mahesh Talekar
Nanal Ayurvedic Clinic.
102, Anand bhuvan, Gorewadi,
Opposite MTNL Colony, Near Matunga Road Railway station,
Mahim west, Mumbai – 400016.

Sponsor:

Unique Biotech Ltd.
Address: Plot No. 2, Phase-II,
Alexandria Knowledge Park
Kolthur Village, Shameerpet Mandal,
Dist - Ranga Reddy, Hyderabad-500 078.

CRO:

Integrity Healthcare Services.
6/138, Poornima Building
Indulal Bhuva Marg
Wadala (West),
Mumbai- 400 031

Statement of Compliance

The trial will be conducted in compliance with the protocol, Good Clinical Practice (GCP) guidelines as per schedule Y of Drugs and Cosmetic act, 20th January 2005 *and Indian Council of Medical Research Ethical Guidelines for Biomedical Research India, 2006.* The principles enunciated in the Declaration of Helsinki (WMA General Assembly, Seoul 2008)

SIGNATURE PAGE

Study Title: A double blind, randomized, placebo controlled study to determine the safety and efficacy of UB0316 as an adjuvant for 12 weeks in type 2 diabetes mellitus.

Study Number: IHS/UBL/03/16

Prepared at Integrity Healthcare Services by:

Dr. Aasin Maurya, BAMS,<br>Medical Writer	20/06/16.<br>Date

SIGNATURE PAGE

Study Title: A double blind, randomized, placebo controlled study to determine the safety and efficacy of **UB0316** as an adjuvant for 12 weeks in type 2 diabetes mellitus.

Study Number: IHS/UBL/03/16

I have read this protocol and confirm that to the best of my knowledge it accurately describes the conduct of this study.

Approved at Unique Biotech Limited

Dr. M. Ratna Sudha

Unique Biotech Limited

Date

20/06/16

Dr. Aasin Maurya, BAMS
Medical Writer

Date

Integrity Healthcare Services Mumbai

p.p.

20/06/16

Mr. Gaurav Chauhan
Biostatistician

Date

Integrity Healthcare Services Mumbai

SIGNATURE PAGE

Study Title: A double blind, randomized, placebo controlled study to determine the safety and efficacy of **UB0316** as an adjuvant for 12 weeks in type 2 diabetes mellitus

Study Number: IHS/UBL/03/16

I have read this report and confirm that to the best of my knowledge it accurately describes the conduct of this study.

20/06/16

Date

Principal Investigator
Dr Anirudh Tripathi
MD Ayurvedic Medicine.

LIFE VEDA
Treatment and Research Centre
7/6, Century Quarters,
Next to T.V. Tower, P. B. Road,
Worli, Mumbai - 400 030.
Tel: 022 2497 8421, 30729461

Table of Contents

1. PROTOCOL SUMMARY 15

1.1 Study Design 19

1.2 Address And Designations 19

1.3 Authorised Person For Protocol Amendments 19

1.4 Clinical Investigator 20

1.5 Laboratory In-charge 20

1.6 Clinical Laboratory/ Institution 20

2 BACKGROUND INFORMATION AND SCIENTIFIC RATIONALE: 21

2.1 Rationale 27

2.1.1 Statement of Hypothesis: 29

2.1.2 Potential Risks and Benefits 29

1.1 Objective 29

3. STUDY DESIGN 34

3.1 Study Population 36

3.2 Inclusion/exclusion criteria 36

3.2.1 Inclusion criteria 36

3.2.2 Exclusion criteria 36

3.3. Enrollment/ Randomization/Masking Procedures 37

3.4. Subject Withdrawal And Dropouts 37

4. STUDY PROCEDURE AND EVALUATION 38

4.1 Screening 38

4.2 Study Procedure: 38

4.2.1 Enrollment/randomization/masking procedures: 41

4.3. Evaluations 41

4.3.1 Clinical Evaluations 42

4.3.2 Evaluation Of Questionnaire 43

4.3.4 Global Assessment (Crf) 45

4.3.5 Clinical Laboratory Evaluations 45

4.3.6 Evaluation 46

5. STUDY MEDICATION 47

5.1 Study Drug 47

5.2 Rescue Drug/Concomitant Drug 47

5.3 Prohibited Medication 47

6. STUDY SCHEDULE 48

7. DRUG DISPENSING 49

7.1 Study Product Acquisition 49

7.2 Formulation, Packaging and Labeling 49

7.3 Directions of Use ... 50

7.4 Product Storage and Stability ... 51

7.5 Accountability Procedures for the Study Intervention/Investigational Product(s) ... 51

7.6 Modification and Discontinuation of Study Intervention/Investigational Product for a Participant.. 51

7.7 Dose/Schedule Modifications for a Subject ... 52

7.8 Criteria for Discontinuation of Study Intervention/Product for Withdrawal of a Subject (or a Cohort) 52

8. ASSESSMENT OF SAFETY ... 52

8.1 Vitals/ Physical Examination: (Every Visit) ... 52

8.2 Additional examination ... 52

8.3 Systemic Examination: ... 52

8.3.1 Methods and Timing for assessing, recording, and analyzing the safety variables ... 52

8.3.2 Methods and Timing for Assessing, Recording, and Analyzing Safety Parameters ... 53

8.4 Assessment of Adverse Event ... 54

8.4.1 Definition of Adverse Event (AE) ... 54

8.4.2 Reporting Procedures ... 54

9. ASSESSMENT OF EFFICACY ... 56

10. CLINICAL DATA MANAGEMENT ... 56

10.1 Site Monitoring Plan ... 56

10.2 Source documents and Access to Source Data/Documents ... 56

10.3 Quality Control and Quality Assurance ... 57

11. ETHICS / PROTECTION OF HUMAN SUBJECTS ... 58

11.1 Declaration of Helsinki ... 58

11.2 Ethics Committee: ... 58

11.3 Informed Consent Process ... 58

11.4 Study Discontinuation ... 58

12. STATISTICAL CONSIDERATIONS ... 59

12.1 Introduction ... 59

12.2 Overview and study objective: ... 59

12.3 Study Population ... 59

12.4 Study Design ... 59

12.5 Study Outcome Measure ... 59

12.6 Study Hypothesis ... 60

12.7 Sample Size Consideration ... 60

12.7.1 Sample Size Justification and power calculation ... 60

12.8 Participant Enrollment and Follow – up ... 61

12.9 Planned interim analysis ... 61

12.10 Final Analysis Plan ... 61

13. DATA HANDLING AND RECORD KEEPING 62
13.1 Data Management Responsibilities 62
13.1.1 Data Management Roles and responsibilities of IHS- 62
13.1.2 Roles and Responsibilities of Sponsor- 63
13.1.3 Roles and Responsibilities of the Investigator 63
13.2 Data Capture Methods 63
13.2.1 Types of Data 63
13.2.2 Study Records Retention 64
13.3 Protocol Deviations 64
13.3.1 Protocol Deviation/ Violation Reporting- 64
14. REFERENCES: 66
15. SUPPLEMENTS/APPENDICES 69
15.1 **Appendix A: Schedule of Procedures and evaluations** 69
15.2. **Appendix B: QOL Questionnaire** 70
15.3 **Appendix C: Consent form** 72
15.4 **Appendix D: Investigators Name and Sites** 73
15.5 **Appendix E: Patient memory card** 74
15.6 **Appendix F: Statistical Analysis plan** 75

LIST OF ABBREVIATIONS

AE	Adverse Event/Adverse Experience
CRF	Case Report Form
CBC	Complete Blood Count
LDL	Low Density Lipoprotein
GCP	Good Clinical Practice
IB	Investigator’s Brochure
ICF	Informed Consent Form
ICH	International Conference on Harmonization
PIFS	Patient Information Sheet
IEC	Independent or Institutional Ethics Committee
PCOS	Polycystic Ovarian Syndrome
FBS	Fasting Blood Sugar
N	Number (typically refers to subjects)
PI	Principal Investigator
PPBS	Post-prandial blood-sugar
QA	Quality Assurance
QC	Quality Control
SAE	Serious Adverse Event/Serious Adverse Experience
ITT	Intention To Treat
SOP	Standard Operating Procedure
WHO	World Health Organization
DCGI	Drug Controller General of India
DCF	Data Clarification Form
Hb	Hemoglobin
IP	Investigational Product
SGPT	Serum glutamic-pyruvic transaminase
TRF	Test Request Form
T2DM	Type 2 diabetes mellitus
HbA1c	Glycated hemoglobin
HOMA	Homeostatic model assessment
CFU	Colony Forming Units
TG	Triglycerides
VLDL	Very Low Density Lipoproteins
HDL	High Density Lipoproteins
TC	Total Cholesterol
QOL	Quality Of Life
IDQOL	Impact of Diabetes on Quality Of Life
PPA	Per Protocol Analysis

<table>
<tr><td>NAME OF COMPANY:
Unique Biotech Ltd</td><td colspan="2">INDIVIDUAL STUDY SYNOPSIS
Page 1 of 3</td></tr>
<tr><td>NAME OF FINISHED PRODUCT:
UB0316</td><td colspan="2">NAME OF ACTIVE INGREDIENT(S):
Lactobacillus salivarius, Lactobacillus casei, Lactobacillus plantarum, Bifidobacterium breve, Lactobacillus acidophilus, Bacillus coagulans, Fructo-oligosaccharides</td></tr>
<tr><td colspan="3">Title of Study: A double blind, randomized, placebo controlled study to determine the safety and efficacy of UB0316 as an adjuvant for 12 weeks in type 2 diabetes mellitus.</td></tr>
<tr><td colspan="3">Investigators: Refer list of investigators in Appendix 13.4.D</td></tr>
<tr><td colspan="3">Study Sites: Refer list of study sites in Appendix 13.4.D - (2 SITES)</td></tr>
<tr><td colspan="3">Publication (Reference): None.</td></tr>
<tr><td colspan="2">Study duration: 12 Weeks</td><td>Phase of Development: II</td></tr>
<tr><td colspan="3">Objectives: To investigate the efficacy of probiotic adjuvant versus placebo on diabetes in a double blind, randomized controlled study</td></tr>
<tr><td colspan="3">Methodology: Patients satisfying all the inclusion and exclusion criteria will be enrolled in the study. A total of 70 patients will be enrolled. The patients will be randomized and will be given either placebo or study drugs for a duration of 12 weeks. They will be asked to visit on the Screening and Baseline day, Week 4, Week 8, and Week 12.</td></tr>
<tr><td colspan="3">Number of Patients: A total of 70 patients completing the study.</td></tr>
<tr><td colspan="3">Diagnosis and Main Criteria for Inclusion:
1. Male or female outpatient between the ages of 18- 65 years.
2. Subject states that he/she has type 2 diabetes (as evidenced by use of stable metformin monotherapy medication for at least 8 weeks prior to screening).
3. Subject’s BMI is > 23 kg/m^2 and < 32 kg/m^2
4. Subjects having an HbA1c level ≥ 7 % & ≤ 9%.
5. If on anti-hyperglycemic, anti-hypertensive, lipid-lowering, or thyroid medications or hormone therapy, has been on constant dosage for at least six months prior to screening visit.
6. Female, not currently pregnant or breast feeding and are using mechanical contraceptive devices such as Intra-uterine devices (IUD). (Barrier method of birth control; abstinence) prior to entry into study, during the period of study participation.
7. Ability to understand and the willingness to sign and date a written Informed Consent document at the screening visit before any protocol specific procedures are performed.

Exclusion Criteria:
1. Subject states that he/she has type 1 diabetes.
2. Subject states that he/she has history of diabetic ketoacidosis.
3. Subject uses anti-hyperglycemic medication other than metformin for glucose control.
4. Subject has fasting blood triglycerides > 400 mg/dL and/or LDL cholesterol > 190 mg/dL at screening.
5. Subject has an HbA1c level > 9.0% at screening.
6. Known hypersensitivity to any of the study drugs or constituents
7. Subjects suffering from severe systemic disease.
8. Subjects receiving any Ayurvedic, Homeopathic or Herbal drug continuously for one month; in last 30 days during screening visit.</td></tr>
</table>

**NAME OF COMPANY:**<br>Unique Biotech Ltd	**INDIVIDUAL STUDY SYNOPSIS**<br>**Page 2 of 3**
**NAME OF FINISHED PRODUCT:**<br>UB0316 Capsules	**NAME OF ACTIVE INGREDIENT(S):**<br>*Lactobacillus salivarius, Lactobacillus casei, Lactobacillus plantarum, Bifidobacterium breve, Lactobacillus acidophilus, Bacillus coagulans, Fructo-oligosaccharides*

Test Product, Dose, Mode of Administration, and Batch Numbers:
Each UB0316 (500 mg) capsule contains:

#	Ingredients	Quantity/per capsule
1	*Lactobacillus salivarius*	5 billion cfu
2	*Lactobacillus casei*	5 billion cfu
3	*Lactobacillus plantarum*	5 billion cfu
5	*Bifidobacterium breve*	5 billion cfu
6	*Lactobacillus acidophilus*	5 billion cfu
7	*Bacillus coagulans*	5 billion cfu
8	*Fructo-oligosaccharides (FOS)*	100 mg
9	*Maltodextrin*	370 mg

Dose: 2 UB0316 capsule/per orum/per day for 12 Weeks

Batch Number:

Duration of Treatment: 12 Weeks

Reference Therapy, Dose, Mode of Administration-

Placebo: 2 capsules/per orum/per day for 12 Weeks

IP: 2 UB0316 capsules/per orum/per day for 12 Weeks

Criteria for Evaluation:

Safety Parameters: Blood routine and Adverse event monitoring

Blood routine - Assessment **of** CBC, SGPT, Sr. Creatinine,

Efficacy parameters: Measurement of HBA1c, FBS, PPBS, Lipid Profile test, Serum Insulin, and HOMA-IR. Quality of Life questionnaire and physician's and subjects global assessment score.

Efficacy endpoints:

Primary Outcome Measures:

- The primary variable is change in hemoglobin A1c from baseline to week 12

Secondary Outcome Measures:

- Change in fasting blood glucose from baseline to week 12
- Change in fasting blood lipids (total cholesterol, LDL cholesterol, HDL cholesterol, VLDL cholesterol, triglycerides) from baseline to week 12
- Change in body weight from baseline to week 12
- Change in Quality of Life (QOL) from baseline to week 12

Safety endpoints:

1. Incidence of adverse events & Blood test (Baseline, week 6, Week 10 and week 12).

**NAME OF COMPANY:** Unique Biotech Ltd	**INDIVIDUAL STUDY SYNOPSIS** **Page 3 of 3**
**NAME OF FINISHED PRODUCT:** UBLAC Chewable tablets	**NAME OF ACTIVE INGREDIENT(S):** *Lactobacillus salivarius, Lactobacillus casei, Lactobacillus plantarum, Bifidobacterium breve, Lactobacillus acidophilus, Bacillus coagulans, Fructo-oligosaccharides*

Sample Size:

In justifying the sample size, we have made the assumptions which are given in below table for primary endpoint. The calculation to justify the sample size is based on the need to assure the study has a sufficient probability to detect the presence of proportion difference. Upto 92 patients are required to be screened and minimum 70 patients required evaluating the primary endpoint, which will provide 80% power to reject the null hypothesis (H_0 =Test – Placebo=0 verses H_a =Test – Placebo≠ 0) when the true overall mean difference is minimum 1.03 with a standard deviation of 1.5 at a significant level of 0.05.The sample size calculation was performed using SAS®9.4.

Statistical analysis:

The primary null hypothesis is that there is no difference between the UB0316 and the placebo with respect to the mean change from baseline in HB1AC at week 12. Reject this null hypothesis at a 2-sided alpha of 0.05 level will indicate the difference observed between the UB0316 and the placebo is statistically significant with respect to the mean change from baseline in HB1AC at week 12.

H_0: μ_T-μ_p=0

H_a: μ_T-μ_p≠0

Where T=UB0316
And P= Placebo

THE SAS SYSTEM
POWER PROCEDURE
Two sample test t test for mean difference

**Fixed scenario element**	
Distribution	Normal
Method	Exact
Mean difference	1.5
Standard dviation	1.5
Nominal power	0.80
Number of sides	2
Null difference	0
Alpha	.5
Group 1 weight	1
Group 2 weight	1

Actual power	N total
0.80	70

NAME OF COMPANY:<br>Unique Biotech Ltd	INDIVIDUAL STUDY SYNOPSIS<br>**Page 3 of 3**
**NAME OF FINISHED PRODUCT:**<br>UBLAC Chewable tablets	**NAME OF ACTIVE INGREDIENT(S):**<br>*Lactobacillus salivarius, Lactobacillus casei, Lactobacillus plantarum, Bifidobacterium breve, Lactobacillus acidophilus, Bacillus coagulans, Fructo-oligosaccharides*

So we will consider 92 number of patients to be screened (at least 70 patients completing the study) with a power is 0.80 (80% rejecting the null hypothesis when it is actually false i.e. correct decision); and this power will be sufficient.

SUMMARY OF RESULTS

Will be provided at the completion of the Trial.

Conclusions:

Will be provided at the completion of the Trial.

Date of Report: Will be provided at the completion of the Trial.

1. PROTOCOL SUMMARY

Description of Study Design:

A double-blind, randomized controlled study to investigate the efficacy of UB0316 capsule versus placebo in diabetes. Up to 92 subjects of either gender will randomized in the study assuming 20% dropout rate and 10% of screening failure final number of patients undergoing study will be 70 patients , the randomization will be done in 1:1 ratio and the block numbers will be generated by computer software.

Both males & females between 18-65 years of age with BMI between 23 kg/ m^2 and 32-kg/ m^2 and Subjects having an HbA1c level ≥ 7 % & ≤ 9% will be included in the study. Only Subjects he/she having type 2 diabetes (as evidenced by use of stable metformin monotherapy medication for at least 8 weeks prior to screening) will be considered for this study.

Subjects having Type I diabetes, history of diabetic ketoacidosis, fasting blood triglycerides > 400 mg/dL and/or LDL cholesterol > 190 mg/dL at screening, HbA1c level > 9.0% at screening will not be considered for the study purpose. Also subjects showing any hypersensitivity to any of the study drugs or constituents or having severe systemic disorders like AIDS, ulcerative colitis, Crohn's disease, coronary artery disease, pulmonary hypertension, neuropsychiatric illnesses, seizures will be excluded. . Subjects having herbal or alternative medicine 3 months prior to the date of enrollment in the study will be excluded from the study.

The primary variable is change in hemoglobin A1c from baseline to week 12 and changes in fasting blood glucose from baseline to week 12, Changes in fasting blood lipids (total cholesterol, LDL cholesterol, HDL cholesterol, VLDL cholesterol, triglycerides) from baseline to week 12, Changes in body weight from baseline to week 12, Changes in Quality of Life (QOL) from baseline to week 12 will be considered as secondary efficacy variables.

QOL (Quality of Life) questionnaire specific to the Impact of diabetes on Quality of Life (IDQOL / IDQOL) consisting of 34 items and eight domains: Physical Health (6 items), Physical endurance (6 items), General health (3 items), Treatment satisfaction (4 items), Symptom botherness (3 items), Financial worries(4 items), Emotional/mental health(5items) and Diet satisfaction (3 items) will be evaluated at baseline and week 4, week 8 and week 12. All items will be rated by the subject as per the designated responses and responses will be given a score of 1 - 5. Domain scores will be obtained by adding item scores, and the total score will be obtained by adding all domain scores. The scores shall be in a range of 1 - 159.

Patients will be treated with 2 UB0316 capsules after lunch. No concurrent medication for diabetes will be permitted during the study period. Patients who will require taking medicines for any other complaints during the course of study period shall be taken after consultation with the Investigator and record of it will be maintained in the Case Report Form.

Antidepressants, antipsychotic or appetite suppressant medicines, or tryptophan (to aid sleep), Weight altering medications including homeopathic, Ayurvedic, herbal (especially St. John's wart), Tibetan or Unani preparations, systemic steroids will be prohibited throughout the study.

CBC, SGPT, Sr. Creatinine, HBA1c, FBS, PPBS, Lipid Profile test, Serum Insulin and HOMA IR will be carried out in order to assess the safety of the patient at the time of Baseline & 3rd month. UPT assay shall be conducted to assess the pregnancy during screening period for females of childbearing age. All laboratory tests will be done by central pathology laboratory of Medilabs.

Assessment of CBC, SGPT, Sr. Creatinine, HBA1c, FBS, PPBS, Lipid Profile test, Serum Insulin AND HOMA-IR will be done at Baseline visit (Day 0) and in the Visit 3 (Week 12).

Trial will be carried out according to the protocol design & ICH-GCP Guidelines. Subjects will be given a MEMORY CARD which will show their follow up visits and schedule for their laboratory investigations along with Test Request Form for the type of tests to be done at each visit. Investigators at site, as per the ICH-GCP guidelines, will maintain source Documents which will include medical records, laboratory reports and subject diaries. The study site will maintain site Master File which will contain study related documents such as ICH-GCP Guidelines, Ethics Committee Approval Letter, Confidentiality Agreement, Clinical Trial Agreement, Study Protocol, Case Report Form, Investigator's Brochure, Patient Information Sheet, Consent Form (in different languages), Screening Form, Memory Card, Drug Accountability Record, Monitoring Log Record, Trial Supply Record document etc.

Table-1: Schematic representation of Study Design

**Type of Study**	**Sample Size**	Placebo	**Study Drug**	**Follow up visits**
Double Blind Study	70	35	35	Screening & Baseline,<br>Week 4, Week 8, and Week 12

Table 2: Flow chart diagram of study schedule

Screening Visit (Day 0)

1. Checking whether subject is complying Inclusion and Exclusion criteria from Study Synopsis
2. Assessment of Vitals, Systemic Examination, Family History, History of Disease,
3. Patient Informed Consent.
4. Enrollment of patient
5. Give Test Request Form.

70 subjects

Baseline Visit (Day 0 ±5)

6. CBC, SGPT, Sr. Creatinine, HBA1c, FBS, PPBS, Lipid Profile, Serum insulin and HOMO IR.
7. IDQOL
8. Dispense Study Medications for 4 weeks and call for 1st Follow up Visit after 4 weeks for assessment.
9. AE/SAE monitoring only for protocol procedure related events.

Visit 1 (Week 4 ±5 Day)

1. Assessment of Vitals, Assessment of QOL, Systemic Examination.
2. Monitoring AE/SAE,
3. Check Patients study drug compliance.
4. IDQOL
5. Dispense Study Medications for 4 weeks and check for compliance
6. Call for 2nd follow up Visit (week 8) for Assessment

Visit 2 (Week 8 ±5 Day)

1. Assessment of Vitals, Assessment of QOL, Systemic examination.
2. Systemic Examination. Provide TRF for week 12 blood investigations.
3. Monitor AE/SAE,
4. Patients study drug compliance
5. Dispense Study Medications for 4 weeks
6. Give Test Request Form for 3rd follow up Visit (Week 12)
7. Call for 3rd follow up Visit (Week 12) for Assessment

Visit 3 (Week 12 ±5 Day)

1. Assessment of Vitals and Lab investigations, Assessment of QOL,.
2. CBC, SGPT, Sr. Creatinine, HBA1c, FBS, PPBS, Lipid Profile, HOMO IR,Serum insulin.
3. Systemic Examination.
4. Note AE/SAE,
5. Check Patients study drug compliance
6. Physicians Global Assessment

KEY ROLES

1.1 STUDY DESIGN

A double blind, randomized, placebo controlled study to determine the safety and efficacy of **UB0316** as an adjuvant for 12 weeks in type 2 diabetes mellitus.

Protocol ID- **IHS/UBL/03/16**

Date: **- 20/06/2016**

1.2 ADDRESS AND DESIGNATIONS

Sponsor

Unique Biotech Ltd

Address: Plot No. 2, Phase-II,
Alexandria Knowledge Park
Kolthur Village,
Shameerpet Mandal,
Dist : Ranga Reddy,
Hyderabad- 500 078.

Medical Monitor-

Dr. Aasin Maurya, B.A.M.S

(Director Integrity Healthcare Services)

CRO Integrity Healthcare Services

301/302, Opulence Building,

6Th Road, Santacruz East,

Mumbai- 400 055.

Clinical Monitor-

Dr. Archana Trivedi, B.A.M.S.

1.3 AUTHORISED PERSON FOR PROTOCOL AMENDMENTS

Dr. Aasin Maurya, B.A.M.S.

Director at Integrity Healthcare Services

1.4 Clinical Investigator

Principal investigator: Dr Anirudh Tripathi $_{MD}$
LIFE VEDA Treatment and Research Centre.
7/6, Century quarters,
Next to T.V. Tower, P.B. Road, Worli,
Mumbai – 400030

Co-Investigator: Dr. Mahesh Talekar.
Co-Investigator: Dr. Satyavrat Nanal.
Nanal Ayurvedic Clinic.
102, Anand bhuvan, Gorewadi,
Opposite MTNL Colony, Near Matunga Road Railway station,
Mahim west, Mumbai – 400016.

1.5 Laboratory In-charge

Mr. Sanjay Upadhyay.
Medilab Diagnostics Center,
Shop no 9 and 10, Sunderdas compound Mumbai central,
Near Nair hospital casualty gate.
Mumbai - 400008

1.6 Clinical Laboratory/ Institution

The blood investigation, and biomarker assays will be conducted at:

Mr. Sanjay Upadhyay.
Medilab Diagnostics Center,
Shop no 9 and 10, Sunderdas compound Mumbai central,
Near Nair hospital casualty gate.
Mumbai - 400008

2 BACKGROUND INFORMATION AND SCIENTIFIC RATIONALE:

Diabetes mellitus (DM) is a group of metabolic disorders characterized by a chronic hyperglycemic condition resulting from defects in insulin secretion, insulin action or both.[1]

There are two main types of diabetes mellitus:

1) Diabetes, also called insulin dependent diabetes mellitus (IDDM), is caused by lack of insulin secretion by beta cells of the pancreas.
2) Diabetes, also called non-insulin dependent diabetes mellitus (NIDDM), is caused by decreased sensitivity of target tissues to insulin.[1]

Type 2 diabetes is a heterogeneous disorder caused by a combination of genetic factors related to impaired insulin secretion, insulin resistance and environmental factors such as obesity, over eating, lack of exercise, and stress as well as aging *(Kaku,2010*). It is typically a multifactorial disease involving multiple genes and environmental factors to varying extents *(Holt, 2004)*. Type 2 diabetes is the common form of idiopathic diabetes and is characterized by a lack of the need for insulin to prevent ketoacidosis.

Some people may be able to control their type 2 diabetes symptoms by losing weight, following a healthy diet, doing plenty of exercise, and monitoring their blood glucose levels. However, type 2 diabetes is typically a progressive disease - it gradually gets worse - and the patient will probably end up have to take insulin, usually in tablet form.

The two main pathological defects in type 2 diabetes are impaired insulin secretion through a dysfunction of the pancreatic β-cell, and impaired insulin action through insulin resistance *(Holt, 2004).*

Diabetes contributes to several kinds of bacterial infections like styes, boils, and folliculitis. People with diabetes are 40% more likely to suffer from glaucoma than people without diabetes. People with diabetes are also at 60% higher risk to develop cataracts. Other disorders caused by diabetes also include nerve damage, hypertension, the chances of having a stroke are 1.5 times higher than in people who don't have diabetes. [2]

People with diabetes have an increased risk of developing a number of serious health problems. Consistently high blood glucose levels can lead to serious diseases affecting the heart and blood vessels, eyes, kidneys, nerves and teeth. In addition, people with diabetes also have a higher risk of developing infections. In almost all high-income countries, diabetes is a leading cause of cardiovascular disease, blindness, kidney failure, and lower limb amputation. [3]

Etiology:

Type 2 diabetes is the predominant form of diabetes and accounts for at least 90% of all cases of diabetes mellitus *(Gonzalez et al., 2009)*. In developing countries, people aged 40 to 60 years (that is, working age) are affected most, compared with those older than 60 years in developed countries *(Shaw et al., 2010)*. This increase in type 2 diabetes is inextricably linked to changes towards a Western lifestyle (high diet with reduced physical activity) in developing countries and the rise in prevalence of overweight and obesity *(Chan et al., 2009; Colagiuri, 2010)*. The incidence of diabetes increases with age, with most cases being diagnosed after the age of 40 years. Type 2 diabetes is a heterogenous disorder caused by a combination of genetic factors related to impaired insulin secretion, insulin resistance and environmental factors such as obesity, over eating, lack of exercise, and stress as well as aging *(Kaku, 2010)*. It is typically a multifactorial disease involving multiple genes and environmental factors to varying extents *(Holt, 2004)*. Type 2 diabetes is the common form of idiopathic diabetes and is characterized by a lack of the need for insulin to prevent ketoacidosis.

Pathophysiology:

Type 2 diabetes mellitus is a heterogeneous syndrome characterized by abnormalities in carbohydrate and fat metabolism. The causes of type 2 diabetes are multifactorial and include both genetic and environmental elements that affect beta-cell function and tissue (muscle, liver, adipose tissue, pancreas) insulin sensitivity. A number of factors have been suggested as possibly linking insulin resistance and beta-cell dysfunction in the pathogenesis of type 2 diabetes. A majority of individuals suffering from type 2 diabetes are obese, with central visceral adiposity. Therefore, the adipose tissue should play a crucial role in the pathogenesis of type 2 diabetes. Although the predominant paradigm used to explain this link is the portal/visceral hypothesis giving a key role in elevated non-esterified fatty acid concentrations, two new emerging paradigms are the ectopic fat storage syndrome (deposition of triglycerides in muscle, liver and pancreatic cells) and the adipose tissue as endocrine organ hypothesis (secretion of various adipocytokins, i.e. leptin, TNF-α, resistin, adiponectin, implicated in insulin resistance and possibly beta-cell dysfunction). These two paradigms constitute the framework for the study of the interplay between insulin resistance and beta-cell dysfunction in type 2 diabetes as well as between our obesogenic environment and diabetes risk in the next decade.

Over the last decade, major advances have been made in our understanding of the pathophysiology and molecular biology of type 2 diabetes.

Major factors associated with type 2 diabetes are.

1) It is Characterised by defect in both insulin secretion and action.
2) It Depends on genetic make-up.
3) Environment.

4) It causes Disruption of working order between endocrine, pancreas, skeletal muscle, adipose tissue, gut, and CNS which leads to alteration of glucose homeostasis and type 2 diabetes.
5) It has Negative influence of intra-abdominal and visceral fat depot.
6) Proven Deleterious role of ectopic triglyceride causing lipotoxicity.
7) Adipose tissue secretes molecules like leptin, Tumour necrosis factor (TNF), adinopectin that may interfere with glucose metabolism and insulin sensitivity.

ROLE OF INSULIN RESISTANCE IN TYPE 2 DIABETES:-

Even if insulin exerts numerous different effects, so far insulin sensitivity has been considered mainly in the context of glucose metabolism, especially at the liver and muscle sites .The presence of insulin resistance in vivo can be evidenced during various dynamic tests such as an oral glucose tolerance test, an intravenous glucose tolerance test and a so-called euglycaemic hyperinsulinaemic clamp. Using the latter approach, it has been extensively demonstrated that insulin-mediated glucose disposal (essentially in the skeletal muscle) is largely reduced in patients with type 2 diabetes. Furthermore, the concomitant use of isotopes showed that hepatic glucose production is insufficiently inhibited by insulin, thus demonstrating the presence of both muscular and hepatic insulin resistance.
Genetic mutations associated with insulin resistance are rare and it seems unlikely that a single genetic alteration explains a large number of cases of insulin resistance among type 2 diabetic patients. Rather, it is more likely that a number of different genes may contribute, some of which may be obesity genes. Three commonly encountered factors that infl uence insulin sensitivity and are apparently not genetically determined are aging, exercise and dietary constituents. However, even if the effects of age, exercise and diet are considered, there is still a large between-subject variation in insulin sensitivity that has to be related to other factors. A major component of this residual variations may be related to obesity, but more importantly to differences in body fat distribution. A vast majority of type 2 diabetic patients are overweight, and obesity undoubtedly plays a major role in the development of the disease. While it is recognized that obesity is an important determinant of insulin sensitivity, body-fat distribution seems to be a critical aspect. Several groups have made a strong case that the intra-abdominally fat depot is the primary correlate of insulin sensitivity, while others have proposed that the central subcutaneous fat depot is the major factor determining a reduction in insulin sensitivity. Excess abdominal fat mass is associated with an increased release of NEFA that may trigger a reduction in insulin sensitivity at both the hepatic and the muscular levels. In the liver, this results in an increased glucose output (essentially due to enhanced gluconeogenesis), a decreased insulin extraction and an increased VLDL production while in the skeletal muscle this results in a reduction in glucose oxidation and glucose storage as glycogen (so-called Randle’s effect)
The ability of the adipocyte to function properly when engorged with lipid can lead to lipid accumulation in other tissues, reducing their ability to function and response normally. Liver steatosis is a common finding in obese subjects, especially in those with intra-abdominal fat depot, and non-alcoholic fatty liver disease is now considered as part of the metabolic

syndrome associated to insulin resistance. In addition, intramuscular triglyceride levels are increased in obese subjects, and a close relationship has been repeatedly reported between the degree of ectopic intramyocellular triglyceride depot and the severity of muscular insulin resistance. Interestingly, ectopic fat accumulation in insulin-sensitive tissues may be associated with insulin resistance independent of overall obesity. Finally, a hemodynamic hypothesis of insulin resistance has also been put forward. Reduced number of muscle capillaries and impaired insulin-induced vasodilatation (essentially in the postprandial state) may contribute to increase the distance and to alter the insulin diffusion process from the capillary to the muscular cells and thereby insulin action in obese patients with type 2 diabetes. This circulatory phenomenon may explain recent consistent findings showing that inhibition of the renin-angiotensin system (with either angiotensin converting enzyme inhibitors or AT1 receptor antagonists) is able to reduce the incidence of type 2 diabetes in hypertensive insulin-resistant patients.

ROLE OF INSULIN DEFICIENCY IN TYPE 2 DIABETES:-

Beta-cell deficit and beta-cell apoptosis are present in humans with type 2 diabetes. Once hyperglycaemia exists, beta-cell dysfunction is clearly present in subjects with type 2 diabetes. This change manifests in a number of different ways including decreases in the early insulin response to intravenous or oral glucose and a decline in the ability of glucose to potentiate the insulin response to non-glucose secretagogues. inefficient proinsulin processing to insulin and a reduction in the release of islet amyloid polypeptide (IAPP, also known as amylin) have been observed in established type 2 diabetes.

A backward extrapolation of findings made in the United Kingdom Prospective Diabetes Study (UKPDS) cohort from shortly after the clinical diagnosis of type 2 diabetes strongly suggests that beta-cell dysfunction commences years (at least 10 years) before hyperglycemia develops.

Three main mechanisms have been proposed to explain the B-cell deficiency observed in subjects prone to develop type 2 diabetes. First, a genetic defect may be present. Second, in utero malnutrition may lead to insufficient beta-cell development and later partial insulin secretory defect. And third, unfavourable metabolic environment may also play a deleterious role, especially increased glucose levels that may induce glucotoxicity and a chronic increase in NEFA levels that may induce lipotoxicity.

Finally, defects in insulin signalling pathways associated with insulin resistance in peripheral tissues have recently been found to disrupt insulin secretion by pancreatic beta cells, suggesting that insulin resistance in the beta cells may be, at least partly, responsible for the beta-cell dysfunction and the development of type 2 diabetes.

DYNAMIC INTERACTION BETWEEN INSULIN ACTION AND INSULIN SECRETION:-

Subjects with type 2 diabetes are characterized by both tissue insulin resistance and impaired insulin secretion. The development of diabetes requires the presence of these two fundamental defects, which disrupt the delicate balance by which insulin-target tissues communicate with the beta cells and vice versa. Type 2 diabetes occurs as a late phenomenon in obese subjects and is preceded by years of normal glucose tolerance or impaired glucose tolerance. The progression from IGT to diabetes occurs when the beta cell becomes unable to maintain its previously high rate of insulin secretion in response to glucose. Longitudinal studies confirmed that this evolution occurs along the natural history of obesity. During the
first years of obesity, the subjects are normoglycaemic but hyperinsulinaemic. Afterwards they become hyperglycaemic at a time when hyperinsulinaemia is not maintained anymore. To some extent, the natural history of the obese subjects developing type 2 diabetes after a prolonged phase of compensatory hyperinsulinism may be explained by the "overworked beta cell" hypothesis, the progressive deterioration of blood glucose control over the next 10 years can essentially be explained by a linear decrease of beta-cell insulin secretory capability.

ADIPOSE TISSUE AS AN ENDOCRINE ORGAN:-

The view of the adipocyte as a simply storage depot for fat is no longer tenable. Indeed, the role of adipose tissue as an endocrine organ capable of secreting a number of adipose- tissue-specific or enriched hormones, known as adipocytokines or adipokines, is gaining appreciation. Indeed, besides NEFA, adipocytes secrete various cytokines, among which leptin, TNF-α, resistin and adiponectin.

adiponectin, an abundant circulating protein synthesized solely in adipose tissue, appears to be a major modulator of insulin action. In contrast to other adipocytokines, adiponectin is characterized by lower (and not higher) circulating levels in presence of obesity. In addition, whereas leptin is more positively related to subcutaneous than to intra-abdominal fat, adiponectin is more strongly negatively related to intra-abdominal than to subcutaneous fat. Its levels are reduced in type 2 diabetes and a strong positive relationship between insulin sensitivity and adiponectin levels has been described in various populations. Thus, low adiponectin levels could contribute to peripheral insulin resistance in type 2 diabetes.

ROLE OF GUT MICROBIOTA IN TYPE 2 DIABETES:-

Muscle and adipose tissue resistance to insulin actions observed in T2D is triggered mainly by a complex combination of genetic predisposition, body composition, nutritional and environmental factors. Insulin receptor, glucose transporter and post-receptor perturbations are observed in T2D. Eventually, peripheral tissues exposed to chronic compensatory hyperinsulinemia become resistant to insulin. Studies have shown the intestinal microbiota is associated with the development of metabolic diseases, as obese and diabetic subjects present perturbations in the proportions of Firmicutes, Bacteroidetes and Proteobacterias. Mammals present sterile gastrointestinal tract at birth, Infants' intestinal microbiota is mainly formed by Bifidobacteria and Enterobacteria, and it changes progressively into a more complex pattern, observed in adults. Diet is pivotal for regulation of the intestinal microbiota, excess of nutrients

like saturated and polyunsaturated fatty acids or shortage of oligosaccharides and phytochemicals can modify the bacterial metabolic activity. High fat diets modify the intestinal microbiota, leading to increased intestinal permeability and susceptibility to microbial antigens, which ultimately correlates with the occurrence of metabolic endotoxemia and insulin resistance. Diabetic individuals have lower counts of Bifidobacterium and Faecalibacterium prausnitzii, both of them Gram + with anti-inflammatory properties. Despite the perturbations already observed in the intestinal microbiota of type 2 diabetic subjects, it is still necessary to elucidate whether the variations in the microbiota, intestinal barrier and metabolic endotoxemia are causes or consequences of diabetes. Interestingly, Mehta et al. [28] showed that acute inflammation induced by intravenous administration of LPS promotes metabolic endotoxemia and systemic insulin resistance, following modulation of specific adipose inflammatory and insulin signaling pathways. Concurrent with metabolic endotoxemia, translocation of live bacteria from the intestinal barrier into the blood appears to be related to the development of T2D. [4] Within ambient determinants, human overall gut bacteria have been identified as a crucial mediator of obesity and its consequences. Gut microbiota plays a crucial role in gastro-intestinal mucosa permeability and regulates the fermentation and absorption of dietary polyssacharides, which may explain its importance in the regulation of fat accumulation and the resultant development of obesity-related diseases. [5]

Clinical features:-

The following symptoms of diabetes are typical. However, some people with type 2 diabetes have symptoms so mild that they go unnoticed. [6]

- Urinating often
- Feeling very thirsty
- Feeling very hungry - even though you are eating
- Extreme fatigue
- Blurry vision
- Cuts/bruises that are slow to heal
- Tingling, pain, or numbness in the hands/feet

Cut-offs of obesity and abdominal obesity for Asian Indians vs. international criteria is as follows:

**Variable**	**Consensus guidelines for Asian Indians [a]**	**Prevalent International Criteria**
Generalized obesity (BMI cut-offs in kg/m2)	Normal: 18.0–22.9	Normal: 18.5–24.9 [b]
	Overweight: 23.0–24.9	Overweight: 25.0–29.9 [b]
	Obesity: >25	Obesity: >30 [b]
Abdominal obesity (Waist circumference cut-offs in cm)	Men: >90 [c]	Men: >102 [d]
	Women: >80 [c]	Women: >88 [d]
Notes: a From Consensus guidelines for Asian Indians [24]; b According to World Health Organization guidelines [25]; c Both as per Consensus Guidelines for Asian Indians [26] and International Diabetes Federation [26]; d According to Modified National Cholesterol Education Program, Adult Treatment Panel III guidelines [27]; Adapted from [23].		

EPIDEMIOLOGY:-

In 2013 it was estimated that over 382 million people throughout the world had diabetes. Diabetes is fast gaining the status of a potential epidemic in India with more than 62 million diabetic individuals currently diagnosed with the disease. In 2000, India (31.7 million) topped the world with the highest number of people with diabetes mellitus followed by China (20.8 million) with the United States (17.7 million) in second and third place respectively. The prevalence of diabetes is predicted to double globally from 171 million in 2000 to 366 million in 2030 with a maximum increase in India. It is predicted that by 2030 diabetes mellitus may afflict up to 79.4 million individuals in India [12]

India is one of the epicenters of the global diabetes mellitus pandemic. Rapid socioeconomic development and demographic changes, along with increased susceptibility for Indian individuals, have led to the explosive increase in the prevalence of diabetes mellitus in India over the past four decades. Type 2 diabetes mellitus in Asian Indian people is characterized by a young age of onset and occurrence at low levels of BMI. [13]
Obesity is one of the major risk factors for diabetes Despite having lower overweight and obesity rates, India has a higher prevalence of diabetes compared to western countries suggesting that diabetes may occur at a much lower body mass index (BMI) in Indians compared with Europeans Therefore, relatively lean Indian adults with a lower BMI may be at equal risk as those who are obese. Furthermore, Indians are genetically predisposed to the development of coronary artery disease due to dyslipidaemia and low levels of high density lipoproteins;[14]these determinants make Indians more prone to development of the complications of diabetes at an early age (20-40 years) compared with Caucasians (>50 years)

2.1 RATIONALE

Given the potential role of the intestinal microbiota in metabolic disorders, it is reasonable to hypothesize that restoration or supplementation of certain microbial populations may have a beneficial effect. In fact, In a study the hypothesis that intestinal microbiota in humans with type 2 diabetes is different from non-diabetic persons was tested and in this research it was demonstrated that the relative abundance of Firmicutes was significantly lower, while the proportion of Bacteroidetes and Proteobacteria was somewhat higher in diabetic persons compared to non-diabetic persons which is correlated to glucose tolerance. [15] In an obesity study, using mice models, Cani and coworkers [16] proposed a hypothesis connecting metabolic diseases with the presence of Gram-negative bacteria in the gut, The intestinal microbiota across the subjects with type 2 diabetes was relatively enriched with Gram-negative bacteria, belonging to the phyla *Bacteroidetes* and *Proteobacteria*. The main compounds of outer membranes in gram-negative bacteria are lipopolysaccharides (LPS), known as potent stimulators of inflammation, which can exhibit endotoxaemia Consequently, LPS will continue to be produced within the gut, which might trigger an inflammatory response and play a role in the development of diabetes. [17]

There have been some reports on the ant diabetic mechanism of LAB. In diabetes mellitus, administration of Lactobacillus casei to both non obese diabetic and noninsulin-dependent diabetic animals effectively inhibited the occurrence of diabetes by controlling the activity of the immune system *(Matsuzaki et al. 1997).* It was reported that the elevation of glucose intolerance and hyperglycaemia was significantly delayed by the feeding of Lactobacillus GG during the progression of streptozotocin-induced diabetes in rats *(Tabuchim et al. 2003).* Probiotic dahi containing Lactobacillus acidophilus and Lact. casei significantly delayed the progression of diabetes in the high-fat diet animals by slowing biochemical changes *(Yadav et al. 2007).* [18, 19, 20]

Also a study was conducted to evaluate the effect of the consumption of a symbiotic shake containing Lactobacillus acidophilus, Bifidobacterium bifidum and fructooligosaccharides on glycemia and cholesterol levels in elderly people. A randomized, double-blind, placebo-controlled study was conducted on twenty volunteers (ten for placebo group and ten for symbiotic group), aged 50 to 60 years. Results of the symbiotic group showed a non-significant reduction ($P > 0.05$) in total cholesterol and triglycerides, a significant increase ($P < 0.05$) in HDL cholesterol and a significant reduction ($P < 0.05$) in fasting glycemia. No significant changes were observed in the placebo group. Conclusion, the consumption of symbiotic shake resulted in a significant increase in HDL and a significant decrease of glycemia. [21].

In 2015 Marta A. Kasińska, Józef Drzewoski conducted a meta analysis of number of studies suggest that the use of probiotics may have a beneficial effect in patients with type 2 diabetes. PubMed, Embase, Cochrane Library, and Scopus databases were thoroughly reviewed up to January 2015 to search for randomized controlled trials (RCTs) that examined the effect of probiotics in patients with type 2 diabetes. The endpoints considered were: fasting plasma glucose (FPG), insulin concentration, insulin resistance, hemoglobin A1c (HbA1c), as well as the levels of total cholesterol, triglycerides, low-density and high-density lipoprotein cholesterols, and C-reactive protein (CRP). A total of 571 RCTs were initially identified, of which 8 trials with 438 individuals were selected for meta-analysis. The effects of probiotics were calculated for each parameter and the results showed a significant effect of probiotics on reducing HbA1c levels and HOMA-IR .Supplementation with probiotics did not have a significant effect on FPG, insulin, and CRP levels as well as the lipid profile.

Hari Om Yadav, Salini Jain, and P R. Sinha investigated the effects of low-fat (2.5%) dahi containing probiotic Lactobacillus acidophilus and Lactobacillus casei on progression of high fructose-induced type 2 diabetes in rats. Result showed that the probiotic dahi-supplemented diet significantly delayed the onset of glucose intolerance, hyperglycemia, hyperinsulinemia, dyslipidemia, and oxidative stress in high fructose-induced diabetic rats, indicating a lower risk of diabetes and its complications. [22,23]

Similarly, our probiotic formulation may influence the energy metabolism, lipolysis, anti-inflammatory effect and regulate the gut-microbiota to support the Diabetes treatment. This may probably establish overall relation of gut-health and diabetes. Therefore; the aim of this study is to investigate the UB0316 capsule supplementation for diabetes.

2.1.1 Statement of Hypothesis:

The primary null hypothesis is that there is no difference between the UB0316 and the placebo with respect to the mean change from baseline in HB1AC at week 12.

2.1.2 Potential Risks and Benefits

2.1.2.1 Potential Risks:

When ingested orally probiotics are generally considered safe and well tolerated. Most common adverse effects noted in previous studies include bloating and flatulence, however these are mild and subside with continued use other than that no known potential risks have been recorded for the formulation or the individual strains. [24]

2.1.2.2 Known Potential Benefits:

On entering the gastrointestinal tract, probiotics are unaffected by acid, bile salts and proteolytic enzymes. In the small bowel they multiply and live on the surface of epithelial cells. Their main beneficial effect is to act as a barrier to harmful organisms by adherence and production of substances that have an antibiotic effect, as well as stimulating immune processes in the host.[18] In the colon they have a major role of fermenting undigested carbohydrates and soluble dietary fiber, producing short-chain fatty acids. Probiotics may change the flora, affecting the fermentation process, so less gas are produced that may cause symptoms, or they may interfere with the growth or harmful effect of producing diarrhea, or they may stimulate the immune process to prevent some unidentified antigen response. Probiotics have been demonstrated to normalize or rebalance the GI microflora status quo, restoring gut epithelial function and the mucosal immunological barrier. [25]

1.1 Objective

Statement of Purpose- Primary objective of the study to determine the safety and efficacy of UB0316 as adjuvant for 12 weeks in type 2 diabetes mellitus.

General Purpose- To determine the safety and efficacy of UB0316 as adjuvant for 12 weeks in type 2 diabetes mellitus.

Names of Intervention:

UB0316 Capsule**:** In this clinical Trial we will study the effect of UB0316 Capsule (30 billion cfu) every day for 12 weeks.

Primary outcome – The primary variable is change in hemoglobin A1C from baseline to week 12.

Secondary outcomes –

- Change in fasting blood glucose from baseline to week 12
- Change in fasting blood lipids (total cholesterol, LDL cholesterol, HDL cholesterol, VLDL cholesterol, triglycerides) from baseline to week 12
- Change in body weight from baseline to week 12
- Change in Quality of Life (QOL) from baseline to week 12

Method of Assessing –

Primary Efficacy Variable:

Method of Assessing- The hemoglobin A1c test will be used as the method of assessing. Hemoglobin A1c, also glycated hemoglobin or A1c, is formed in the blood when glucose attaches to hemoglobin. [26] The more glucose in your blood, the more hemoglobin gets glycated. By measuring the percentage of A1C in the blood, you get an overview of your average blood glucose control for the past few months. The A1C test result is reported as a percentage. The higher the percentage, the higher a person's blood glucose levels have been. A normal A1C level is below 5.7 percent. [27]

Red cells live for 8 -12 weeks before they are replaced. By measuring the HbA1C it can tell you how high the blood glucose has been on average over the last 8-12 weeks. HB1Ac will be recorded based on the blood test results

Diagnosis*	A1C Level
Normal	below 5.7 percent
Pre – Diabetes	5.7 to 6.4 percent
Diabetes	6.5 percent or above

*Any test for diagnosis of diabetes requires confirmation with a second measurement unless there are clear symptoms of diabetes

Secondary Efficacy Variable:

Blood glucose monitoring will be used as method of assessing. Changes in FBG would be noted and compared from Baseline to week 12. Also changes in the lipid profile which includes LDL, VLDL, TC, Triglycerides would be checked from baseline to week 12 via routine lipid profiling. Change in body weight from baseline to week 12 and Quality of life will also be included in secondary efficacy parameters.

Quality of Life Questionnaire:

QOL (Quality of Life) questionnaire specific to the Impact of diabetes on Quality of Life (IDQOL / IDQOL) consisting of 34 items and eight domains: Physical Health (6 items), Physical endurance (6 items), General health (3 items), Treatment satisfaction (4 items), Symptom botherness (3 items), Financial worries (4 items), Emotional/mental health(5items) and Diet satisfaction (3.items) will be evaluated at baseline and week 4, week 8 and week 12. All items will be rated by the subject as per the designated responses and responses will be given a score of 1 - 5. Domain scores will be obtained by adding item scores, and the total score will be obtained by adding all domain scores. The scores shall be in a range of 1 - 159.

Diabetes: Quality of life questionnaire (IDQOL)

Sr. No	Questions	Response				
	**Role Limitation Due to Physical Health**	**1**	**2**	**3**	**4**	**5**
1.	How often do you miss your work because of your diabetes?	Always	Frequently	Often	Sometimes	Never
2.	A person with diabetes has the requirement of adhering to a schedule for eating and taking regular medication. How often does this affect your work?	Always	Frequently	Often	Sometimes	Never
3.	How often does diabetes affect your efficiency at work?	Always	Frequently	Often	Sometimes	Never
4.	How often do you find diabetes limiting your social life?	Always	Frequently	Often	Sometimes	Never
5.	To what extent do you avoid traveling (business tour, holiday, general outings) because of your diabetes?	A lot	Highly	Little	Very little	Not at all
6.	Compared to others of your age are your social activities (visiting friends/partying) limited because of your diabetes?	A lot	Highly	Little	Very little	Not at all
**Physical Endurance**						
1.	How often in last three months has your overall health problems limited the kind of vigorous activities you can do like lifting heavy bags/objects, running, skipping, jumping?	Always	Frequently	Often	Sometimes	Never
2.	How often in last three months has your overall health problems limited the kind of moderate activities you can do like moving a table, carrying groceries or utensils?	Always	Frequently	Often	Sometimes	Never
3.	How often in last three months has your overall health problems limited you from walking uphill or climbing 1-2 floors?	Always	Frequently	Often	Sometimes	Never
4.	How often in last three months has your overall health problems limited you from walking 1-2 km at a stretch?	Always	Frequently	Often	Sometimes	Never
5.	How often in last three months has your overall health problems limited you from bending, squatting, or turning?	Always	Frequently	Often	Sometimes	Never
6.	How often in last three months has your overall health problems limited you from eating, dressing, bathing, or using the toilet?	Always	Frequently	Often	Sometimes	Never
	**General Health?**					
1.	In general would you say your health is?	Poor	Fair	Good	Very Good	Excellent
2.	How well are you able to concentrate in everything like working, driving, reading etc.?	Not at all	Little	Moderate	very much	Extreme
3.	How many times in the past three months have you had fatigue/ felt very tired?	Always	Frequently	Often	Sometimes	Never
	**Treatment Satisfaction**					
1.	How satisfied are you with your current diabetes treatment?	Very dissatisfied	Moderately dissatisfied	Neither satisfied nor dissatisfied	Moderately Satisfied	Very Satisfied
2.	How satisfied are you with amount of time it takes to manage your diabetes?	Very dissatisfied	Moderately dissatisfied	Neither satisfied nor dissatisfied	Moderately Satisfied	Very Satisfied
3.	How satisfied are you with the amount of time you spend getting regular check-up`s (once in 3 months)?	Very dissatisfied	Moderately dissatisfied	Neither satisfied nor dissatisfied	Moderately Satisfied	Very Satisfied
4.	A person with diabetes needs to exercise for 35-45 min, 4 times a week. Keeping this in mind how satisfied are you with the time you spend exercising?	Very dissatisfied	Moderately dissatisfied	Neither satisfied nor dissatisfied	Moderately Satisfied	Very Satisfied

Sr. No	Questions	Response				
	**Symptom Botherness**	**1**	**2**	**3**	**4**	**5**
1.	How many times in the past three months have you felt excessive hunger?	Always	Frequently	Often	Sometimes	Never
2.	How many times in the past three months have you had frequent urination related to diabetes management?	Always	Frequently	Often	Sometimes	Never
3.	How many times in the past three months have you had frequent urination related to diabetes management?	Always	Frequently	Often	Sometime	Never
	**Emotional/Mental Health**					
1.	How satisfied are you with yourself?	Very dissatisfied	Moderately dissatisfied	Neither satisfied nor dissatisfied	Moderately Satisfied	Very Satisfied
2.	How satisfied are you with your personal relationships (family, friends, relatives and known tos)?	Very dissatisfied	Moderately dissatisfied	Neither satisfied nor dissatisfied	Moderately Satisfied	Very Satisfied
3.	How satisfied are you with the emotional support you get from your friends and family?	Very dissatisfied	Moderately dissatisfied	Neither satisfied nor dissatisfied	Moderately Satisfied	Very Satisfied
4.	How often are you discouraged by your health problems?	Always	Frequently	Often	Sometimes	Never
5.	All people want to fulfil certain roles and lead their lives in a purposeful manner. To what extent do you feel that you have been able to lead your life in the same way?	Not at all	Little	Moderate	very much	Extreme
	**Diet Satisfaction**					
1.	How often do you feel because of your diabetes a restriction in choosing your food when eating out?	Always	Frequently	Often	Sometimes	Never
2.	How often do you eat the food items that you shouldn't, in order to hide the fact that you are having diabetes.	Always	Frequently	Often	Sometimes	Never
3.	As you have diabetes, how much choice do you feel you have in eating your meals or snacks away from home e.g. if you go in a party and there is a buffet where there are also a lot of fried snacks and desserts would you be able to make enough choice?	No choice	very little	Little	Enough	A lot

Sr. No	Questions	Response			
	**Financial Worries**	**1**	**2**	**3**	**4**
1.	What do you think about the cost involved in your management of diabetes?	Very expensive	Little Expensive	Reasonable	Not at all expensive

		Response				
		**1**	**2**	**3**	**4**	**5**
2.	To what extent has your priority of expenditure shifted towards diabetes management?	A lot	Highly	Little	Very little	Not at all
3.	To what extent has your family budget got affected by the expenses related to the management of diabetes?	A lot	Highly	Little	Very little	Not at all
4.	To what extent has your diabetes limited your expenditure on other aspects of life (Movies, outings, parties etc.)?	A lot	Highly	Little	Very little	Not at all

*Nagpal j, Kumar A, Kakar S, Bhartia A. The development of 'Quality of Life Instrument for Indian Diabetes Patients (QOLID) : A Validation & Reliability study in Middle and Higher income Groups. May 2010; JAPI Vol 58. [28]

Evaluation

Domain	Score
Physical health	
Physical endurance	
General health	
Treatment satisfaction	
Symptom botherness	
Emotional/Mental Health	
Financial worries	
Diet satisfaction	
**Total Score**	

All items will be rated by the subject as per the designated responses and responses will be given a score of 1 - 5. Domain scores will be obtained by adding item scores, and the total score will be obtained by adding all domain scores. The scores shall be in a range of 1 - 159.

3. STUDY DESIGN

A double blind, randomized, placebo controlled study to determine the safety and efficacy of UB0316 as an adjuvant for 12 weeks in type 2 diabetes mellitus. Up to 92 subjects of either gender will randomized in the study assuming 20% dropout rate (cases expected to have 70 completed), the randomization will be done in 1:1 ratio and the block numbers will be generated by computer software.

Both males & females between 18-65 years of age with BMI between 23 kg/ m2 and 32-kg/ m2 and Subjects having an HbA1c level ≥ 7 % & ≤ 9% will be included in the study. Only Subjects he/she having type 2 diabetes (as evidenced by use of stable metformin monotherapy medication for at least 8 weeks prior to screening) will be considered for this study.
Subjects having Type I diabetes, history of diabetic ketoacidosis, fasting blood triglycerides > 400 mg/dL and/or LDL cholesterol > 190 mg/dL at screening, HbA1c level > 9.0% at screening will not be considered for the study purpose. Also subjects showing any hypersensitivity to any of the study drugs or constituents or having severe systemic disorders like AIDS, ulcerative colitis, Crohn's disease, coronary artery disease, pulmonary hypertension, neuropsychiatric illnesses, seizures will be excluded. . Subjects having herbal or alternative medicine 3 months prior to the date of enrollment in the study will be excluded from the study.
Subjects with history of depression, eating disorders such as anorexia nervosa, bulimia nervosa; drug and alcohol dependence will be excluded from the study. Pregnant or lactating females; females with history of PCOS* will not be included in the study. Subjects having herbal or alternative medicine 3 months prior to the date of enrollment in the study.

Primary efficacy variable will be the change in hemoglobin A1c from baseline to week 12. Changes in FBS, change in quality of life, change in lipid profile, and global assessment by investigator & patients self-assessments will be considered as secondary efficacy variables.

QOL (Quality of Life) questionnaire specific to the Impact of diabetes on Quality of Life (IDQOL / IDQOL) consisting of 34 items and eight domains: Physical Health (6 items), Physical endurance (6 items), General health (3 items), Treatment satisfaction (4 items), Symptom botherness (3 items), Financial worries(4 items), Emotional/mental health(5items) and Diet satisfaction (3 items) will be evaluated at baseline and week 4, week 8 and week 12. All items will be rated by the subject as per the designated responses and responses will be given a score of 1 - 5. Domain scores will be obtained by adding item scores, and the total score will be obtained by adding all domain scores. The scores shall be in a range of 1 - 159.

Patients were treated with 2 UB0316 Capsules after lunch. No concurrent medication for diabetes will be permitted during the study period. Patients who will required to take medicines for any other complaints during the course of study period shall be taken after consultation with the Investigator and record of it will be maintained in the Case Report Form.

Any other probiotics, Ayurvedic, herbal or Unani preparations, will be prohibited throughout the study.

CBC, SGPT, HBA1c, FBS, PPBS, Lipid Profile test, Serum insulin and HOMO IR will be carried out in order to assess the safety of the patient at the time of Baseline & 3rd month. UPT assay shall be conducted to assess the pregnancy during screening period for females of childbearing age. All laboratory tests will be done by central pathology laboratory of Medilabs

Assessment of CBC, SGPT, Sr. Creatinine, HBA1c, FBS, PPBS, Lipid Profile test, Serum Insulin, HOMA-IR will be done at Baseline visit (Day 0) and in the Visit 3 (Week 12).

Trial will be carried out according to the protocol design & ICH-GCP Guidelines.
Subjects will be given a MEMORY CARD which will show their follow up visits and schedule for their laboratory investigations along with Test Request Form for the type of tests to be done at each visit. Investigators at site, as per the ICH-GCP guidelines, will maintain source Documents which will include medical records, laboratory reports and subject diaries. The study site will maintain site Master File which will contain study related documents such as ICH-GCP Guidelines, Ethics Committee Approval Letter, Confidentiality Agreement, Clinical Trial Agreement, Study Protocol, Case Report Form, Investigator's Brochure, Patient Information Sheet, Consent Form (in different languages), Screening Form, Memory Card, Drug Accountability Record, Monitoring Log Record, Trial Supply Record document etc.

Table-1: Schematic representation of Study Design

Type of Study	Sample Size	Placebo	Study Drug	Follow up visits
Double Blind Study	70 Subjects	35 Patients	35Patients	Baseline, week 4, week 8, and week 12

3.1 Study Population

Total up to 92 patients of either sex of age range 18-65 years will be screened with other inclusion criteria and exclusion criteria, cases expected to have completed is 70 considering drop outs and screening failures from which 35 will receive the study drug and 35 patients will receive placebo.

3.2 Inclusion/exclusion criteria

3.2.1 Inclusion criteria

- Male or female outpatient between the ages of 18- 65 years.
- Subject states that he/she has type 2 diabetes (as evidenced by use of stable metformin monotherapy medication for at least 8 weeks prior to screening).
- Subject's BMI is > 23 kg/m^2 and < 32 kg/m^2
- Subject has an HbA1c level ≥ 7 % & ≤ 9%
- If on anti-hyperglycemic, anti-hypertensive, lipid-lowering, or thyroid medications or hormone therapy, has been on constant dosage for at least six months prior to screening visit.
- Female, not currently pregnant or breast feeding and are using mechanical contraceptive devices such as Intra-uterine devices (IUD). (Barrier method of birth control; abstinence) prior to entry into study, during the period of study participation.
- Ability to understand and the willingness to sign and date a written Informed Consent document at the screening visit before any protocol specific procedures are performed

3.2.2 Exclusion criteria

- Subject states that he/she has type 1 diabetes.
- Subject states that he/she has history of diabetic ketoacidosis.
- Subject uses anti-hyperglycemic medication other than metformin for glucose control.
- Subject has fasting blood triglycerides > 400 mg/dL and/or LDL cholesterol > 190 mg/dL at screening.
- Subject has an HbA1c level > 9.0% at screening.
- Known hypersensitivity to any of the study drugs or constituents
- Subjects suffering from severe systemic disease.
- Subjects receiving any Ayurvedic.
- Homeopathic or Herbal drug continuously for one month; in last 30 days during screening visit.

3.3. Enrollment/ Randomization/Masking Procedures

This is a double blind study, and therefore computer generated block randomization will be used. 70 patients are expected to complete the study. 35 patients will receive study drug and 35 patients will receive placebo. Random number will be allocated to patients in both arms as per the computer generated random numbers. Both groups will receive medications for 12 weeks. 10 bottles will be dispensed to both the sites during site initiation and subsequently as the study progresses again a set of 10 bottles will be dispensed when required at either site in timely fashion. The IP will be having a label depicting the randomization number as per the computer generated randomization sequence. Following is a short explanation of how the IP will be dispensed to both study sites:

1) Site 1 will receive 1-10 (randomly numbered IP) & Site 2 will receive 11-20 each a set of 10 bottles.
2) Based on the requirement next batch of set of 10 bottles of the IP will be dispatched to the respective site after it dispenses its initial quota of IP which will be checked and noted.
3) In an event of one of the sites unable to dispense the given set of IP, This set won't be supplied to the other site and would remain unused.

The serial numbers depicting the respective code on the IP will be placed in a sealed envelope and kept at both the sites in case of emergency. The serial numbers will be provided for enrollment of patients in relevant centers.

3.4. Subject Withdrawal And Dropouts

Subjects will be deemed as a withdrawn from the study on:

- Discretion of the investigator
- Repeated Protocol Deviations.
- Patients used any other drug than suggested concomitant medication during the trial duration other than the treatment given by the investigator.
- Serious Adverse events where continuation of the study possess serious risk to the patient.
- Patient has less than 80 % of compliance of the total drug that need to be consumed in the period between the follow ups
- Patient is without medication for more than 7 day.
- Worsening of the disease as per the patients' response and or investigator decides for standard therapy due to increase in infection.
- Patients' willingness to withdraw/ discontinue from the study or the study medication.

4. STUDY PROCEDURE AND EVALUATION

In this double blind, randomized placebo controlled study, 35 subjects will receive study drug and 35 subjects will receive placebo based on block randomization and the computer generated numbers. The UB0316 supplementation is intended to act as an adjuvant to Type II diabetes patients who are already on stable Metformin therapy. The Approximate time frame to complete the study enrollment is of 6 months to 1 year for 70 subjects. The expected duration of subject participation is 12 Weeks from the day of enrollment. Each subject will have to make four visits which will be recorded in CRF. No changes will be made to the dose scheduling and escalations. Stratifications are not applicable in the study.

4.1 Screening

Both males & females between 18-65 years of age with BMI between 23 kg/ m2 and 32-kg/ m2 and subjects having HbA1c level ≥ 7 % & ≤ 9% will be included in the study. Only Subjects he/she having type 2 diabetes (as evidenced by use of stable metformin monotherapy medication for at least 8 weeks prior to screening) will be considered for this study. Subjects having Type I diabetes, history of diabetic ketoacidosis, fasting blood triglycerides > 400 mg/dL and/or LDL cholesterol > 190 mg/dL at screening, HbA1c level > 9.0% at screening will not be considered for the study purpose. Also subjects showing any hypersensitivity to any of the study drugs or constituents or having severe systemic disorders like AIDS, ulcerative colitis, Crohn's disease, coronary artery disease, pulmonary hypertension, neuropsychiatric illnesses, seizures will be excluded. Subjects having herbal or alternative medicine 3 months prior to the date of enrollment in the study will be excluded from the study.

A total number of 70 patients will be completing the study, the randomization will be done on 1:1 ratio and the block numbers will be generated by computer. Patients will be randomized based on the qualification of inclusion and exclusion criteria.

During the screening period, patients qualifying all the criteria will be provided with the study drugs, Memory Card for follow-up visits and Lab investigations. The Lab investigations including CBC, SGPT, Sr. Creatinine, HBA1c, FBS, PPBS, Lipid Profile, Serum insulin and HOMA IR will be performed at the baseline and at the end of the study. The physician's global assessment; patient's global assessment and the Quality of Life questionnaire will be assessed at the baseline and at Week 4, Week 8 and week 12.

4.2 Study Procedure:

During the first/screening visit to the site, the physician will evaluate screening criteria to enroll patient in the study. After patient signed informed consent form, investigator performed detailed clinical, physical & systemic examination. Investigator recorded the disease history, present signs and symptoms and treatment taken in past. Blood sample was collected for CBC, SGPT, Sr. Creatinine, HBA1c, FBS, PPBS, Lipid Profile, Serum insulin and HOMA IR, if subject satisfied with the inclusion & exclusion criteria from the study synopsis. Based on these examination physician informed the patients about their eligibility for the study. In case patient was not eligible for the study, physician informed the patient and provided the patient with suggestive recommendations.

Screening:

During the first/screening visit to the site, the physician will evaluate screening criteria to enroll you in the study. If you satisfy the inclusion criteria, you will be enrolled in the study & consent will be explained followed by signing of the informed consent form. You will then be assigned the study drug based on the random enrolment in the study. After you have signed the informed consent form, investigator will perform detailed clinical, physical & systemic examination. You will be given a PATIENT MEMORY CARD consisting of the next Follow Up visit dates, dates for submission of blood sample for laboratory investigations and instructions for taking the study medication. The Study Doctor will record your disease history, present signs and symptoms and treatment taken in past. A Test Request Form will be given to you for collection of your blood for CBC, SGPT, Sr. Creatinine, HBA1c, FBS, PPBS, Lipid Profile, Serum Insulin and HOMA IR for the Baseline Visit. In case you are not eligible for the study, your study doctor will inform you and provide you with suggestive recommendations.

Baseline Visit (day 0)

During this visit, you will be asked to fill the impact of diabetes on QOL (Quality of Life) questionnaire consisting of 34 items and six domains: Physical Health (6 items), Physical endurance (6 items), General health (3 items), Treatment satisfaction (4 items), Symptom botherness (3 items), Financial worries (4 items), Emotional/mental health (5items) and Diet satisfaction (3 items. After this, the study medication will be dispensed to you for 30 days. Your next visit will be scheduled after 30 days. In the event of you unable to visit on scheduled date, you will have to visit for your follow up visit within (after/before) 4 days of your scheduled visit. The Study Doctor will assess your laboratory reports and re-evaluate your inclusion and exclusion criteria and decide whether or not you are eligible to take part in the study.

1st Follow up (Week 4)

On this visit, the study doctor will check your vitals and perform systemic examination. Your compliance in taking the study medication will be checked by counting the number of capsules returned. You will be asked to fill the Quality of Life questionnaire for diabetes and the score will be assessed. Any adverse events (known as unfavorable and unintended sign, symptom, or disease temporally associated or unassociated with study drug) or serious adverse event experienced by you during 4 weeks will be noted. You will then be dispensed a container containing study drug capsules for 4 weeks. You will have to bring the container along with you on the next visit & compliance of the study drug capsules will be assessed by counting the remaining capsules in the container. As instructed by your Study doctor, your next visit will be scheduled 4 weeks after visit 1. In the event of you unable to visit on scheduled date, you will have to come for your follow up within (after/before) 4 days of your scheduled visit.

2nd Follow up (Week 8)

On this visit, the study doctor will check your vitals and perform systemic examination. Your compliance in taking the study medication will be checked by counting the number of capsules returned. You will be asked to fill the Quality of Life questionnaire for diabetes and the score will be assessed. Any adverse events (known as unfavorable and unintended sign, symptom, or disease temporally associated or unassociated with study drug) or serious adverse event experienced by you during 4 weeks will be noted. You will then be dispensed a container containing study drug capsules for 4 weeks. You will have to bring the container along with you on the next visit & compliance of the study drug capsules will be assessed by counting the remaining capsules in the container. As instructed by your Study doctor, your next visit will be scheduled 4 weeks after visit 2. In the event of you unable to visit on scheduled date, you will have to come for your follow up within (after/before) 4 days of your scheduled visit. A Test Request Form will be given to you for collection of your blood for CBC, SGPT, Sr. Creatinine, HBA1c, FBS, PPBS, Lipid Profile,Serum Insulin and HOMA IR for the 3rd Follow up Visit.

3rd Follow Up (Week 12)

On this visit, the study doctor will check your vitals and perform systemic examination and assess the laboratory investigations. Your compliance in taking the study medication will be checked by counting the number of capsules returned. You will be asked to fill the Quality of Life questionnaire for diabetes and the score will be assessed. Any adverse events (known as unfavorable and unintended sign, symptom, or disease temporally associated or unassociated with study drug) or serious adverse event experienced by you during 4 weeks will be noted. The physician’s & patient’s global assessment of therapy will be done during this visit. It is a 5 point scale, where 1 indicates worst overall health condition and 5 indicates best overall health condition.

Recording Adverse Drug Reactions

Adverse Events (AE), Serious Adverse Events (SAE) and Adverse Drug Reactions (ADR) if any were recorded at every visit.

4.2.1 ENROLLMENT/RANDOMIZATION/MASKING PROCEDURES:

This is a double blind study, and therefore computer generated block randomization will be used. 70 patients are expected to complete the study. 35 patients will receive study drug and 35 patients will receive placebo. Random number will be allocated to patients in both arms as per the computer generated random numbers. Both groups will receive medications for 12 weeks. 10 bottles will be dispensed to both the sites during site initiation and subsequently as the study progresses again a set of 10 bottles will be dispensed in a plastic bag when required at either site in timesly fashion. The IP will be having a label depicting the randomization number per the computer generated randomization sequence. Following is a short explanation of how the IP will be dispensed:

1) Site 1 will receive 1-10 (randomly numbered IP) & Site 2 will receive 11-20 each a set of 10 bottles.
2) Based on the requirement next batch of set of 10 bottles of the IP will be dispatched to the respective site after it dispenses its initial quota of IP which will be checked and noted.
3) In an event of one of the sites unable to dispense the given set of IP, This set won’t be supplied to the other site and would remain unused.

The serial numbers depicting the respective code on the IP will be placed in a sealed envelope and kept at both the sites in case of emergency. The serial numbers will be provided for enrollment of patients in relevant centers.

4.3. EVALUATIONS

Assessment of CBC, SGPT, Sr. Creatinine, HBA1c, FBS, PPBS, Lipid Profile test, Serum Insulin and HOMA-IR will be done at Baseline visit (Day 0) and in the Visit 3 (Week 12).

Trial will be carried out according to the protocol design & ICH-GCP Guidelines.

Subjects will be given a MEMORY CARD which will show their follow up visits and schedule for their laboratory investigations along with Test Request Form for the type of tests to be done at each visit. The laboratory investigations will be performed on baseline and at 12 weeks.

4.3.1 Clinical Evaluations

After the patient has been told about the trial/ study, depending upon the patient's willingness an Informed consent form and consent will be obtained. The patients contact information will be recorded in the patient contact information sheet (PISF). The medical history of the patient will be recorded– OPD Papers or diaries of the patient will be obtained as source data verification document when the patient comes for screening visit, which mentions a detailed history about the disease and its reasons. On the same day the patient will be explained about the procedures in the trial and clinical evaluations will be carried out including assessment of stool test, followed by physical examination where vital signs like pulse rate, blood pressure and temperature will be taken and recorded in the Case Report Form (CRF).

Primary efficacy variable will be change in hemoglobin A1c and changes in fasting blood glucose, fasting blood lipids, change in body weight; and changes in quality of life, change in lipid profile, and global assessment by investigator & patients self-assessments will be considered as secondary efficacy variables.

Assessment of CBC, SGPT, Sr. Creatinine, HBA1c, FBS, PPBS, Lipid Profile test, Serum Insulin and HOMA-IR will be done at Baseline visit (Day 0) and in the Visit 3 (Week 12).

QOL (Quality of Life) questionnaire specific to the Impact of diabetes on Quality of Life (IDQOL / IDQOL) consisting of 34 items and six domains: Physical Health (6 items), Physical endurance (6 items), General health (3 items), Treatment satisfaction (4 items), Symptom botherness (3 items), Financial worries (4 items), Emotional/mental health(5items) and Diet satisfaction (3.items) will be evaluated at baseline and week 4, week 8 and week 12. All items will be rated by the subject as per the designated responses and responses will be given a score of 1 - 5. Domain scores will be obtained by adding item scores, and the total score will be obtained by adding all domain scores. The scores shall be in a range of 1 - 159.

CBC, SGPT, Sr. Creatinine, HBA1c, FBS, PPBS, Lipid Profile test, Serum insulin and HOMA IR will be carried out in order to assess the safety of the patient at the time of Baseline & 3rd month. UPT assay shall be conducted to assess the pregnancy during screening period for females of childbearing age. All laboratory tests will be done by central pathology laboratory of Medilabs.

In the event, if patient is dispensed with rescue medication; the same will be recorded in CRF during the follow-up visits. If In case of an AE/SAE the treatment assigned to the patient will be stop immediately and a systemic therapy will be given by the investigator depending upon the type of event. The Patient can withdraw from the study whenever he/she wants to discontinue or is not satisfied with the treatment.

4.3.2 EVALUATION OF QUESTIONNAIRE

QOL or IDQOL

QOL (Quality of Life) questionnaire specific to the Impact of diabetes on Quality of Life (IDQOL / IDQOL) consisting of 34 items and six domains: Physical Health (6 items), Physical endurance (6 items), General health (3 items), Treatment satisfaction (4 items), Symptom botherness (3 items), Financial worries(4 items), Emotional/mental health(5 items) and Diet satisfaction (3.items) will be evaluated at baseline and week 4, week 8 and week 12. All items will be rated by the subject as per the designated responses and responses will be given a score of 1 - 5. Domain scores will be obtained by adding item scores, and the total score will be obtained by adding all domain scores. The scores shall be in a range of 1 - 159.

Diabetes Questionnaire (IDQOL)

Sr. No	Questions	Response				
	**Role Limitation Due to Physical Health**	**1**	**2**	**3**	**4**	**5**
1.	How often do you miss your work because of your diabetes?	Always	Frequently	Often	Sometimes	Never
2.	A person with diabetes has the requirement of adhering to a schedule for eating and taking regular medication. How often does this affect your work?	Always	Frequently	Often	Sometimes	Never
3.	How often does diabetes affect your efficiency at work?	Always	Frequently	Often	Sometimes	Never
4.	How often do you find diabetes limiting your social life?	Always	Frequently	Often	Sometimes	Never
5.	To what extent do you avoid traveling (business tour, holiday, general outings) because of your diabetes?	A lot	Highly	Little	Very little	Not at all
6.	Compared to others of your age are your social activities (visiting friends/partying) limited because of your diabetes?	A lot	Highly	Little	Very little	Not at all
**Physical Endurance**						
1.	How often in last three months has your overall health problems limited the kind of vigorous activities you can do like lifting heavy bags/objects, running, skipping, jumping?	Always	Frequently	Often	Sometimes	Never
2.	How often in last three months has your overall health problems limited the kind of moderate activities you can do like moving a table, carrying groceries or utensils?	Always	Frequently	Often	Sometimes	Never
3.	How often in last three months has your overall health problems limited you from walking uphill or climbing 1-2 floors?	Always	Frequently	Often	Sometimes	Never
4.	How often in last three months has your overall health problems limited you from walking 1-2 km at a stretch?	Always	Frequently	Often	Sometimes	Never
5.	How often in last three months has your overall health problems limited you from bending, squatting, or turning?	Always	Frequently	Often	Sometimes	Never
6.	How often in last three months has your overall health problems limited you from eating, dressing, bathing, or using the toilet?	Always	Frequently	Often	Sometimes	Never
	**General Health?**					
1.	In general would you say your health is?	Poor	Fair	Good	Very Good	Excellent
2.	How well are you able to concentrate in everything like working, driving, reading etc.?	Not at all	Little	Moderate	very much	Extreme
3.	How many times in the past three months have you had fatigue/ felt very tired?	Always	Frequently	Often	Sometimes	Never
	**Treatment Satisfaction**					
1.	How satisfied are you with your current diabetes treatment?	Very dissatisfied	Moderately dissatisfied	Neither satisfied nor dissatisfied	Moderately Satisfied	Very Satisfied
2.	How satisfied are you with amount of time it takes to manage your diabetes?	Very dissatisfied	Moderately dissatisfied	Neither satisfied nor dissatisfied	Moderately Satisfied	Very Satisfied
3.	How satisfied are you with the amount of time you spend getting regular check-up's (once in 3 months)?	Very dissatisfied	Moderately dissatisfied	Neither satisfied nor dissatisfied	Moderately Satisfied	Very Satisfied
4.	A person with diabetes needs to exercise for 35-45 min, 4 times a week. Keeping this in mind how satisfied are you with the time you spend exercising?	Very dissatisfied	Moderately dissatisfied	Neither satisfied nor dissatisfied	Moderately Satisfied	Very Satisfied

*Nagpal j, Kumar A, Kakar S, Bhartia A. The development of 'Quality of Life Instrument for Indian Diabetes Patients (QOLID) : A Validation & Reliability study in Middle and Higher income Groups. May 2010; JAPI Vol 58.

Sr. No	Questions	Response				
	**Symptom Botherness**	**1**	**2**	**3**	**4**	**5**
1.	How many times in the past three months have you felt excessive hunger?	Always	Frequently	Often	Sometimes	Never
2.	How many times in the past three months have you had frequent urination related to diabetes management?	Always	Frequently	Often	Sometimes	Never
3.	How many times in the past three months have you had frequent urination related to diabetes management?	Always	Frequently	Often	Sometime	Never
	**Emotional/Mental Health**					
1.	How satisfied are you with yourself?	Very dissatisfied	Moderately dissatisfied	Neither satisfied nor dissatisfied	Moderately Satisfied	Very Satisfied
2.	How satisfied are you with your personal relationships (family, friends, relatives and known tos)?	Very dissatisfied	Moderately dissatisfied	Neither satisfied nor dissatisfied	Moderately Satisfied	Very Satisfied
3.	How satisfied are you with the emotional support you get from your friends and family?	Very dissatisfied	Moderately dissatisfied	Neither satisfied nor dissatisfied	Moderately Satisfied	Very Satisfied
4.	How often are you discouraged by your health problems?	Always	Frequently	Often	Sometimes	Never
5.	All people want to fulfil certain roles and lead their lives in a purposeful manner. To what extent do you feel that you have been able to lead your life in the same way?	Not at all	Little	Moderate	very much	Extreme
	**Diet Satisfaction**					
1.	How often do you feel because of your diabetes a restriction in choosing your food when eating out?	Always	Frequently	Often	Sometimes	Never
2.	How often do you eat the food items that you shouldn't, in order to hide the fact that you are having diabetes.	Always	Frequently	Often	Sometimes	Never
3.	As you have diabetes, how much choice do you feel you have in eating your meals or snacks away from home e.g. if you go in a party and there is a buffet where there are also a lot of fried snacks and desserts would you be able to make enough choice?	No choice	very little	Little	Enough	A lot

Sr. No	Questions	Response				
	**Financial Worries**	**1**	**2**	**3**	**4**	
1.	What do you think about the cost involved in your management of diabetes?	Very expensive	Little Expensive	Reasonable	Not at all expensive	
		**Response**				
		**1**	**2**	**3**	**4**	**5**
2.	To what extent has your priority of expenditure shifted towards diabetes management?	A lot	Highly	Little	Very little	Not at all
3.	To what extent has your family budget got affected by the expenses related to the management of diabetes?	A lot	Highly	Little	Very little	Not at all
4.	To what extent has your diabetes limited your expenditure on other aspects of life (Movies, outings, parties etc.)?	A lot	Highly	Little	Very little	Not at all

*Nagpal j, Kumar A, Kakar S, Bhartia A. The development of 'Quality of Life Instrument for Indian Diabetes Patients (QOLID) : A Validation & Reliability study in Middle and Higher income Groups. May 2010; JAPI Vol 58.

4.3.4 GLOBAL ASSESSMENT (CRF)

The Physician' and Patients' global assessment for overall improvement in the weight management and response to treatment will be recorded by the following Visits defined:

	Week 4	Week 8	Week 12
Physicians' Global Assessment	x	x	x
Patients' Global Assessment	x	x	x

4.3.5 CLINICAL LABORATORY EVALUATIONS

Visit Name	Blood Sample Submission Date
Screening Visit & Baseline Visit	XX
Follow Up Visit 1	-
Follow Up Visit 2	-
Follow Up Visit 3	XX

Table of Clinical Lab Investigations:/Normal Values

Hemoglobin	Males-13 – 18 gm%, Females-12 – 16.5gm%
RBC (total erythrocyte count)	4 – 5 million/cmm
WBC(total leucocyte count)	4,000 – 11,000/cmm
Neutrophils	40 – 75%
Eosinophils	1 – 6%
Basophils	0 – 1%
Lymphocytes	20 – 45%
Monocytes	2 – 10%
SGPT	0 – 40 IU/L
Serum creatinine	0.6 – 1.4 mg/dl
PCV	Males: 38.9-52.2, Females : 34.9-46 %
Platelets	150 – 400 x10^3/µL
MCH	27.0- 33.0 fL

HbA1c-Average Blood Glucose Reading :

%	Mmol/L	mg/DL	Inference
3-4	2–4	36-72	Extremely low to low blood sugar
4.5	5.5	99-100	Perfect
4.5-5	5-6	99-108	Normal Pre prandial
5.5-6	7-8	126-144	Normal Post prandial
6.5-7	9-10	162-180	Max Post prandial
6.5-7.5	9-11	162-198	High even for diabetics
7.5-9.5	11-15	198-270	Indicates poorly controlled bG

4.3.6 EVALUATION

All items will be rated by the subject as per the designated responses and responses will be given a score of 1 - 5. Domain scores will be obtained by adding item scores, and the total score will be obtained by adding all domain scores. The scores shall be in a range of 1 - 159.

**Domain**	**Score**
Physical health	
Physical endurance	
General health	
Treatment satisfaction	
Symptom botherness	
Emotional/Mental Health	
Financial worries	
Diet satisfaction	
**Total Score**	

5. STUDY MEDICATION

5.1 STUDY DRUG

2 UB0316 Capsules - 2 time a day, after meals for 12 Weeks.

5.2 RESCUE DRUG/CONCOMITANT DRUG

Human Insulin Rescue Medication
Open-label Human insulin, subcutaneous injection, as required as a rescue medication; dose determined per the investigator's discretion

Drug: Glimepiride Rescue Medication
Open-label glimepiride tablets, oral, as required as a rescue medication, dose determined per the investigator's discretion

5.3 PROHIBITED MEDICATION

- Probiotics usage other than study medication
- Herbal therapies

6. STUDY SCHEDULE

Sr. No	Tests & Assessments	Screening Baseline (Day 0 ± 5 days)	Visit 1 (Week 4 ± 5 days)	Visit 2 (Week 8 ± 5 days)	Visit 3 (Week 12 ±5 days)
**1.**	Screening	X	-	-	-
**2.**	Consent	X	-	-	-
**3.**	Enrollment	X	-	-	-
**4.**	Medical History	X	-	-	-
**5.**	Vitals	X	X	X	X
**6.**	Systemic examination	X	X	X	X
**7.**	TRF for Blood Test	X	-	X	-
**8.**	Primary efficacy Parameters	X	-	-	X
**9.**	Drug Dispensing	X	X	X	-
**10.**	Drug Compliance	-	X	X	X
**11.**	Blood Routine	X	-	-	X
**12.**	Patient Memory Card	X	X	X	X
**13.**	Quality of Life score	X	X	X	X
**14.**	Adverse event monitoring	X	X	X	X
**15.**	Physician's and Subject's Global Assessment	-	X	X	X

7. DRUG DISPENSING

Each UB0316 (500 mg) capsule contains:

#	Ingredients	Quantity/per capsule
1	*Lactobacillus salivarius*	5 billion cfu
2	*Lactobacillus casei*	5 billion cfu
3	*Lactobacillus plantarum*	5 billion cfu
5	*Bifidobacterium breve*	5 billion cfu
6	*Lactobacillus acidophilus*	5 billion cfu
7	*Bacillus coagulans*	5 billion cfu
8	*Fructo-oligosaccharides (FOS)*	100 mg
9	*Maltodextrin*	370 mg

Each PLACEBO (500 mg) capsule contains:

#	Ingredients	Quantity/per Capsule
1	FOS	100 mg
2	Colloidal silicon dioxide	5 mg
3	Magnesium stearate	5 mg
4	Maltodextrin	390 mg

7.1 STUDY PRODUCT ACQUISITION

The investigational products, which include the study drug & placebo, will be received from sponsor and the date of receipt of the study medication will mentioned in the project master file. The unused or returned medication will be maintained in project master file. The batch number, manufacturing dates and expiry dates will be maintained for IPs in the project master file. Dispatch to various study sites and the receipt of the remaining, unused or tempered IPs will be recorded in the drug dispensing log of individual sites.

7.2 FORMULATION, PACKAGING AND LABELING

The UB0316 capsule will be supplied in HDPE container with silica gel bag to avoid moisture. Each container will contain 70 capsules of each study drug and placebo. (As the subject has to take IP in the form of capsules twice a day, 70 capsules are calculated in the following manner, 4 weeks ± 5 days, A quota of 60 capsules for outlined duration and 10 capsules for deviation of 5 days as per the protocol)

Table 3: Composition

Each UB0316 (500 mg) capsule contains:

#	Ingredients	Quantity/per capsule
1	*Lactobacillus salivarius*	5 billion cfu
2	*Lactobacillus casei*	5 billion cfu
3	*Lactobacillus plantarum*	5 billion cfu
5	*Bifidobacterium breve*	5 billion cfu
6	*Lactobacillus acidophilus*	5 billion cfu
7	*Bacillus coagulans*	5 billion cfu
8	*Fructo-oligosaccharides (FOS)*	100 mg
9	*Maltodextrin*	370 mg

Table 4: Composition of PLACEBO

Each PLACEBO (500mg) Capsule contains:

#	Ingredients	Quantity/per Capsule
1	FOS	100 mg
2	Colloidal silicon dioxide	5 mg
3	Magnesium stearate	5 mg
4	Maltodextrin	390 mg

7.3 Directions of Use

2 capsules - 1 time a day after meal.

7.4 Product Storage and Stability

The UB0316 capsules will be supplied in HDPE Container. Each Container will contain 70 capsules of the study drug and placebo. Containers will be stored at the temperature of 2-8◦ C in cool dry place away from direct sunlight. Control medications, rescue medication or concomitant medications will be stored as per the manufacturers' instruction. The drug accountability log will describe the storage temperature.

7.5 Accountability Procedures for the Study Intervention/Investigational Product(s)

The study Intervention will be distributed having the center number, manufactured date, expiry date, temperature under which it has to be stored, batch number, ingredients, composition, the dosage form, route of administration, what are the risk and benefits, in how much amount it has to be taken.

The IP will be shipped through a courier by the sponsors to the site/ center. A set of 10 containers each containing 70 capsules of the study drug (Dosage of 4 weeks ± 5 days). The IPs will be dispensed to the patients/ subjects by the investigator containing a unique identification number mentioned by the monitor as per randomization chart. A drug accountability log will be prepared to keep the tracking records of the IP's dispensed and received.

.

	Screening & Baseline (Day 0)	Visit1 (Week 4)	Visit 2 (Week 8)	Visit 3 (Week 8)
Number of Capsules dispensed	x	x	x	-
Number of Capsules returned	-	x	x	x

The IP consists of 70 numbers of the capsules of Placebo & study Drug. The IP will be dispensed on the screening day, and on subsequent visits depending on usage. After the patients have made the last follow up visit, the IP will be procured and will be maintained at the site/ center.

7.6 Modification and Discontinuation of Study Intervention/Investigational Product for a Participant

In case of occurrence of Serious Adverse Event, PI should intimate the CRO within 24 hours and to local Ethics committee within 7 days .The CRO will keep the other Sites involved in the Study informed about the development of Serious ADRs. For all adverse events, the attending Investigator must pursue and obtain information adequate to determine the Outcome of the SAE/AE and must opine whether the adverse reaction could have occurred due to treatment administration.

7.7 Dose/Schedule Modifications for a Subject

There will be no modification made to the drug and in the dose given to the patients. The Dose of the drug would be stopped immediately after the patient experiences the adverse event. He will be then switched to standard treatment depending on severity of the condition (antiviral therapy may or may not be required) or then in certain other cases the patient will be treated by the investigator (Only if he feels that the patient does not matches with the inclusion criteria as mentioned in the protocol). The Investigator will have to inform the CRO regarding the adverse event. If serious adverse event occurs, the subject will be hospitalized until complete recovery of the subject is done and will be excluded from the study.

7.8 Criteria for Discontinuation of Study Intervention/Product for Withdrawal of a Subject (or a Cohort)

The subject will be discontinued from the study if

1) There is abnormal laboratory value in the safety assessment report.
2) Subject experiences AE /SAE
3) On the Patients willingness to discontinue.
4) Increase in the complications of Type 2 Diabetes.
5) Violation of Protocol and Development of exclusion criteria.

8. ASSESSMENT OF SAFETY

8.1 Vitals/ Physical Examination: (Every Visit)

Pulse Rate, Respiratory Rate, Temperature, Systolic & Diastolic Blood Pressure.

8.2 Additional examination

Blood Test

8.3 Systemic Examination:

8.3.1 Methods and Timing for assessing, recording, and analyzing the safety variables

8.3.1.1 Measurement Schedule: (Screening, Day 0, Week 4, Week 8, Week 12)

- Vitals
- Systemic Examination
- Central Nervous System
- Gastrointestinal System
- Respiratory System
- Cardiovascular System
- Genitourinary System
- Endocrine System
- Others

8.3.1.2 The following lab tests are carried out in order to assess the safety of the patient.

a. Blood Routine

b. Adverse Events

The normal values of safety assessment are mentioned in the point number 4.4.

8.3.2 Methods and Timing for Assessing, Recording, and Analyzing Safety Parameters

Sr No	Patient ID	Within first 3 Days of Baseline	Week 12
		Blood Routine	Blood Routine

8.4 Assessment of Adverse Event

8.4.1 Definition of Adverse Event (AE)-

(As per ICH-GCP E6 guidelines) Any untoward medical occurrence in a patient or clinical investigation subject administered a pharmaceutical product and which does not necessarily have a causal relationship with this treatment. An adverse event (AE) can therefore be any unfavorable and unintended sign (including an abnormal laboratory finding), symptom, or disease temporally associated with the use of a medicinal (investigational) product, whether or not related to the medicinal (investigational) product (see the ICH Guideline for Clinical Safety Data Management: Definitions and Standards for Expedited Reporting).

Serious Adverse Event

Definition of Serious Adverse Events or Serious Adverse Drug Reaction as per ICH-GCP - Any untoward medical occurrence that at any dose:

1) Results in Death,

2) Is Life Threatening

3) Requires Inpatient hospitalization or prolongation of existing hospitalization

4) Results in persistent or significant disability/ incapacity, or

5) Is a congenital anomaly/ birth defect.

8.4.2 Reporting Procedures

8.4.2.1 Serious Adverse Event Detection and Reporting

In case of SAE's, the Investigator should report to the sponsor immediately followed by promptly written detailed report with a SAE form. The immediate and follow up reports should identify subjects by a unique code numbers assigned, rather than its name and personal identification numbers or addresses. The Investigator should also comply with the regulatory requirements, related to the reporting of unexpected serious adverse drug reactions to the regulatory authority (ies) and the IEC. The investigator should promptly report in writing as well as make a telephonic call to IEC and sponsor.

8.4.2.2 Reporting of Pregnancy

Pregnant women are not included into the trials. In case a patient gets conception during the trial, she will be withdrawn from the study and will continue to receive the standard care treatment. If the subject has already used the IP and continues to come for follow up visits, she will be considered in ITT analysis.

8.4.2.3 PROCEDURES TO BE FOLLOWED IN THE EVENT OF ABNORMAL LABORATORY TEST VALUES OR ABNORMAL CLINICAL FINDINGS

Abnormal laboratory data determined will be safety investigations like CBC, FBS, PPBS, Lipid profile, HB1Ac, Serum insulin, and HOMO IR. In case of Abnormal Laboratory Clinical Finding, A data clarification form would be sent to the Investigator. Investigator will diagnose the disease condition of the patient. If the patient is diagnosed with serious illness, the patient will be discontinuing from the trial and can still take the necessary treatment from his/her investigator.

8.4.2.4 TYPE AND DURATION OF THE FOLLOW-UP OF SUBJECTS AFTER ADVERSE EVENTS

In case if any patient encounters an adverse events then the patient will be switched from the study drug/ medication to the standard treatment at the discretion of the study doctor. The standard treatment will generally be antibiotics or painkillers. Antivirals are only effective if administered during prodromal stage or within 1 day of onset of lesions, and therefore may not be used in most cases. The patient will have to continue to come for the follow up visit as mentioned in the protocol, there will be no change of schedule in the follow up visits. The investigator will record and report the adverse event in the CRF and in any other document as provided by the sponsor as per Schedule Y guidelines. The investigator will inform the sponsor about the adverse event and about the treatment provided to the patient along with study drug.

8.4.2.5 HALTING RULES

The Trial will be halted if significant number of adverse events, frequency of adverse events is increased, the no of particular type of SAE, Severe AEs/reactions are noted from all the sites. It will be halted with the Approval of Ethics committee. Sponsor And CRO cannot halt the Trial.

9. ASSESSMENT OF EFFICACY

Efficacy endpoints:

Primary Outcome Measures:

1. The primary variable is change in hemoglobin A1c from baseline to week 12

Secondary Outcome Measures:

1. Change in fasting blood glucose from baseline to week 12
2. Change in fasting blood lipids (total cholesterol, LDL cholesterol, HDL cholesterol, VLDL cholesterol, triglycerides) from baseline to week 12
3. Change in body weight from baseline to week 12
4. Change in Quality of Life (QOL) from baseline to week 12

Safety endpoints:

Incidence of adverse events & Blood Test.

10. CLINICAL DATA MANAGEMENT

10.1 Site Monitoring Plan

Study monitoring will be done at each site within 2 Days minimum or maximum of previous follow up. Discrepancies in the study related documentations will be noted and informed to study coordinator/PI. In case of serious discrepancies i.e. other than typological error, the manual Data Clarification Form (DCF) will be generated. The site shall complete the DCF within 3 working from the date of generation. The completed DCF will be archived in SMF as well as in Project Master File (PMF). After, data clarification, finished data will be entered in database format for analysis and manual double data check will be done. After this, final data will be locked and send for Statistical Analysis.

10.2 Source documents and Access to Source Data/Documents

Definition of Source Data/Documents

Source Data- All Information in original records and certified copies of original records of clinical findings, observations, or other activities in a clinical trial necessary for the reconstruction and evaluation of the trial. Source data are contained in source documents (original records or certified copies).

Source Documents- Original documents, data, and records (e.g., hospital records, clinical and office charts, laboratory notes, memoranda, subjects' diaries or evaluation checklists, pharmacy dispensing records, recorded data from automated instruments, copies or transcriptions certified after verification as being accurate copies, subject files, and records kept at the pharmacy, at the laboratories and at medico-technical departments involved in the clinical trial).

The investigator should ensure that the source data/ document will be provided/ collected from the patient. The source document is the document that gives you a brief details about the history of the patient, It can be the OPD paper that the patient maintains which he/ she receives it from the hospital, or it can be the diary which is maintained by the patient whenever he visits the hospital or the institution or the clinic for the treatment of any kind diseases or whenever he is not well. A copy of the source document is to be collected by the investigator or the coordinator

(if any) for the evidence purpose, so that, in case of an adverse event or serious adverse event the records of the patient can be accessed to check for any abnormal values in the laboratory values or in any other surgical or diagnostic intervention.

10.3 Quality Control and Quality Assurance

Each site will have standard operating procedures (SOPs) for quality management. Data will be evaluated for compliance with protocol and accuracy in relation to source documents. The study will be conducted in accordance with procedures identified in the protocol. The site activity delegation log will be completed and the study related documents will be reviewed and the schedule for reviews will be specified in monitoring logs of the site.

Each study site will be initiated with training and ascertain the activity dedicated to the study personnel. The training will be re-conducted in case of site requires and in case of any study related or protocol related process change as per the amended protocol.

11. ETHICS / PROTECTION OF HUMAN SUBJECTS

11.1 DECLARATION OF HELSINKI

The trial will be conducted in accordance to Declaration of Helsinki for the Protection of Human Subjects during the conduct

11.2 ETHICS COMMITTEE:

The study will be conducted as institute run out patient department and the ethical approval will be taken from the Institutes'' Ethics committee. Study conduct at the private clinics will be approved by an Independent Ethics Committee as per the guidelines of ICMR.

11.3 INFORMED CONSENT PROCESS

Before enrolling the patient to the trial, investigator would ask the patient whether he is interested to participate in the Trial. The Investigator would then explain to the patient the study procedure, the possible risk and benefits of the study, what laboratory and safety parameters will be conducted, whether there is requirement of interventional procedure. The patient would be given time to think and ask his family or other members. He would also be told that he has right to withdraw himself from the study at any point of time and his rights will be safeguarded. He will also be given regular treatment in case he does not comply with the protocol. If the patient agrees to participate in the trial, consent will be explained to him and/or his legally accepted representative.

Subject Confidentiality

The Patient will be assigned a unique identification Number. Names address and contact details will not be disclosed to others except the investigator, ethics committee, CRO and The Sponsor. Biological samples taken from the patients would be preserved only during the Study. After completion of the study, they will be destroyed

The study protocol, documentation, data and all other information generated will be held in strict confidence. No information concerning the study or the data will be released to any unauthorized third party, without prior written approval of the sponsor. The study monitor or other authorized representatives of the sponsor may inspect all documents and records required to be maintained by the Investigator, including but not limited to, medical records (office, clinic or hospital) and pharmacy records for the subjects in this study. The clinical study site will permit access to such records.

11.4 STUDY DISCONTINUATION

The subjects who fulfill the inclusion and exclusion criteria as mentioned in the protocol are taken into the study/ trial. If in case of AE/ SAE the patients will be withdrawn from the study by the investigator and will be given a standard treatment. In case of SAE the IRB/ IEC has to be reported in writing within 14 working days and it should be reported in writing to the sponsor within 24 hours. SAE should be notified in writing to the IRB/IEC within 7 working days. If a subject is non-compliant then he will be given a standard treatment and if it's a lost to

follow up then the subject will be considered as a dropout from the study, and will be given a standard treatment.

12. STATISTICAL CONSIDERATIONS

12.1 INTRODUCTION

UB0316 Study capsules have to be taken twice a day. The effect size of the two groups will be compared and the data will be evaluated with SAS®9.4 software.

12.2 OVERVIEW AND STUDY OBJECTIVE:

Primary objective of the study is to determine the effect of UB0316 Capsule on type 2 diabetes

12.3 STUDY POPULATION

A total of upto 92 patients including male and female participants between 18-65 years of age will be included in the trial based upon the study selection criteria.

12.4 STUDY DESIGN

The study is a double blind, randomized controlled study to investigate the efficacy of UB0316 versus placebo on type 2 diabetes.

12.5 STUDY OUTCOME MEASURE

PRO consists of Quality of Life (QoL) Questionnaire. The PRO recording corrections will be instructed if require. The outcome of the blood test will be noted and briefed in case of any abnormal values. Patients' physical and vital examination, medical history will be recorded. Patients' Quality of Life assessment will be done on 5 point scale. It consists of total 34 questions, divided into 8 categories: Physical Health (6 items), Physical endurance (6 items), General health (3 items), Treatment satisfaction (4 items), Symptom botherness (3 items), Financial worries (4 items), Emotional/mental health (5items) and Diet satisfaction (3 items). Hb1AC will be recorded at baseline and week 12 visit. Blood test results will be assessed at baseline and week 12 visit for Hb1AC, Fasting blood sugar; PPBS, lipid profile, SGPT, Sr. Creatinine, serum insulin and HOMA IR. Physician's and Subject's Global Score assessment will be done on Week 4, 8 and 12.

12.6 Study Hypothesis

The Hypothesis of the study is UB0316 as an adjuvant will bring a change in the hemoglobin A1c, as compared to placebo.

12.7 Sample Size Consideration

12.7.1 Sample Size Justification and power calculation

Sample size justification:

In justifying the sample size, we have made the assumptions which are given in below table for primary endpoint.

The calculation to justify the sample size is based on the need to assure the study has a sufficient probability to detect the presence of proportion difference.

Up to 92 patients are required to be screened and minimum 70 patients required evaluating the primary endpoint, which will provide 80% power to reject the null hypothesis (H_0 =Test – Placebo=0 verses H_a =Test – Placebo≠ 0) when the true overall mean difference is minimum 1.03 with a standard deviation of 1.5 at a significant level of 0.05.The sample size calculation was performed using SAS®9.4.

**Sample size for Clinical trial**	
Design	Double-blinded, Randomized, Placebo-Control.
Variance Estimate	1.5
Level of significance	0.05
clinical difference	1.03 difference in hemoglobin A1c
Expected power	80%
Other information as required to calculate sample size (e.g. cure rate, number of group)	Reduction of hemoglobin A1c (Baseline and week 12)
Expected Number of subjects study:	70
20% of dropouts	14
10% of Screen-failure	7
Total number of subjects required for the study	91+1=92 (Added 1 make even number for equal assignment)

12.8 PARTICIPANT ENROLLMENT AND FOLLOW – UP

Patients' enrollment and the follow-up will be conducted according to the point number 4 of the protocol.

12.9 PLANNED INTERIM ANALYSIS

Interim analysis will be conducted after 75% patients completing the study.

12.10 FINAL ANALYSIS PLAN

The patients completing all the visits of the study will be considered for the final analysis and will be considered as per protocol population. The patients discontinuing due to lost to follow up will be considered under ITT and the record of the same will be maintained in CRF. Missing values will be replaced by a Analysis method that will be mentioned in the appendix F. Data will be analyzed for per protocol analysis (PPA) to test for effects of treatment adherence. Details of statistical procedure i.e. the tables, listings and figures shall be described in the Statistical Analysis Plan.

13. DATA HANDLING AND RECORD KEEPING

Then data will be handled by the CRO and access to the database would be given to the members of the CRO and Biostatistician. Records will be entered in the Excel sheet, configured and reconcile with SAS software for statistical calculations. In the data sheet, patient number will be entered. The data sheet will be locked by a secured password. After entering the data the data will be analyzed, validated, cleaned and freeze for submission to the regulatory authorities. An excel sheet of freeze clinical data will be sent to the sponsors. SOP of the system will be maintained .An unambiguous subject identification that allows identification of all the data reported for each subject will be used.

The Source Data Verification Sheet (SDV, The OPD paper or any other document which reveals about the history of the patient in relation to disease and vital signs and physical symptoms) will be collected from each subject and will be noted in the CRF, for its accuracy and consistency. The SVD should be signed and dated by the investigator or the General Physician. The Physical and laboratory examination data will be entered in the CRF by the Investigator or the physician with date and signatures on it. This will in turn be monitored by the monitor visiting the site. The laboratory and other efficacy related data.

This above mentioned activities will be performed under and in accordance with ICH GCP guidelines.

13.1 DATA MANAGEMENT RESPONSIBILITIES

13.1.1 DATA MANAGEMENT ROLES AND RESPONSIBILITIES OF IHS-

IHS will design the manual CRF, Logs relating to data management. Data will be entered manually in the CRF s by the investigator during patients visit. After entering the data into the Physical CRF, discrepancies will be checked and resolved and the data will be entered into electronic case report form. The data entered by the Investigator will be sent for discrepancy management, where Clinical monitor will generate Data clarification in case of discrepancies to the investigator. QC personnel will perform the internal audit. Data entry will be performed after discrepancy management. The data would be entered in to the excel sheet. Double data entry would be done to check for verification and reconciliation of data. Preparation of Interim report will be done. Missing data will be imputed by satisfactory statistical method and will be mentioned later in Appendix F. Before sending for statistical analysis, accuracy and completeness of the data will be checked by QA personnel and data base will be locked and sent for statistical analysis. After preparation of statistical report, IHS will prepare Clinical report of the study.

13.1.2 Roles and Responsibilities of Sponsor-

1. Review of the trial data
2. Maintaining Quality Assurance and Quality control systems
3. Periodic access to the progress of clinical trials
4. Assign a Medical advisor for trial related medical questions and problems.

13.1.3 Roles and Responsibilities of the Investigator

1. Entering data in the Paper CRF
2. Maintenance of Source Verification Document.

13.2 Data Capture Methods

The data of each Subject/ individual will be captured in the paper CRF and E-CRF.

13.2.1 Types of Data

The data of each Subject/ individual will be captured in the paper CRF and will be collected each time the subject makes a visit this is done till he/she have not completed the study/ made the last visit. Only after the completion of the study at each center, this study data will be collected from the various centers where the study has been conducted, the data will be recorded, cleaned, analyzed and processed.

Similarly the data will be recorded in a form of E-CRF in "CASTOR EDC"; Online data collection software for medical research purpose. It records & saves data and provides a reliable back up and source trail for paper CRF in form of electronic data. The data from the paper CRF will be entered into the E-CRF as the study progresses in similar time frame.

The Expected timeframe for the completion of the study is from 6 months to 1 year, after which the data is collected from each center and recorded with the CRO or with the sponsor. The CRF's have to be submitted only after the last patient has made the last visit (As per each center). The Equipment that are required for capturing the data are paper CRF and Diaries (if required).

Data reported on the CRF should be consistent with the source documents. Any changes in the CRF should be dated, initialed and should explain the reason for the changes.

13.2.2 Study Records Retention

The Investigator should maintain all the records of the study for a minimum of 5 years or even more as per the type of the study, since the trial is in its Phase II stage, and is yet to get approved for post marketing studies/ Phase IV trials, Which will take another 5 years or even perhaps more than that for the product to get its approval to be marketed in the market.

The investigator or the Institution should ask for the permission from the sponsor before destruction of the records of the Investigational product and should have a look at the agreement signed between the IHS and the sponsor.

13.3 Protocol Deviations

13.3.1 Protocol Deviation/ Violation Reporting-

Definition of Deviation/ Violation- An unanticipated or an unintentional divergence or departure from the expected conduct of the approved study that is not consistent with current research protocol, consent document or study addenda.

The investigator should consider deviation and should complete the deviation from, in order to eliminate an immediate hazard(s) to trial subjects without prior IRB/IEC approval / favorable opinion. However, prior to implement deviation, investigator should inform about the deviation to the sponsor for agreement and in association of sponsor.

a. to the IRB/ IEC for review and approval/ favorable opinion

b. to the regulatory authority(ies)

As soon as possible, the implemented deviation or change, the reasons for it, and, if appropriate, the proposed protocol amendment(s) should be submitted to IRB/IEC.

1. The deviation has harmed or posed a significant or substantive risk of harm to the research subject.

- A research subject received the wrong treatment or incorrect dose.
- A research subject met withdrawal criteria during the study but was not withdrawn.
- A research subject received an excluded medication.

2. The deviation compromises the scientific integrity of the data collected for the study.

- A research subject was enrolled but does not meet the protocol's eligibility criteria.
- Failure to treat research subjects per protocol procedures that specifically relate to primary efficacy outcomes. (if it involves patient safety it meets the first category above)
- Changing the protocol without prior IEC approval.
- Inadvertent loss of samples or data.

3. The deviation is a willful or knowing breach of human subject protection regulations, policies, or procedures on the part of the investigator(s).

- Failure to obtain informed consent prior to initiation of study-related procedures
- Falsifying research or medical records.
- Performing tests or procedures beyond the individual's professional scope or privilege status (credentialing)

4. The deviation involves a serious or continuing noncompliance with federal, state, local or institutional human subject protection regulations, policies, or procedures.

- Working under an expired professional license or certification
- A breach of confidentiality
- Inadequate or improper informed consent procedure.

Minor Protocol Deviation- A minor protocol deviation is any change, divergence, or departure from the study design or procedures of a research protocol that has not been approved by the IEC and which DOES NOT have a major impact on the subject's rights, safety or well-being, or the completeness, accuracy and reliability of the study data

14. REFERENCES:

1. The pathogenesis and pathophysiology of type 1 and type 2 diabetes mellitus Ozougwu, J. C., Obimba, K. C. , Belonwu, C. D. , and Unakalamba, C. B. (2013)
2. A. J. Scheen. Pathophysiology of type 2 diabetes. (2003)
3. http://www.medicalnewstoday.com/info/diabetes
4. Gut microbiota, probiotics and diabetes Aline Corado Gomes, Allain Amador Bueno, Rávila Graziany, Machado de Souza and João Felipe Mota. Pub : Nutrition journal (2014)
5. Beyond gut microbiota: understanding obesity and type 2 diabetes Eva Lau, Davide Carvalho, Cidália Pina-Vaz, José-Adelino Barbosa, Paula Freitas. 2015 Jul-Sep; 14(3):358-69.
6. http://www.idf.org/complications-diabetes
7. Misra, A.; Khurana, L. Obesity related non-communicable diseases: South Asians vs White Caucasians. Int. J. Obes. 2011, 35, 167–187.
8. Misra, A.; Chowbey, P.K.; Makkar, B.M.; Vikram, N.K.; Wasir, J.S.; Chadha, D.; Joshi, S.R.; Sadikot, S.; Gulati, S.; Munjal, Y.P. Consensus statement for diagnosis of obesity, abdominal obesity and the metabolic syndrome for Asian Indians and recommendations for physical activity, medical and surgical management. J. Assoc. Physicians India 2009, 57, 163–170.
9. How Do We Define Obesity and Overweight? Obesity and Overweight. Available online: http://www.who.int/dietphysicalactivity/media/en/gsfs_obesity.pdf (accessed on 6 May 2013).
10. IDF Worldwide Definition of the Metabolic Syndrome. Available online: http://www.idf.org/ metabolic-syndrome (accessed on 29 November 2012).
11. National Cholesterol Education Program (NCEP) Expert Panel on Detection, Evaluation, and Treatment of High Blood Cholesterol in Adults (Adult Treatment Panel III). Third Report of the National Cholesterol Education Program (NCEP) Expert Panel on Detection, Evaluation, and Treatment of High Blood Cholesterol in Adults (Adult Treatment Panel III) final report. Circulation 2002, 106, 3143–3421.
12. The current state of diabetes mellitus in India Seema Abhijeet Kaveeshwar and Jon Cornwall. (2014)
13. Cani PD, Neyrinck AM, Fava F, Knauf C, Burcelin RG, et al. Selective increases of bifidobacteria in gut microflora improve high-fat-diet-induced diabetes in mice through a mechanism associated with endotoxaemia. Diabetologia 50: 2374–2383. (2007)
14. Misra A, Khurana L. Obesity-related non-communicable diseases: South Asians vs White Caucasians.Int J Obes (Lond) 2011;35(2):167–87

15. Gut Microbiota in Human Adults with Type 2 Diabetes Differs from Non-Diabetic Adults. Nadja Larsen ,Finn K. Vogensen,Frans W. J. van den Berg, Dennis Sandris Nielsen,Anne Sofie Andreasen, Bente K. Pedersen, Waleed Abu Al-Soud, Søren J. Sørensen, Lars H. Hansen, Mogens Jakobsen. PLoS ONE 5(2): e9085. doi: 10.1371/journal.pone.0009085
16. https://labtestsonline.org/understanding/analytes/a1c/tab/test/
17. Matsuzaki, T., Nagata, Y.,Kado, S., Uchida, K.,Hashimoto, S. andYokokura, T. Effect of oral administration of *Lactobacillus casei* on alloxan-induced diabetes in mice. *APMIS* **105**,637–642. (1997b)
18. Tabuchi, M., Ozaki, M.,Tamura, A., Yamada, N.,Ishida, T., Hosoda, M. and Hosono, A. Antidiabetic effect of *Lactobacillus* GG in streptozotocin-induced diabetic rats. *Biosci Biotechnol Biochem* **67**, 1421–1424. (2003)
19. S.I. Yun,H.O. Park,J.H. Kang. Effect of *Lactobacillus gasseri* BNR17 on blood glucose levels and body weight in a mouse model of type 2 diabetes. (2009)
20. Camila Moroti, Loyanne Francine Souza Magri, Marcela de Rezende Costa, Daniela CU Cavallini1 and Katia Sivieri. Effect of the consumption of a new symbiotic shake on glycemia and cholesterol levels in elderly people with type 2 diabetes mellitus. (2012)
21. Shirai K. Obesity as the core of the metabolic syndrome and the management of coronary heart disease. Curr Med Res Opin. 2004;20:295-304
22. Hariom Yadav, M.Sc. Shalini Jain, M.Sc.P.R. Sinha, M.Sc., Ph.D. Antidiabetic effect of probiotic dahi containing Lactobacillus acidophilus and Lactobacillus casei in high fructose fed rats. Nutrition (2006)
23. Ramachandran A. Epidemiology of type 2 diabetes in Indians. Indian J Med Res 125, ,pp 217-230, (March 2007)
24. Williams Nancy Toedter. Probiotics. American Journal of Health-System Pharmacy; 2010;67(6):449-458.
25. Cani PD, Neyrinck AM, Fava F, Knauf C, Burcelin RG, et al. Selective increases of bifidobacteria in gut microflora improve high-fat-diet-induced diabetes in mice through a mechanism associated with endotoxaemia. Diabetologia 50: 2374–2383. (2007)
26. http://www.diabetes.org/living-with-diabetes/treatment-and-care/blood-glucose-control/a1c/#sthash.ImCkORwR.dpuf
27. Diagnosis, epidemiology and pathogenesis of diabetes mellitus: an update for psychiatrists. (Holt 2004)
28. Nagpal J, Kumar A, Kakar S, Bhartia A. The development of 'Quality of Life Instrument for Indian Diabetes Patients (QOLID): A Validation & Reliability study in Middle and Higher income Groups; JAPI Vol 58; (May 2010)

15. SUPPLEMENTS/APPENDICES

15.1 APPENDIX A: SCHEDULE OF PROCEDURES AND EVALUATIONS

Sr. No	Tests & Assessments	Screening Baseline (Day 0 ± 5 days)	Visit 1 (Week 4 ± 5 days)	Visit 2 (Week 8 ± 5 days)	Visit 3 (Week 12± 5 days)
1.	Screening	X	-	-	-
2.	Consent	X	-	-	-
3.	Enrollment	X	-	-	-
4.	Medical History	X	-	-	-
5.	Vitals	X	X	X	X
6.	Systemic examination	X	X	X	X
7.	TRF for Blood Test	X	-	X	-
8.	Primary efficacy parameters Measurement	X	-	-	X
9.	Drug Dispensing	X	X	X	-
10.	Primary efficacy parameters	X	-	-	X
11.	Drug Compliance	-	X	X	X
12.	Patient Memory Card	X	X	X	X
13.	Quality of Life score	X	X	X	X
14.	Adverse event monitoring	X	X	X	X
15.	Physician's and Subject's Global Assessment	-	X	X	X

15.2. APPENDIX B: QOL QUESTIONNAIRE

Sr. No	Questions	Response				
	**Role Limitation Due to Physical Health**	1	2	3	4	5
1.	How often do you miss your work because of your diabetes?	Always	Frequently	Often	Sometimes	Never
2.	A person with diabetes has the requirement of adhering to a schedule for eating and taking regular medication. How often does this affect your work?	Always	Frequently	Often	Sometimes	Never
3.	How often does diabetes affect your efficiency at work?	Always	Frequently	Often	Sometimes	Never
4.	How often do you find diabetes limiting your social life?	Always	Frequently	Often	Sometimes	Never
5.	To what extent do you avoid traveling (business tour, holiday, general outings) because of your diabetes?	A lot	Highly	Little	Very little	Not at all
6.	Compared to others of your age are your social activities (visiting friends/partying) limited because of your diabetes?	A lot	Highly	Little	Very little	Not at all
**Physical Endurance**						
1.	How often in last three months has your overall health problems limited the kind of vigorous activities you can do like lifting heavy bags/objects, running, skipping, jumping?	Always	Frequently	Often	Sometimes	Never
2.	How often in last three months has your overall health problems limited the kind of moderate activities you can do like moving a table, carrying groceries or utensils?	Always	Frequently	Often	Sometimes	Never
3.	How often in last three months has your overall health problems limited you from walking uphill or climbing 1-2 floors?	Always	Frequently	Often	Sometimes	Never
4.	How often in last three months has your overall health problems limited you from walking 1-2 km at a stretch?	Always	Frequently	Often	Sometimes	Never
5.	How often in last three months has your overall health problems limited you from bending, squatting, or turning?	Always	Frequently	Often	Sometimes	Never
6.	How often in last three months has your overall health problems limited you from eating, dressing, bathing, or using the toilet?	Always	Frequently	Often	Sometimes	Never
	**General Health?**					
1.	In general would you say your health is?	Poor	Fair	Good	Very Good	Excellent
2.	How well are you able to concentrate in everything like working, driving, reading etc.?	Not at all	Little	Moderate	very much	Extreme
3.	How many times in the past three months have you had fatigue/ felt very tired?	Always	Frequently	Often	Sometimes	Never
	**Treatment Satisfaction**					
1.	How satisfied are you with your current diabetes treatment?	Very dissatisfied	Moderately dissatisfied	Neither satisfied nor dissatisfied	Moderately Satisfied	Very Satisfied
2.	How satisfied are you with amount of time it takes to manage your diabetes?	Very dissatisfied	Moderately dissatisfied	Neither satisfied nor dissatisfied	Moderately Satisfied	Very Satisfied
3.	How satisfied are you with the amount of time you spend getting regular check-up's (once in 3 months)?	Very dissatisfied	Moderately dissatisfied	Neither satisfied nor dissatisfied	Moderately Satisfied	Very Satisfied
4.	A person with diabetes needs to exercise for 35-45 min, 4 times a week. Keeping this in mind how satisfied are you with the time you spend exercising?	Very dissatisfied	Moderately dissatisfied	Neither satisfied nor dissatisfied	Moderately Satisfied	Very Satisfied

*Nagpal j, Kumar A, Kakar S, Bhartia A. The development of 'Quality of Life Instrument for Indian Diabetes Patients (QOLID) : A Validation & Reliability study in Middle and Higher income Groups. May 2010; JAPI Vol 58.

Sr. No	Questions	Response				
	**Symptom Botherness**	1	2	3	4	5
1.	How many times in the past three months have you felt excessive hunger?	Always	Frequently	Often	Sometimes	Never
2.	How many times in the past three months have you had frequent urination related to diabetes management?	Always	Frequently	Often	Sometimes	Never
3.	How many times in the past three months have you had frequent urination related to diabetes management?	Always	Frequently	Often	Sometime	Never
	**Emotional/Mental Health**					
1.	How satisfied are you with yourself?	Very dissatisfied	Moderately dissatisfied	Neither satisfied nor dissatisfied	Moderately Satisfied	Very Satisfied
2.	How satisfied are you with your personal relationships (family, friends, relatives and known tos)?	Very dissatisfied	Moderately dissatisfied	Neither satisfied nor dissatisfied	Moderately Satisfied	Very Satisfied
3.	How satisfied are you with the emotional support you get from your friends and family?	Very dissatisfied	Moderately dissatisfied	Neither satisfied nor dissatisfied	Moderately Satisfied	Very Satisfied
4.	How often are you discouraged by your health problems?	Always	Frequently	Often	Sometimes	Never
5.	All people want to fulfil certain roles and lead their lives in a purposeful manner. To what extent do you feel that you have been able to lead your life in the same way?	Not at all	Little	Moderate	very much	Extreme
	**Diet Satisfaction**					
1.	How often do you feel because of your diabetes a restriction in choosing your food when eating out?	Always	Frequently	Often	Sometimes	Never
2.	How often do you eat the food items that you shouldn't, in order to hide the fact that you are having diabetes.	Always	Frequently	Often	Sometimes	Never
3.	As you have diabetes, how much choice do you feel you have in eating your meals or snacks away from home e.g. if you go in a party and there is a buffet where there are also a lot of fried snacks and desserts would you be able to make enough choice?	No choice	very little	Little	Enough	A lot

Sr. No	Questions	Response			
	**Financial Worries**	1	2	3	4
1.	What do you think about the cost involved in your management of diabetes?	Very expensive	Little Expensive	Reasonable	Not at all expensive

		Response				
		1	2	3	4	5
2.	To what extent has your priority of expenditure shifted towards diabetes management?	A lot	Highly	Little	Very little	Not at all
3.	To what extent has your family budget got affected by the expenses related to the management of diabetes?	A lot	Highly	Little	Very little	Not at all
4.	To what extent has your diabetes limited your expenditure on other aspects of life (Movies, outings, parties etc.)?	A lot	Highly	Little	Very little	Not at all

*Nagpal J, Kumar A, Kakar S, Bhartia A. The development of 'Quality of Life Instrument for Indian Diabetes Patients (QOLID) : A Validation & Reliability study in Middle and Higher income Groups. May 2010; JAPI Vol 58.

Above questionnaire determines the impact of diabetes on the quality of life of an individual. It was developed and validated by J. Nagpal, *Et. al*. May 2010;JAPI Vol 58.

Questionnaire consists of 34 questions spanning over 8 domains associated with daily life. All domains have high internal consistency and lower level of refusal rate. Standard Likert Scale is used across all questions. In the conclusion it states" QOLID is a reliable valid and sensitive tool for assessment of diabetes specific Quality of life in Indian Patients".

15.3 APPENDIX C: CONSENT FORM

Patient Consent Form

Protocol ID: IHS/UBL/03/16

Study Title: A double blind, randomized, placebo controlled study to determine the safety and efficacy of UB 0316 as an adjuvant for 12 weeks in type 2 diabetes mellitus.

**Subject's Full Name:**	
**Date of Birth:**	**Qualification:**
**Occupation:**<br>Student/self -employed/service/housewife/other (please tick as appropriate)	
**Subject's Address:**	
**Contact Details:**	

I confirm that I have read and understood the information document dated ___________ for the above study and have had the opportunity to ask questions.

1. I understand that my participation in the study is voluntary and that I am free to withdraw at any time, without giving any reason and without my medical care or legal rights being affected.

2. I understand that the sponsor of the clinical trial/project, others working on the Sponsor's behalf, the Ethics Committee and the regulatory authorities will not need my permission to look at my health records both in respect of the current study and any further research that may be conducted in relation to it, even if I withdraw from the trial. However, I understand that my Identity will not be revealed in any information released to third parties or published.

3. I agree not to restrict the use of any data or results that arise from this study provided such a use is only for scientific purpose(s).

4. I permit the use my stored sample (Blood) for future research. Yes [ ] No [ ]

5. I agree to take part in the above study.

**Name of the Patient**	**Date**	**Signature of the Patient**

**Name of the Witness**	**Date**	**Signature of the Witness**

**Name of the Investigator**	**Date**	**Signature of the Investigator**

15.4 APPENDIX D: INVESTIGATORS NAME AND SITES

Principal Investigator: Dr.Anirudh Tripathi

Life Veda Treatment and Research Centre
7/6, Century Quarters,
Next to Doordarshan towers P.B.road,
Worli, Mumbai-400030

Co-Investigator: Dr. Satyavrat Nanal
Co-Investigator: Dr. Mahesh Talekar
Nanal Ayurvedic Clinic.
102, Anand bhuvan, Gorewadi,
Opposite MTNL Colony, Near Matunga Road Railway station,
Mahim west, Mumbai – 400016.

15.5 APPENDIX E: PATIENT MEMORY CARD

Patient Memory Card

PROTOCOL NUMBER: **IHS/UBL/03/16**
TITLE: A double blind, randomized, placebo controlled study to determine the safety and efficacy of **UB0316** as an adjuvant for 12 weeks in type 2 diabetes mellitus.

**Subject Initials:**				**Subject ID**				

Dose: 2 UB0316 capsule/per orum/per day for 12 Weeks

Visit Schedule:

**Visit Name**	**Date and Day**	**Blood Sample Submission Date**	In the event of you unable to visit on scheduled date, you will have to come for your follow up within (after/before) 5 days of your scheduled visit.
**Screening Visit & Baseline Visit**		XX	
**Follow Up Visit 1**			
**Follow Up Visit 2**			
**Follow Up Visit 3**		XX	

15.6 APPENDIX F: STATISTICAL ANALYSIS PLAN

STUDY HYPOTHESIS

The Hypothesis of the study is UB0316 as an adjuvant will bring a change in the hemoglobin A1c, as compared to placebo.

SAMPLE SIZE CONSIDERATION

SAMPLE SIZE JUSTIFICATION AND POWER CALCULATION

Sample size justification:

In justifying the sample size, we have made the assumptions which are given in below table for primary endpoint.

The calculation to justify the sample size is based on the need to assure the study has a sufficient probability to detect the presence of proportion difference.

Up to 92 patients are required to be screened and minimum 70 patients required evaluating the primary endpoint, which will provide 80% power to reject the null hypothesis (H0 =Test – Placebo=0 verses Ha =Test – Placebo≠ 0) when the true overall mean difference is minimum 1.03 with a standard deviation of 1.5 at a significant level of 0.05.The sample size calculation was performed using SAS®9.4.

**Sample size for Clinical trial**	
Design	Double-blinded, Randomized, Placebo-Control.
Variance Estimate	1.5
Level of significance	0.05
clinical difference	1.03 difference in hemoglobin A1c
Expected power	80%
Other information as required to calculate sample size (e.g. cure rate, number of group)	Reduction of hemoglobin A1c (Baseline week 4, week 8 and week 12)
Expected Number of subjects study:	70
20% of dropouts	14
10% of Screen-failure	7
Total number of subjects required for the study	91+1=92 (Added 1 make even number for equal assignment)

PARTICIPANT ENROLLMENT AND FOLLOW – UP

Patients' enrollment the follow-up will be conducted according to the point number 4 of the protocol.

PLANNED INTERIM ANALYSIS

Interim analysis will be conducted after 75% patients completing the study.

FINAL ANALYSIS PLAN

The patients completing all the visits of the study will be considered for the final analysis and will be considered as per protocol population. The patients discontinuing due to lost to follow up will be considered under ITT and the record of the same will be maintained in CRF. Missing values will be replaced by a method that will be mentioned in the appendix F. Data will be analyzed for per protocol analysis (PPA) to test for effects of treatment adherence. It will be mentioned elaborately and distinctively in SAP (Statistical Analysis plan).
